# Ligand Hydrogenation during Hydroformylation Catalysis Detected by In Situ High-Pressure Infra-Red Spectroscopic Analysis of a Rhodium/Phospholene-Phosphite Catalyst †

**DOI:** 10.3390/molecules29040845

**Published:** 2024-02-14

**Authors:** José A. Fuentes, Mesfin E. Janka, Aidan P. McKay, David B. Cordes, Alexandra M. Z. Slawin, Tomas Lebl, Matthew L. Clarke

**Affiliations:** 1 EaStCHEM School of Chemistry, University of St Andrews, Purdie Building, North Haugh, St Andrews KY16 9ST, UK; jaf14@st-andrews.ac.uk (J.A.F.); apm31@st-andrews.ac.uk (A.P.M.); dbc21@st-andrews.ac.uk (D.B.C.); amzs@st-andrews.ac.uk (A.M.Z.S.); tl12@st-andrews.ac.uk (T.L.); 2 Eastman Chemical Company, 200 South Wilcox Drive, Kingsport, TN 37660, USA

**Keywords:** hydroformylation, phosphacycles, homogeneous catalysis, rhodium, in situ spectroscopy, regioselectivity

## Abstract

Phospholane-phosphites are known to show highly unusual selectivity towards branched aldehydes in the hydroformylation of terminal alkenes. This paper describes the synthesis of hitherto unknown unsaturated phospholene borane precursors and their conversion to the corresponding phospholene-phosphites. The relative stereochemistry of one of these ligands and its Pd complex was assigned with the aid of X-ray crystal structure determinations. These ligands were able to approach the level of selectivity observed for phospholane-phosphites in the rhodium-catalysed hydroformylation of propene. High-pressure infra-red (HPIR) spectroscopic monitoring of the catalyst formation revealed that whilst the catalysts showed good thermal stability with respect to fragmentation, the C=C bond in the phospholene moiety was slowly hydrogenated in the presence of rhodium and syngas. The ability of this spectroscopic tool to detect even subtle changes in structure, remotely from the carbonyl ligands, underlines the usefulness of HPIR spectroscopy in hydroformylation catalyst development.

## 1. Introduction

**Figure 1:**
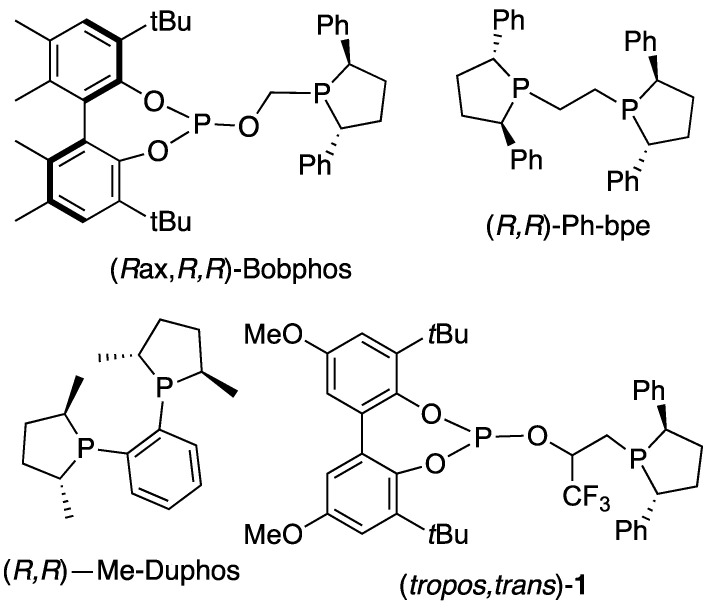
Structures of some phospholanes used in homogeneous catalysis.

Phosphacycles are widely applied ligands in several areas of homogenous catalysis [1,2,3,4,5,6,7,8,9,10,11], and hence new examples of phosphacycles are important in both main group chemistry and catalysis disciplines. Rhodium complexes of enantiomerically pure 2,5-disubstituted phospholanes, such as Me-DUPHOS (Figure 1), are produced at the kilogram scale and are applied industrially, whilst other related bis-phospholanes have many applications as catalysts in asymmetric synthesis [2,8,9]. The 2,5-diarylphospholano motif is present in the widely used ligand Ph-BPE (Ph-BPE = phenyl, bis-phospholano-ethane, Figure 1) [10,11] and additionally in phospholane-phosphite ligands, such as Bobphos (Figure 1) [11]. The latter confer very unusual branched regioselectivity up to 6:1 in the hydroformylation of a range of unbiased terminal ‘alkyl-alkenes’, a type of substrate that normally forms linear aldehydes [12,13,14,15]. Phospholane-phosphites of this type have also recently been found to be preferred ligands for certain enantioselective arylation reactions using arylboron reagents [16,17].

These catalysts were originally developed for producing high-value, branched, enantiomerically pure aldehydes for pharmaceutical and fine chemical synthesis, but more recently we have been working on a programme exploring the possibility of developing a large-scale selective route to *iso*-butanal from propene. We reported that several milestones were reached, namely, (i) the use of unusual process conditions to improve the *iso*-butanal regioselectivity observed in the initial screening from around 60% up to around 80%, maintaining a selectivity of at least 65% even at high temperatures, at which industrially acceptable rates were observed [14]; (ii) the redesign of the ligand to obtain structures such as **1** (Figure 1) to confer stability at high temperatures for elongated periods of time [18]. These solutions to significant hurdles delivered stable and selective catalysts that operated with no decomposition in experiments lasting over several days, producing kilogram amounts of products [18]. One issue, however, is that for such a high-volume speciality chemical like isobutanal, with a market of many thousands of tonnes, the ligand structure is quite complex, needing eight synthetic steps; simpler ligand structures that can produce catalysts with similar performance are of significant interest. 

**Scheme 1:**
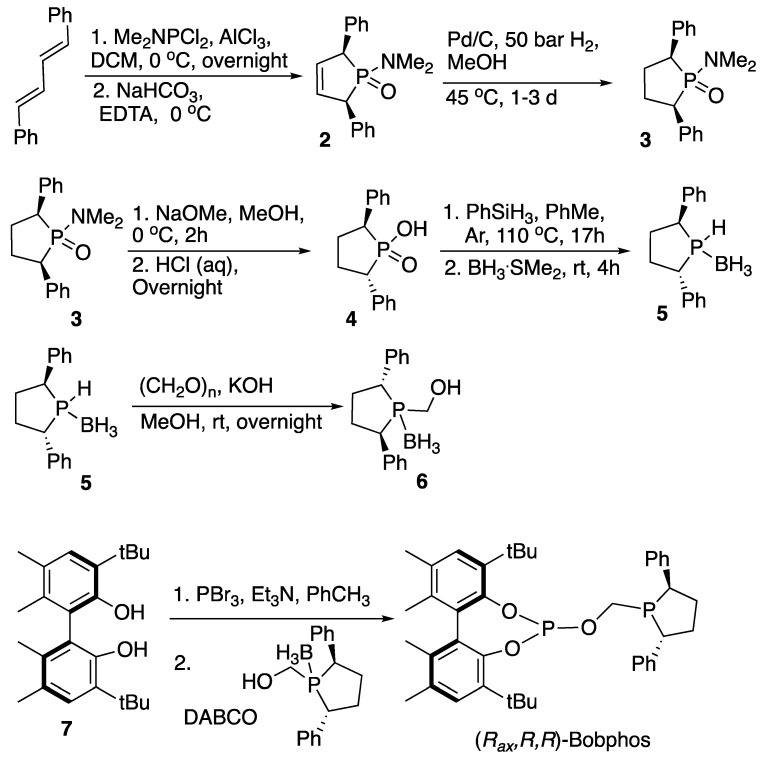
Synthesis of (*R_ax_*,*R*,*R*)-Bobphos.

Considering the synthetic route to Bobphos (Scheme 1) and the proposed origin of selectivity [19], it seemed plausible that an unsaturated *phospholene*-phosphite could be obtained with two fewer synthetic steps. It was unclear what the effect of the more rigid unsaturated ring and the different relative stereochemistry of the two phenyl groups would be, but we hoped that the molecule might be sufficiently similar to **1** to still favour branched aldehyde formation. Here, we describe the synthesis of this type of ligand and its precursors, before reporting on a study of the stability of their Rh catalysts. In situ high-pressure infra-red (HPIR) spectroscopy was discovered to be a sufficiently sensitive tool to detect the hydrogenation of the C=C bond in the phospholene under conditions relevant to hydroformylation catalysis.

## 2. Results and Discussion

**Scheme 2:**
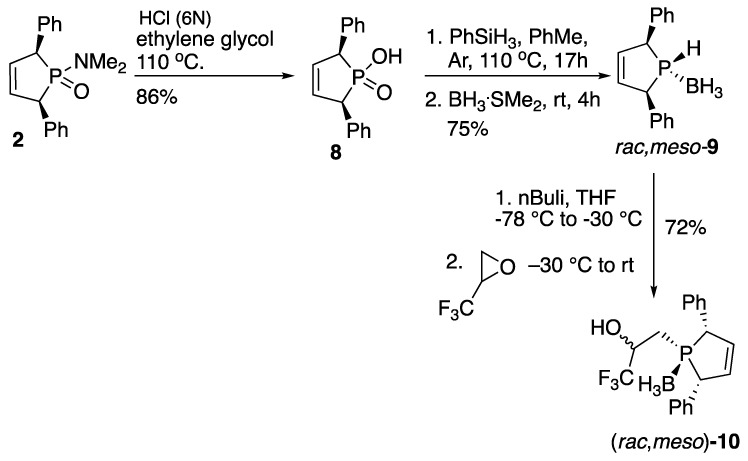
Synthesis of secondary phospholene borane **9** and the precursor to phospholene-phosphite ligands. (See Scheme 1 for synthesis of **2**).

To the best of our knowledge, phospholene ligands are very scarcely applied in transition metal catalysis or as ligands in general [20,21]. Phosphorous chemists have prepared a variety of these molecules over the years [20,21,22,23,24,25,26,27], primarily using a McCormack cycloaddition between dienes and phosphenium cations. Their main utility has been as precursors to other phosphacycles [22,23,24,25,26]. A secondary phospholene borane precursor to produce 2,5-diarylsubstituted phospholenes was consequently unknown, but was readily prepared in this study by simply omitting the C=C hydrogenation and *cis-trans* isomerisation steps that were previously used to prepare secondary phospholane borane, **5** [10,22]. The synthesis is shown in Scheme 2 and started from amino-phosphine oxide **2** obtained in one step from diphenylbutadiene, as shown in Scheme 1. The *cis–trans* isomerization and hydrogenation steps for the synthesis of **1** were omitted, making phospholenic acid **8** available in just two steps in place of four steps needed to make phospholanic acid **4**.

Phospholenic acid **8** was already described in the literature [24]. Reduction of acid **8** followed by protection with borane-dimethylsulfide afforded the corresponding secondary phospholene **9**. The preparation of the phospholene-containing fragment was achieved by deprotonation of secondary phospholene **9** with *n*-BuLi and attack of the resulting anion on the less substituted carbon of the commercially available 2-(trifluoromethyl)oxirane to obtain adduct **10** (Scheme 2). The NMR data suggested the presence of a single, racemic diastereomer and a single regioisomer in solution for compounds **9** and **10** [27]. The relative stereochemistry in phospholene borane **9** was investigated using 1D gs-NOESY. An NOE between hydrogens, three bonds apart in a five-membered ring, cannot be reliably used to assign the relative stereochemistry without a known standard to compare to. This is not only due to the likelihood of zero-quantum coherence effect (ZQC), but also due to a high chance of seeing an NOE signal even in the case of *anti* geometry. Whilst such an NOE would be expected to be weaker for an *anti* relationship between P-H and C-H, in the case of only one isomer, it would not be possible to assign the relative stereochemistry without knowing the NOE for the other isomer. Fortunately, in this case, the previously synthesised phospholane borane **5** has C-H bonds with both *syn* and *anti* relationships with the P-H bond; a 1D gs-NOESY spectrum of phospholane borane **5** (Supplementary Materials) showed the presence of both NOE effects and H *syn* to P-H and H *anti* to P-H relationships. There was a strong NOE signal for one of the CH α to P and a weak antiphase (ZQC) signal for the other CH, as expected (see Supplementary Materials). We found that 1D gs-NOESY of phospholene borane **9** only showed an antiphase (ZQC) artefact signal when irradiating *H*-P, due to scalar coupling between those spins (*J* = 8.2 Hz). Furthermore, a significant NOE of the *H*-P with one of the aromatic hydrogens (see Supplementary Materials) was observed, suggesting *anti* stereochemistry, as depicted in Scheme 2.

**Scheme 3:**
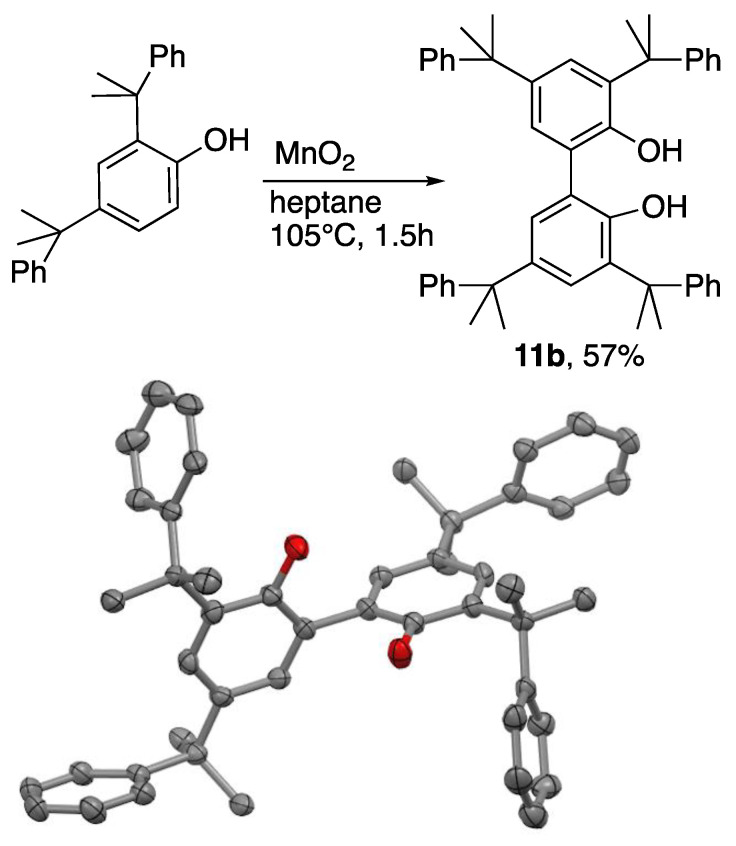
Synthesis and X-ray crystal structure of **11b** (thermal ellipsoid plot, 50% probability ellipsoids). Carbon atoms shown grey, oxygen red). Hydrogen atoms omitted for clarity.

The final step in the synthesis was the coupling between **10** and a chloro-phosphite. In addition to the main target **13a**, a bulkier phosphite fragment, **13b**, and a less electron-donating phosphite fragment, **13c**, were targeted for synthesis. The requisite diol, **11b**, for the bulkier ligand was not commercially available and was prepared using a classical oxidative coupling in the presence of MnO_2_ (Scheme 3).

Compound **11b** was reported previously in the literature [28]. Our sample of diol **11b**, prepared as in Scheme 3, resembled a pure compound with the expected NMR spectrum, but the NMR data did not fully match the literature [28], as our NMR spectrum contained significantly fewer peaks. Esguerra et al. attributed the unexpectedly high number of observed signals in their data to the presence of two conformational isomers. For whatever reason, the sample of **11b** we prepared did *not* show extra signals, possibly assignable to the unexplained lack of an extra conformational isomer or to a major impurity in the compound reported in reference [28] To completely confirm that our NMR data indicated the proposed structure of compound **11b**, we determined the structure of diol **11b** using X-ray crystallography. The crystal structure is shown in Scheme 3, confirming the structure of the diol prepared in Scheme 3. One feature that merits discussion concerns the twists between the rings. We found that biphenol **11b** possessed a smaller twist of 79.1(4)° than the one observed for diol **11a**, of 89.84(8)° [29]. In comparison, unsubstituted [1,1′-biphenyl]-2,2′-diol displayed a much smaller torsion angle of 48.71(5)°, probably due to the lack of substituents and the presence of intramolecular hydrogen bonding [30].

**Scheme 4:**
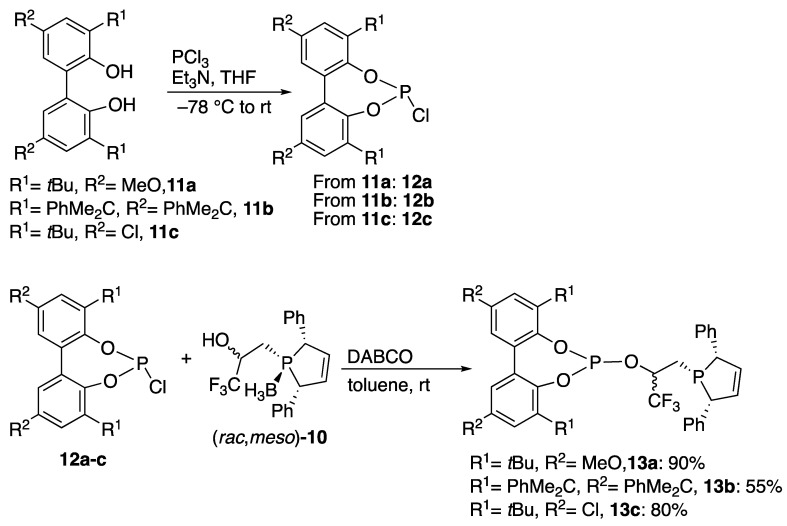
Phosphite coupling and deprotection gives the final phospholene-phosphite ligands.

Phosphite coupling to prepare the ligands **13a**–**13c** was achieved by synthesising the corresponding chlorophosphites from *tropos* diols **11a**–**11c**, and these were reacted directly with precursor **10** in the presence of 1,4-diazabicyclo-[2,2,2]-octane (DABCO, Scheme 4).

**Figure 2:**
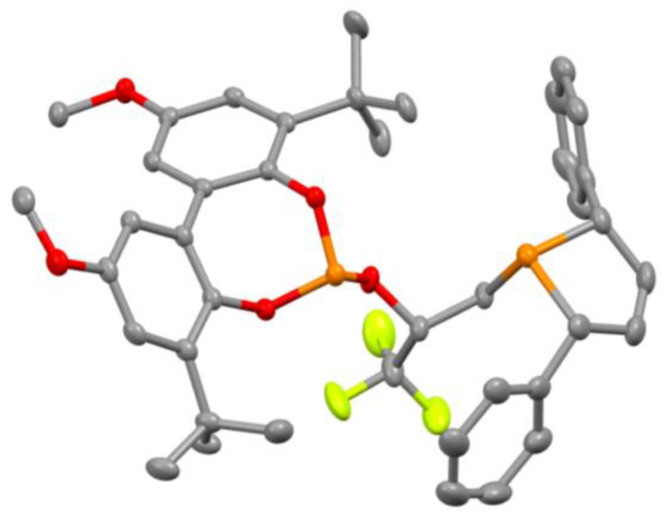
X-ray crystal structure of **13a** (thermal ellipsoid plot, 50% probability ellipsoids). Carbon atoms shown grey, oxygen red, fluorine green, phosphorus orange). Hydrogen atoms omitted for clarity.

In order to confirm the relative configuration of ligand **13a**, an X-ray crystal structure was obtained (Figure 2). In the structure, it can be observed that the lone pair on phosphorous is *anti* relative to the *meso-cis* phenyl groups. This relative stereochemistry for alkyl or P-H relative to phenyl was similar to the stereochemical assignment made for **9**. It is quite possible that, rather than the deprotonation–alkylation step occurring with retention of configuration at phosphorus, the deprotonated form of **9** might be configurationally unstable, with the most thermodynamically stable and/or most reactive stereoisomer of the anion leading to the observed stereochemistry in precursor **10**. Retaining the same stereochemistry in **10** and **13a** is as expected. Ligand **13a** (in racemic form) was, as expected, a mixture of the (*S_c_*, *meso-cis*) and (*R_c_*, *meso-cis*) isomers. 

It is worth mentioning that ligand **13a** contains a *tropos* diol, which, in solution, displays a rapid interconversion through the planar conformation. However, in the solid state, the diol settles in a preferred *atropos* conformation that depends on the chirality of the stereocentre with the CF_3_ substituent. The two isomers (enantiomers) observed were, therefore, (*S_c_*, *pseudo-R_ax_*, *meso-cis_ring_)* and (*R_c_*, *pseudo-S_ax_*, *meso-cis_ring_*), both with the P lone pair *anti* to the phenyl rings.

**Scheme 5:**
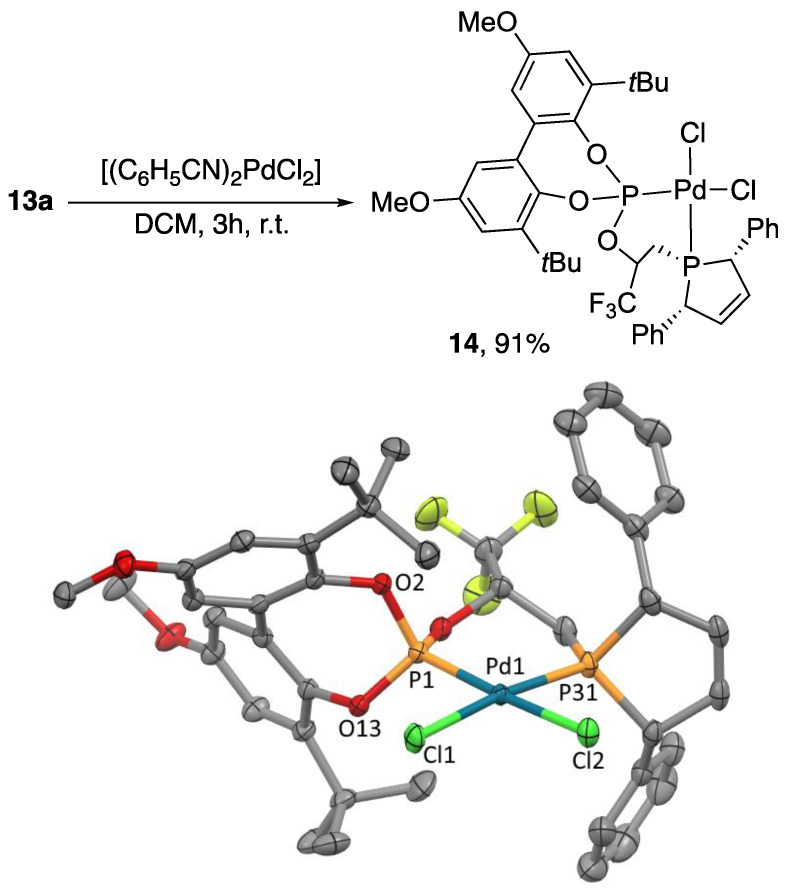
Synthesis and X-ray crystal structure of **14** (thermal ellipsoid plot, 50% probability ellipsoids). Carbon atoms shown grey, oxygen red, fluorine light green, phosphorus orange, chlroine green, palladium turquoise). Hydrogen atoms and chloroform solvates omitted for clarity.

Crystals of a rhodium complex derived from ligands **13a**–**c** were not available; so, one example of a Pd complex was prepared, which had the additional desirable feature of being comparable to the analogous Pd(L)Cl_2_ complex of Bobphos that was structurally characterised [19]. Complex **14** was prepared by reacting ligand **13a** with [PdCl_2_(PhCN)_2_]. and single crystals suitable for analysis were grown from chloroform. The X-ray crystal structure of [PdCl_2_(**13a**)]·3CHCl_3_ (**14**) (Scheme 5, Table 1) revealed a slightly distorted square planar geometry about palladium. The bidentate ligand **13a** occupied two coordination sites, with a P-Pd-P crystallographic bite angle of 96.22(2)°, enlarged by about 6° over the preferred 90° for this type of complex. This crystallographic bite angle was over 10° larger than the one in [PdCl_2_{(*S_ax_*,*S*,*S*)-Bobphos}]·2CHCl_3_ [19], which was largely due to the introduction of an extra carbon in the linker of **13a**. The Pd-Cl bonds were slightly shorter than in [PdCl_2_(*S_ax_*,*S*,*S*)-Bobphos)], and contrary to the Pd/Bobphos complex, the Pd-Cl bond *trans* to the phosphite, 2.3385(5) Å, was slightly longer than the Pd-Cl bond trans to the phosphine, 2.3271(5) Å. 

The twist observed between the phenol groups of 53.05(4)° in the free ligand **13a** was most similar to that in the unsubstituted biphenol, with the phospholene ring restricting the rotation between the phenol moieties, and remained quite far from perpendicular in the Pd complex at 50.07(6)° This was a narrower twist than that observed in the analogous complex derived from an atropos biphenol, [PdCl_2_(*S_ax_*,*S*,*S*)-Bobphos·2CHCl_3_], which was 66.8(4)°.

Hydroformylation catalysis continues to attract academic research interest and promote new discoveries in the industry [31,32,33,34]. Whilst the reaction is of interest across several sectors, the largest scale hydroformylation reaction practised is the Rh-catalysed hydroformylation of propene. Whilst linear selective reactions produce *n*-butanal, needed on a large scale, the formation of *iso*-butanal is of more recent interest, both as a conceptual challenge to reverse the innate preference of these reactions and since *iso*-butanal now has a very significant and increasing market [33,34,35]. This reaction has been the focus of a long-standing project in our laboratories, and branched selective hydroformylations (and hydroformylation with unusually low *n/iso* ratios) have been studied quite widely [36]. Until recently, class-leading results were generally to tilt the *n/iso* ratio; so, the *iso*-product was slightly in excess. Some reactions more similar to *iso*-selective reactions have been reported recently [14,18,35,36,37]. The performances of these new, more readily synthesised *phospholene-phosphite* ligands, **13a**–**13c**, were studied in the hydroformylation of propene and were compared to class-leading *phospholane-phosphite* **1** (Table 2). We previously reported the use of *n*-dodecane, a cheap non-volatile solvent, indicating it as a good solvent that can be used as an alternative to more expensive but preferred fluorinated solvents; dodecane was the solvent used in this study [18]. The catalysts originated from these ligands were preactivated by mixing [Rh(acac)(CO)_2_] and the corresponding ligand in dodecane and syngas at 90 °C before the vessel was brought to reaction temperature and then filled with the propene/syngas mixture, as we described in previous papers. Complete conversion was 1450 TON for reactions filled at 90 °C; so, the turnover measurements were performed at <50% conversion after 1 h, and the values obtained can be considered to be the average TOF for the first stage of the reaction. The time taken for this activation step was separately studied by in situ HPIR spectroscopy (Supplementary Materials) to ensure that the bands associated with the catalyst had grown to full intensity within the chosen activation times used in the catalysis experiments (30–45 min). The activation times for the phospholene-phosphite ligands **11a**–**11c** were found to be similar to those for ligand **1**. We were pleased to find that Rh/**13a** generated a branched selective catalyst, affording *iso*-butanal with a selectivity of 67.5% at 75 °C (Table 2, entry 2). The *iso*-selectivity of the new complex was lower than that of the complex obtained with Rh/**1** at the same temperature, (74.6%, Table 2, entry 3), but not by a huge margin. The stereochemical change to a *meso-cis* arrangement of the two phenyl groups seemed only to have a marginal impact on regioselectivity; this was far from obvious, given the various subtleties we observed during our work with phospholanes [14].

Improved turnover frequencies (TOFs) were obtained at 90 and 105 °C, and the loss of selectivity with temperature compared with that for Rh/**1** was also lower, allowing Rh/**13a** to have a similar *iso*-butanal selectivity to Rh/**1** at 105 °C (64.5% vs. 67.2%, Table 2, entries 6 and 7). The bulkier phosphite-phospholene **13b** did not improve the branched selectivity in the reaction, and there was a drop in activity (Table 2, entries 8–10). The introduction of a Cl atom in the backbone of the diol moiety in the phosphite (ligand **13c**), in order to make the phosphorus atom of the phosphite less basic, afforded the expected increase in the reaction rate (Table 2, entries 11–13). However, this did come at the cost of a drop in selectivity in the reaction.

**Figure 3:**
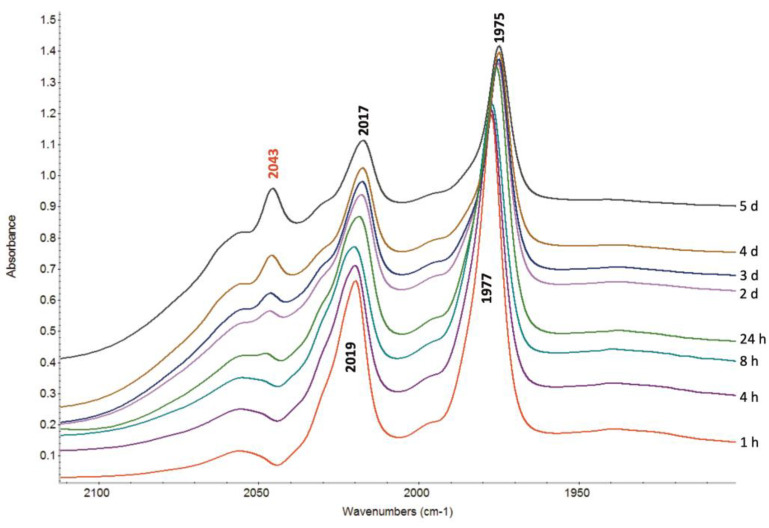
HPIR spectra of [RhH(CO)_2_**13a**]. Conditions: Rh/L = 1:1.25 (C_Rh_ 1 mM in dodecane), T = 90 °C, P = 20 bar, CO:H2 1:1. Colours represent spectra obtained at times given on right of figure.

Initially, an HPIR study was carried out to characterise the catalyst resting state and, as previously discussed, to investigate the time taken for the activation step (For discussion on the HPIR set-up, see ref. [38]). The presence of two main bands in the spectrum at 2019 and 1977 cm^−1^ and the asymmetric nature of the bands were consistent with an equatorial–axial (*ea*) coordination mode (Figure 3) [18,39]. We were very pleased to find that the stability, with respect to the decomposition of a range of unselective, unidentifiable species, was good: after 5 days at 90 °C, the IR bands for [RhH(CO)_2_(**13a**)] were characteristic of an eq–ax isomer of a complex of type [RhH(CO)_2_(**L**)] (Figure 3). However, a small shift was observed for both bands, from 2019 to 2017 and from 1977 to 1975 cm^−1^, after 24 h (Figure 3).

Given the lack of data on phospholene ligands in homogeneous catalysis, one aspect of interest to us at the outset of this study was if the alkene function in the phospholene was stable in hydroformylation conditions and whether in situ HPIR spectroscopy was a sensitive-enough tool to observe relatively remote changes in the catalyst relative to the carbonyl ligands on rhodium. It is possible the alkene could undergo hydroformylation, polymerisation, isomerisation or hydrogenation. Polymerisation was relatively unlikely from a reactivity perspective and would be revealed by changes in physical properties. Hydroformylation should lead to new carbonyl bands in the IR and formyl protons when investigated by NMR. Detection of the catalyst resting state by NMR spectroscopy was therefore performed.

**Figure 4:**
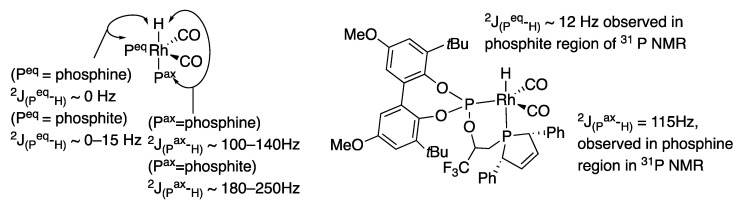
(**Left**) Diagnostic coupling ranges for phosphorus ligands bound in axial and equatorial sites in trigonal bipyramidal rhodium hydride complexes. (**Right**) Proposed structure and coupling constants observed for [RhH(CO)_2_(**13a**)].

We previously found all of the [RhH(CO)_2_(**L**)] complexes from bidentate ligands related to Bobphos (and to diphosphites) to be stable for several hours, enabling NMR characterisation under 1 atm of syngas; so, the desired complex, [RhH(CO)_2_(**13a**)] was generated in a pressure vessel and then sampled for NMR interrogation. For a broader discussion on the expected fluxionality for a Rh complex of an asymmetrically substituted bidentate ligand, see References [18,40]. However, the key parameter is the magnitude of ^2^*J*_P-H_, since ^2^*J*_P-H_ for H-*trans*-P is known to be much larger (100–250 Hz) than for a *cis* coupling (10–20 Hz) (Figure 4). In addition, phosphites have significantly larger coupling constants than phosphines (by a factor of nearly 2; hence, a complex with phosphite *trans* to H would have ^2^*J*_P-H_ of around 200 Hz). Intermediate values (e.g., ^2^*J*_P-Hcis_ of around 50 Hz), can sometimes be observed when there is a rapidly interconverting mixture of complexes with phosphite *trans* to H and complexes with phosphine *trans* to H, but this was not significant here. Thus, it was possible to measure two ^2^*J*_P-H_ coupling constants from the hydride region of the ^1^H NMR spectrum, which were ~115 Hz and either 10 or 12 Hz (^1^*J*_Rh-H_ was also 10 or 12 Hz and was not distinguishable from the very similar ^2^*J*_P-H cis_). Comparing the ^31^P NMR and ^31^P{^1^H} NMR spectra clearly showed that the phosphite only possessed a small ^2^*J*_P-H_ coupling constant (measured as 11 Hz, i.e., 10 or 12 Hz), whereas the phospholene region had a large coupling constant. These data are clearly supportive of the structure shown in Figure 4, with the phospholene in the axial position, displaying the H-*trans*-P coupling. The small size of the ^2^*J*_P-H cis_ coupling constant in the phosphite region indicated that a rapidly interconverting mixture of isomers was either not observed or contained a very high ratio of isomers favouring the structure in Figure 4. After 2 h in the pressure vessel, the alkene protons in the phospholene ring were visible in the complex (see Supplementary Materials, HSQC).

Carrying out the reaction of ligand, Rh source and syngas, while stirring for elongated reaction times (7 days) to ensure that whatever change that was implied by the shifting bands in the HPIR spectrum occurred, led to a different compound in comparison with that obtained in the 2 h reaction. There was no extensive fragmentation of the catalyst, but rather a different rhodium–hydride–dicarbonyl complex formed. This complex showed quite different peak shape and shift in the hydride region of the ^1^H-NMR spectrum relative to both [RhH(CO)_2_(**13a**)] and [RhH(CO)_2_(**1**)] (see comparative spectra in Supplementary Materials). The HSQC spectra showed that in the region where the alkene protons of [RhH(CO)_2_(**13a**)] were visible, there was now no resonance. There was no sign of any other alkene protons or multiple overlapping peaks for each signal, nor any change in physical properties, and there was no signal in the aldehyde region of the ^1^H-NMR spectrum. This seemed to rule out polymerisation, hydroformylation, or isomerisation, whilst the fact that the species was not [RhH(CO)_2_(**1**)] ruled out hydrogenation and epimerisation at one of the C-HPh centres. The most likely structure by far was then a simple hydrogenation of the C=C bond, with no other significant changes. One aspect of the NMR spectrum that was not fully explained was that there were twice as many peaks in the spectrum of the catalyst derived from the hydrogenated ligand. More specifically, these were consistent with two very similar isomers formed in a 50/50 ratio, both of which had similar spectral features as those of the single isomer of [RhH(CO)_2_(**13a**)]. Both sets of peaks contained a large ^2^*J*_P-H_ coupling constant for the phospholene ligand, indicating an e–a isomer with phospholene in the apical site. There are two likely explanations for this. One is that [RhH(CO)_2_(**13a**)] and [RhH(CO)_2_(**1**)] had a phosphite unit derived from a *tropos* biphenol that froze to one atropisomer in the complex. This was observed in various forms using *tropos* compounds that either were coordinated to metals or had further chiral centres within their structure. It is possible that the 7 days of heating and ligand hydrogenation led to the hydrogenated ligand product interconverting to both atropisomers, which were both detectable by NMR. Alternatively, it is also possible that the relative stereochemistry of the P-alkyl bond, which was always *syn* to the Ph group for **10**, **13a** and **14**, interconverted by pyramidalization at phosphorous [27,41,42,43,44]. This is a known reaction for a phosphacycle but is normally accomplished at higher temperatures; here, however, the reaction time was very long. The former explanation seems, by far, most likely; in any case, the data are most consistent with a ligand hydrogenation reaction to produce a *meso-cis* phospholane-based ligand. The small shift of the IR bands to lower wavenumbers is consistent with a slight increase in the donor strength of the phosphorous ligand, which is consistent with the basicity of saturated heterocycles versus that of unsaturated heterocycles.

## 3. Conclusions

The observations in this study should be meaningful to various groups of chemists, ranging from those interested in the synthesis and reactivity of phosphocycles to those who study and develop selective hydroformylation catalysts. A new nucleophilic precursor, secondary phospholene borane, was synthesised. This could prove a useful synthon for further studies on phospholene ligands. There is a preference, most likely thermodynamic, in borane precursors (secondary phospholene and its anion) towards one stereoisomer for the final tertiary phospholenes, as confirmed by both NMR and X-ray crystallographic studies.

Whilst outside of the scope of this project, the phospholene C=C bond might provide useful for the further remote functionalisation of a phosphacycle ligand for various purposes and would certainly be of interest in the future. The stereochemical preferences and synthetic routes show that the start of such a project should be relatively straightforward. Phospholene-phosphite ligands have many similarities to the corresponding phospholanes and can readily act as bidentate ligands for Pd and Rh. Activation of H_2_ along with coordination of CO occurred readily to form complexes of the [RhH(CO)_2_(**13**)] type, where the phospholene portion is in the apical site and *trans* to hydride. These complexes were tested in a reaction of industrial interest: the formation of *iso*-butanal with a low *n/iso* ratio (i.e., with some branched selectivity). They appeared very nearly as good as [RhH(CO)_2_(**1**)] in terms of selectivity, reaction rate, or stability with respect to fragmentation/total decomposition. The synthesis of phospholene here described is two-step shorter than that of the analogous phospholanes, including the elimination of one step using a relatively expensive Pd/C catalyst. This is a technical improvement with likely reduced ligand cost, although we note that a catalyst that would clearly justify its incorporation into an established and efficient industrial-scale reaction would desirably be even simpler to access. It was pleasing that a relatively subtle and remote change within a ligand could be detected by in situ HPIR spectroscopic monitoring. The phospholene ligand was cleanly hydrogenated within a few days of operation at 90 °C and 20 bar syngas. This was longer than the operation time of some laboratory-scale batch catalysis reactions, but is relevant to processes that run for a long time. It is probably desirable that phospholene be hydrogenated rather than hydroformylated, since aldehydes are very reactive functional groups, allowing various other reactions to be triggered. In the low-pressure Rh-catalysed hydroformylation of alkyl-alkenes such as cyclopentenes, C=C hydrogenation is rare. Alkene hydrogenation under hydroformylation conditions is only regularly observed with reactive compounds such as unsaturated esters or alkynes. In this case, it seems likely that the C=C bond in the phospholane was able to insert into a Rh-hydride, but due to the coordination of another phosphorous moiety (probably the phosphite), this Rh alkyl was not mobile enough to undergo another migratory insertion reaction with Rh-CO. Instead, hydrogenolysis occurred to produce the saturated ring. In any case, the observation of this possible side reaction could be useful if further studies of phospholenes as ligands in homogeneous catalysis are carried out in the future. Whilst there has now been significant progress towards *iso*-selective hydroformylation reactions, in the case of the simplest but most important example, converting propene to *iso*-butanal, more streamlined ligands would be desirable, as would also be higher *iso*-selectivity.

## 4. Materials and Methods

### 4.1. Safety Note

Hydroformylations make use of hydrogen and carbon monoxide gases. Both are flammable, and CO is toxic. Reactions should only be carried out by trained personnel in pressure vessels designed for high-pressure reactions. The dispensing of CO should be carried out using a controllable cylinder head with a secondary method for stopping the flow of CO. Carbon monoxide detectors should be warned, and adequate signage and control of the laboratory to prevent access from non-trained personnel should be ensured.

### 4.2. General Information

All reactions were performed under an inert atmosphere of nitrogen or argon using standard Schlenk techniques, unless otherwise stated. All glassware used was flame-dried. Dry and degassed solvents were obtained from a solvent still or a solvent purification system (SPS). Commercially purchased anhydrous solvents were degassed before use by the freeze–pump–thaw method or by purging with inert gas. Triethylamine and CDCl_3_ were dried and degassed before use. All chemicals, unless specified, were purchased commercially and used as received. CO/H_2_ and propylene/CO/H_2_ (10/45/45%) were obtained pre-mixed from BOC. NMR spectra were recorded on a Bruker Avance 300, 400 or 500 MHz instrument. Proton chemical shifts are referenced to internal residual solvent protons. Carbon chemical shifts are referenced to the carbon signal of the deuterated solvent. Signal multiplicities are provided as s (singlet), d (doublet), t (triplet), q (quartet), m (multiplet) or a combination of the above. Where appropriate, coupling constants (*J*) are quoted in Hz and are reported to the nearest 0.1 Hz. All spectra were recorded at r.t. (unless otherwise stated), and the solvent for a particular spectrum is indicated in parentheses. NMR spectra of compounds containing phosphorus were recorded under an inert atmosphere in dry and degassed solvent. Gas chromatography was performed on an Agilent Technologies 7820A machine. Mass spectrometry was performed on a Micromass GCT spectrometer, a Micromass LCT spectrometer and on Waters ZQ4000, Thermofisher LTQ Orbitrap XL or Finnigan MAT 900 XLT instruments. Flash column chromatography was performed using Merck Geduran Si 60 (40–63 μm) silica gel. Thin-layer chromatographic (TLC) analyses were carried out using POLYGRAM SIL G/UV254 or POLYGRAM ALOX N/UV254 plastic plates. TLC plates were visualised using a UV visualizer or stained using potassium permanganate dip followed by gentle heating. The synthesis and characterisation of compounds not described below, experimental protocols and spectra can be found in the Supplementary Materials. Crude unprocessed NMR data are available in a data archive [45]. High pressure infrared spectroscopy was performed in a Parr high pressure IR CSTR vessel constructed from Hastelloy C, fitted with CaF_2_ windows and rated to 275 bar. The adjustable pathlength was set to 4 mm. The high-pressure IR spectra were recorded using an Avatar 360 FT-IR. Further discussion of the HPIR set up is available in the Supplementary Materials, and in reference [38]. The presence of ‘unmodified catalysts is ruled out as discussed, in agreement with previous data [38,46].

### 4.3. X-ray Crystallography

X-ray diffraction data for compound **11b** were collected at 125 K using Rigaku MM-007HF high-brilliance RA generator/confocal optics with a XtaLAB P200 diffractometer [Cu Kα radiation (λ = 1.54187 Å)] using Crystal Clear [47]. Intensity data were collected using ω steps, accumulating area detector images spanning at least a hemisphere of reciprocal space. X-ray diffraction data for compounds **13a** and **14** were collected at 125 K using Rigaku FR-X ultrahigh-brilliance Microfocus RA generator/confocal optics with a XtaLAB P200 diffractometer [Mo Kα radiation (λ = 0.71073 Å)] using CrysAlisPro [48]. Data for all compounds analysed were processed (including correction for Lorentz, polarization and absorption) using CrysAlisPro. The structures were solved by dual-space methods (SHELXT [49]) and refined by full-matrix least squares against F^2^ (SHELXL-2019/3) [50]. Non-hydrogen atoms were refined anisotropically, and hydrogen atoms were refined using a riding model, except for the hydrogen atom on O2 in the structure of **11b**, which was located from the difference Fourier map and refined isotropically subject to a distance restraint. The calculations were performed using either the CrystalStructure [51] or the Olex2 [52] interfaces. Selected crystallographic data are presented in Table 3. CCDC 2310520-2310522 contains the supplementary crystallographic data for this paper. These data can be obtained free of charge from the Cambridge Crystallographic Data Centre via www.ccdc.cam.ac.uk/structures.

### 4.4. General Procedure for the Rhodium-Catalysed Hydroformylation of Propene

The hydroformylation reactions of propene were performed in a Parr 4590 Micro Reactor fitted with a gas entrainment stirrer; comprising holes which allowed for better gas dispersion throughout the reaction mixture. The vessel had a volume capacity of 0.1 L, an overhead stirrer with gas entrainment head (set to 1200 r.p.m.), temperature controls, a pressure gauge and the ability to be connected to a gas cylinder. The ligand (10.24 μmol (Rh/L 1:2)) was added to a Schlenk tube, which was then purged with nitrogen (or argon). The internal standard 1-methylnaphthalene (0.1 mL) was then added. The mixture was dissolved in a stock solution of [Rh(acac)(CO)_2_] in toluene (2 mg/mL, 0.65 mL, 5.12 μmol of [Rh(acac)(CO)_2_]), followed by the addition of the designated solvent (19.35 mL). The solution was transferred via a syringe to the pressure vessel (which had been purged with CO/H_2_) through the injection port. CO/H_2_ (1:1) (20 bar) was added, and the heating jacket was set to the desired temperature while stirring. Once the desired temperature was reached, the reaction was stirred for the required time to fully activate the catalyst. Then, pressure was slowly released, and repressurisation was achieved with propene/CO/H_2_. The reaction was then run for the time specified in the tables. After this time, stirring was stopped, and the reaction was cooled by placing the vessel in a basin of cold water. The pressure was released, and the crude sample was analysed immediately by GC (in toluene). The GC method was run on a HP-5 Agilent column with a length of 30 m, a diameter of 0.250 mm and a film of 0.25 μm. The oven was initially held at 25 °C for 6 min, and then the temperature was increased to 60 °C at a rate of 10 °C per minute. The ramp was then increased to 20 °C per minute until the temperature reached 300 °C. The following products with the indicated retention times could be identified: *iso*-butyraldehyde (1.02 min); *n*-butyraldehyde (1.15 min); and 1-methylnaphthalene (13.50 min). The GC was calibrated for propene hydroformylation using (1-methylnaphthalene) as an internal standard. Both the linear (*n*-butyraldehyde) and the branched (*iso*-butyraldehyde) products were calibrated against the internal standard and against each other. Caution: The hydroformylation protocol should be carried out in an adequate vessel for the pressures encountered, and the use of a CO detector is recommended when handling syngas (a poisonous and highly flammable gas).

#### 4.4.1. Synthesis of (*Meso*)-2,5-cis-diphenylphospholene borane Adduct 9: Borane-Protected (*meso*)-2,5-diphenyl-2,5-dihydro-1H-phosphole, **9**

The compound (*meso*)-1-hydroxy-2,5-diphenyl-2,5-dihydrophosphole 1-oxide **(8**) (2.0 g, 7.4 mmol) was suspended in dry and degassed toluene (16 mL) under an inert atmosphere. Phenyl silane (1.83 mL, 14.8 mmol, 2 eq.) was added slowly to the reaction mixture using a syringe. The mixture was then heated to 110 °C and stirred for 17 h. After that time, the reaction mixture was cooled to around 5 °C using an ice bath, and the borane–dimethylsulfide complex (0.783 mL, 8.14 mmol, 1.1 eq.) was added over 1 min. The reaction mixture was then allowed to warm to room temperature and stirred for 4 h. The resulting solution was then filtered through a plug of silica and eluted with toluene (40 mL), and the solvent was removed under reduced pressure to leave a ‘sticky’ colourless solid. The solid was stirred in toluene/heptane 1:4 (10 mL) for 30 min, filtered, washed with toluene/heptane 1:4 (1 × 2 mL) and dried under vacuum to afford the desired product **9** as a white solid (0.654 g, 2.59 mmol, 35%). The organic fractions from the trituration and the washes were concentrated in vacuo. Purification by flash chromatography on silica gel (9:1 hexane/EtOAc) yielded more of the desired product **9** (0.757 g, 3.00 mmol, 40.6%) as a white solid. Combined isolated yield: 1.411 g, 5.59 mmol, 75.6%. ^1^H NMR (CDCl_3_, 500 MHz) δ 7.37–7.26 (10H, m, ArH), 6.25 (2H, d, ^3^*J_P-H_ =* 17.9 Hz, CH=CH), 4.33–4.31 (2H, m, P-CH), 4.29 (1H, dm, ^1^*J_P-H_ =* 367.9 Hz, P-H), 0.84 (3H, q, *J =* 87.5 Hz, BH_3_). ^31^P{^1^H} NMR (CDCl_3_, 202 MHz) δ 57.1 (br d, *J =* 42.8 Hz). ^31^P NMR (CDCl_3_, 202 MHz) δ 57.1 (br dm, ^1^*J_P-H_ =* 367.9 Hz). ^13^C NMR (CDCl_3_, 126 MHz) δ 138.54 (d, *J =* 7.2 Hz 2 × ArC), 133.63 (d, *J =* 2.5 Hz, *C*H=*C*H), 129.38 (d, *J =* 2.0 Hz, 4 × ArCH), 127.74 (d, *J =* 2.4 Hz, 2 × ArCH), 127.35 (d, *J =* 4.2 Hz, 4 × ArCH), 48.66 (d, ^1^*J_C-P_ =* 27.7 Hz, 2 × P-CH). HRMS (ES^+^) C_16_H_18_BPNa [MNa]^+^ m/z: 275.1128 found, 275.1131 required.

#### 4.4.2. Borane-protected 3-((*meso*)-2,5-diphenyl-2,5-dihydro-1H-phosphol-1-yl)-1,1,1-trifluoropropan-2-ol, **10**

To a stirred solution of (*meso*)-2,5-*cis*-diphenylphospholene borane adduct **9** (0.770 g, 3.06 mmol) in THF (13 mL) at −78 °C, under an atmosphere of nitrogen, a 1.58 M solution of *n*-BuLi in hexanes (1.94 mL, 3.06 mmol) was added dropwise via a syringe. The reaction was then allowed to slowly warm to −30 °C, and after stirring for 3 h, a solution of 2-(trifluoromethyl)oxirane (0.29 mL, 3.36 mmol) in THF (4 mL) was added dropwise via a syringe. Once the addition was complete, the reaction was allowed to warm to room temperature and stirred for 2 h. The reaction was quenched by the slow addition of saturated NaHCO_3_ (aq) (5 mL) and water (5 mL) and diluted with diethyl ether (10 mL), and the organic layer was separated. The aqueous layer was extracted with diethyl ether (3 × 10 mL). The organic fractions were combined, dried (MgSO_4_), filtered and concentrated in vacuo to yield a white solid. Purification by flash chromatography on silica gel (3:1 hexane/Et_2_O) yielded the desired product **10** (0.802 g, 2.20 mmol, 72%) as a white solid. ^1^H NMR (CDCl_3_, 500 MHz) δ 7.44–7.24 (10H, m, ArH), 6.32–6.24 (2H, m, CH=CH), 4.63–4.60 (2H, m, P-CH), 3.03–2.96 (1H, m, CH-O), 2.74 (1H, br s, OH), 1.59–0.70 (5H, m, P-CH_2_, BH_3_). ^31^P{^1^H} NMR (CDCl_3_, 202 MHz) δ 46.9 (br d, *J =* 54.7 Hz). ^19^F NMR (CDCl_3_, 470 MHz) δ −80.85 (s). ^13^C NMR (CDCl_3_, 126 MHz) δ 134.46 (d, *J =* 7.6 Hz ArC), 134.33 (d, *J =* 7.4 Hz ArC), 132.87 (d, *J =* 3.2 Hz, *C*H=CH), 132.34 (d, *J =* 3.3 Hz, CH=*C*H), 129.51 (d, *J =* 2.0 Hz, 2 × ArCH), 129.37 (d, *J =* 2.0 Hz, 2 × ArCH), 128.27 (d, *J =* 2.5 Hz, ArCH), 128.16 (d, *J =* 2.5 Hz, ArCH), 127.82 (d, *J =* 3.6 Hz, 2 × ArCH), 127.73 (d, *J =* 3.5 Hz, 2 × ArCH), 123.47 (qd, ^1^*J_C-F_ =* 281.2 Hz, *J =* 16.3 Hz, CF_3_), 65.74 (q, ^2^*J_C-F_ =* 32.7 Hz, OCH), 50.10 (d, ^1^*J_C-P_ =* 28.1 Hz, P-CH), 50.06 (d, ^1^*J_C-P_ =* 27.1 Hz, P-CH), 20.42 (d, ^1^*J_C-P_ =* 28.8 Hz, P-CH_2_). HRMS (ES^+^) C_19_H_21_OBF_3_NaP [MNa]^+^ m/z: 387.1256 found, 387.1267 required.

#### 4.4.3. 4,8-di-tert-butyl-6-((3-((meso)-2,5-diphenyl-2,5-dihydro-1H-phosphol-1-yl)-1,1,1-trifluoropropan-2-yl)oxy)-2,10-dimethoxydibenzo[d,f][1,3,2]dioxaphosphepine, **13a**

3,3′-di-*tert*-butyl-5,5′-dimethoxy-[1,1′-biphenyl]-2,2′-diol [53] (0.271 g, 0.755 mmol) was placed in a Schlenk tube and dissolved in 3 mL of THF. The resulting solution was cooled to −78 °C, and PCl_3_ (0.086 mL, 0.982 mmol) was added slowly. NEt_3_ (0.315 mL, 2.265 mmol) was also added to the reaction mixture, which was then stirred and allowed to reach room temperature over 1 h and then stirred for another hour. The suspension was filtered using a frit under an inert atmosphere, and the filtrate was evaporated using a Schlenk line and dried under vacuum to remove any residual PCl_3_. The crude ^31^P{^1^H} NMR (202.4 MHz, C_6_D_6_) spectrum showed a single peak at δ 172.0 ppm, corresponding to the chlorophosphite. The product was used in the next step without further purification. To a Schlenk flask containing a solution of the chlorophosphite from the previous step in toluene (6 mL) a solution of (*rac*,*meso*)-phospholene **10** (0.250 g, 0.687 mmol) in toluene (6 mL) was added, followed by a solution of 1,4-diazabicyclo-[2,2,2]-octane (DABCO) (0.462 g, 4.12 mmol, 6 eq.) in toluene (5.5 mL). The reaction mixture was then stirred at room temperature overnight (20 h). The resulting suspension was filtered through silica gel (previously dried overnight in an oven) under an inert atmosphere, using dry toluene to compact and wash SiO_2_ after filtration. Purification of (*tropos*,*meso*)-**13a** was achieved by recrystallisation. Heptane (2 mL) was added to a flask containing the reaction mixture; then, the flask was gently warmed with a heat gun, causing the solid to dissolve. The resulting solution was left standing at room temperature, which led to the formation of crystals (0.453 g, 0.615 mmol, 89.5%). ^1^H NMR (C_6_D_6_, 500 MHz) δ 7.17–7.02 (12H, m, ArH), 6.71 (1H, d, *J =* 3.0 Hz, ArH), 6.61 (1H, d, *J =* 3.0 Hz, ArH), 5.85–5.82 (2H, m, CH=CH), 4.39–4.28 (2H, m, P-CH), 3.34 (3H, s, OCH_3_), 3.29 (3H, s, OCH_3_), 3.19–3.08 (1H, m, CH-O), 1.82–1.79 (1H, m, P-CH_2_), 1.47 (9H, s, 3 × CH3), 1.44 (9H, s, 3 × CH3), 1.18–1.12 (1H, m, P-CH_2_). ^31^P{^1^H} NMR (C_6_D_6_, 202 MHz) δ 143.1 (ap dd, *J_P-P_ =* 53.6 Hz, *J_P-F_ =* 3.2 Hz), 5.0 (ap dd, *J_P-P_ =* 53.6 Hz, *J_P-F_ =* 3.2 Hz). ^19^F NMR (C_6_D_6_, 470 MHz) δ −77.73 (ap t, *J_P-F_ =* 4.2 Hz). ^13^C NMR (C_6_D_6_, 126 MHz) δ 156.82 (ArC), 156.00 (ArC), 143.25 (ArC), 143.11 (d, *J =* 11.3 Hz ArC), 142.77 (ArC), 141.21 (ArC), 138.14 (d, *J =* 3.8 Hz ArC), 137.98 (d, *J =* 3.5 Hz ArC), 135.31 (d, *J =* 4.8 Hz ArC), 133.99 (ArC), 135.55 (d, *J =* 3.2 Hz, CH=CH), 134.04 (d, *J =* 3.0 Hz, CH=*C*H), 129.42 (2 × ArCH), 128.91 (2 × ArCH), 128.69 (d, *J =* 1.7 Hz, 2 × ArCH), 128.16 (d, *J =* 1.8 Hz, 2 × ArCH), 127.09 (ArCH), 126.97 (ArCH), 124.46 (qm, ^1^*J_C-F_ =* 281.6 Hz, CF_3_), 115.01 (ArCH), 114.82 (ArCH), 113.65 (ArCH), 112.87 (ArCH), 71.0–69.86 (m, OCH), 55.14 (OCH_3_), 54.08 (OCH_3_), 51.95 (dd, ^1^*J_C-P_ =* 22.2, *J =* 3 Hz, P-CH), 51.68 (d, ^1^*J_C-P_ =* 22.3 Hz, P-CH), 35.67 (*C*(CH_3_)_3_), 35.61 (*C*(CH_3_)_3_), 31.49 (C(*C*H_3_)_3_), 31.24 (d, *J_C-P_ =* 3.7 Hz, C(*C*H_3_)_3_), 21.64 (d, ^1^*J_C-P_ =* 30.6 Hz, P-CH_2_). HRMS (ES^+^) C_41_H_46_O_5_F_3_P_2_ [MH]^+^ m/z: 737.2767 found, 737.2753 required. Recrystallisation from heptane afforded X-ray-quality crystals to determine the relative configuration of the ligand (in racemic form)

The synthesis and characterisation of other ligands and intermediates discussed in this paper can be found in the Supplementary Materials, along with all relevant NMR spectra.

## Figures and Tables

**Table 1:** Comparison of selected bond lengths (Å), angles (º) and torsions (º) for [PdCl_2_(**13a**)]·3CHCl_3_ (**14**) and [PdCl_2_(*S_ax_*,*S*,*S*)-Bobphos·2CHCl_3_ (**NEKXEJ**).

	13a	14	NEKXEJ
Pd-P(1)		2.2023 (5)	2.183 (2)
Pd-P(31)		2.2426 (2)	2.225 (2)
Pd-Cl(2)trans phosphite		2.3385 (5)	2.342 (3)
Pd-Cl(1)trans phosphine		2.3271 (5)	2.348 (2)
P(1)-Pd-P(31)		96.22 (2)	86.13 (9)
Cl(1)-Pd-Cl(2)		92.50 (1)	93.8 (1)
O(2)-P1-O(13)		106.47 (7)	106.5 (4)
C(2)-C(1)-C(12)-C(13)		51.0 (2)	65 (1)
C(6)-C(1)-C(12)-C(17)		45.0 (2)	62 (1)
Phenol-phenol twist	53.05 (4)	50.07 (6)	66.8 (4)

**Table 2:** Hydroformylation of propene catalysed by Rhodium complexes of new phospholane-phosphite ligands.

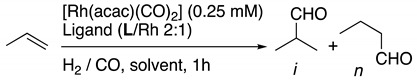				
Entry	Ligand [a]	T [°C]	TON (in 1 h)	*Iso* (%)
1 [b]	**13a**	50	204	71.0
2	**13a**	75	117	67.5
3	**1**	75	121	74.6
4	**13a**	90	333	65.7
5 [c]	**1**	90	397	70.9
6	**13a**	105	751	64.5
7	**1**	105	782	67.2
8	**13b**	75	78	67.0
9	**13b**	90	184	64.8
10	**13b**	105	502	62.3
11	**13c**	75	181	65.3
12	**13c**	90	462	63.3
13	**13c**	105	993	61.0

[a] Catalyst preformed from [Rh(acac)(CO)_2_] (5.12 × 10^−3^ mmol) and ligand (10.24 × 10^−3^ mmol) by stirring at 20 bar of CO/H_2_, at 90 °C for 40 min (**13a**), 45min (**13b**), 30min (**13c**), and 30 min (**1**) in the desired solvent (20 mL) and then increasing or decreasing the reaction T prior to running the reaction using a gas feed of propene/CO/H_2_ at a 1:4.5:4.5 ratio. Rh concentration = 2.52 × 10^−4^ mol dm^−3^. Product determined by GC using 1-methylnaphthalene as an internal standard. The TON values can be treated as average TOF values for the first part of the reaction; see the text for a discussion. [b] Reaction time, 16 h. Average TOF over 16 h = 13. [c] Ligand **1** (major isomer) from reference [17].

**Table 3:** Selected crystallographic data.

	11b	13a	14
formula	C_48_H_50_O_2_	C_41_H_45_O_5_F_3_P_2_	C_44_H_48_O_5_F_3_P_2_Cl_11_Pd
fw	658.92	736.71	1272.11
crystal description	colourless plate	colourless block	yellow prism
crystal size [mm^3^]	0.1 × 0.1 × 0.02	0.12 × 0.11 × 0.04	0.1 × 0.09 × 0.05
space group	*P*3_2_21	*P*2_1_/*c*	*P*2_1_/*n*
*a* [Å]	9.9188 (3)	18.5498 (3)	14.94065 (19)
*b* [Å]		10.34298 (16)	19.3961 (2)
*c* [Å]	32.7080 (13)	20.2110 (3)	18.4869 (2)
*β* [°]		103.8332 (16)	95.1095 (11)
vol [Å]^3^	2786.78 (16)	3765.21 (11)	5336.02 (11)
*Z*	3	4	4
*ρ* (calc) [g/cm^3^]	1.178	1.300	1.583
*μ* [mm^−1^]	0.534	0.174	1.011
F(000)	1062.0	1552.0	2568.0
reflections collected	12302	74481	115446
independent reflections (*R*_int_)	3712 (0.1250)	9058 (0.0333)	13030 (0.0352)
parameters, restraints	235/1	468/27	753/166
GoF on *F*^2^	1.225	1.028	1.031
*R*_1_ [*I* > 2*σ*(*I*)]s	0.0586	0.0386	0.0291
*wR*_2_ (all data)	0.2517	0.0997	0.0682
largest diff. peak/hole [e/Å^3^]	0.44/−0.30	0.30/−0.33	0.55/−0.40

## Data Availability

Crude unprocessed NMR data are available in a data archive [45].

## Supplementary Materials

The following supporting information can be downloaded at: https://www.mdpi.com/article/10.3390/molecules29040845/s1.
