# Supplementary material for: Ligand Hydrogenation during Hydroformylation Catalysis Detected by In Situ High-Pressure Infra-Red Spectroscopic Analysis of a Rhodium/Phospholene-Phosphite Catalyst
Source: Molecules. 2024 Feb 14;29(4):845. doi: 10.3390/molecules29040845 (PMC10891676; doi:10.3390/molecules29040845)
Supplement: Supplementary file 1 [file molecules-29-00845-s001.zip › molecules-2835946-supplementary.pdf]

Supporting Information for:

Ligand Hydrogenation during Hydroformylation Catalysis detected by in-Situ High Pressure Infra-Red Spectroscopic analysis of a Rhodium/ Phospholene-Phosphite catalyst.

José A. Fuentes,[a] Mesfin E. Janka,[b] Aidan McKay,[a] David B. Cordes,[a] Alexandra M. Z. Slawin,[a] Tomas Lebl,[a] Matthew L. Clarke*[a]

[a] Dr. J. A. Fuentes, Dr. A. McKay, Dr. D. Cordes, Prof. A. M. Z. Slawin, Dr. Tomas Lebl, Prof. M. L. Clarke

EaStCHEM School of Chemistry, University of St Andrews,

Purdie Building, North Haugh, St Andrews, KY16 9ST, United Kingdom

E-mail: mc28@st-andrews.ac.uk

[b] Dr Mesfin E. Janka

Eastman Chemical Company, 200 South Wilcox Drive, Kingsport, Tennessee, 37660, USA

Table of Contents

1. General Information.

1.1. High pressure infrared spectroscopy

2. General procedures.

2.1 General Procedure for rhodium-catalysed hydroformylation of propene.

2.2 General procedure for monitoring the complexation of ligands using HPIR spectroscopy.

3. Synthesis of Ligands **13a-c** precursors.

3.1 Synthesis of borane protected phospholene adduct **9**.

3.2. Synthesis of borane protected phospholene adduct **10**.

3.3. Synthesis of 3,3',5,5'-tetrakis(2-phenylpropan-2-yl)-[1,1'-biphenyl]-2,2'-diol, **11b**.

3.4. Synthesis of 3,3'-di-*tert*-butyl-5,5'-dichloro-[1,1'-biphenyl]-2,2'-diol, **11c**.

4. Synthesis of phospholene-phosphite ligands **13a-13c**.

5. Preparation of [$PdCl_2$**13a**], **14**.

6. 1D gs-NOESY Spectra of **5** and **9**

7. Coordination and stability studies.

7.1 NMR spectroscopic investigation of the coordination mode of [RhH$(CO)_2$(*tropos*,*meso*)-**13a**)].

7.2 NMR spectroscopic investigation of the stability under syngas at 90 °C of [RhH$(CO)_2$(*tropos*,*meso*)-**13a**)]. One week-long experiment.

8. HPIR spectroscopic activation study of phospholane-phosphites **13a-13c** with [Rh(acac)$(CO)_2$].

9. HPIR spectroscopic thermal stability study of phospholane-phosphites **13a-13c** with [Rh(acac)$(CO)_2$].

10. NMRs of selected compounds

11. References.

1. General Information.

All reactions were performed under an inert atmosphere of nitrogen or argon using standard Schlenk techniques, unless otherwise stated. All glassware used was flame-dried. Dry and degassed solvents were obtained from a solvent still or SPS solvent purification system.

Commercially purchased anhydrous solvents were degassed before use by the freeze-pump-thaw method or by purging with inert gas. Triethylamine and $CDCl_3$ were dried and degassed before use. All chemicals, unless specified were purchased commercially and used as received. CO/H_2 and propylene/CO/H_2 (10/45/45%) were obtained pre-mixed from BOC.

NMR spectra were recorded on a Bruker Avance 300, 400 or 500 MHz instrument. Proton chemical shifts are referenced to internal residual solvent protons. Carbon chemical shifts are referenced to the carbon signal of the deuterated solvent. Signal multiplicities are given as s (singlet), d (doublet), t (triplet), q (quartet), m (multiplet) or a combination of the above. Where appropriate coupling constants (*J*) are quoted in Hz and are reported to the nearest 0.1 Hz. All spectra were recorded at r.t. (unless otherwise stated) and the solvent for a particular spectrum is given in parentheses. NMR of compounds containing phosphorus were recorded under an inert atmosphere in dry and degassed solvent.

Gas chromatography was performed on an Agilent Technologies 7820A machine.

Mass spectrometry was performed on a Micromass GCT spectrometer, Micromass LCT spectrometer, Waters ZQ4000, Thermofisher LTQ Orbitrap XL or Finnigan MAT 900 XLT instruments.

Flash column chromatography was performed using Merck Geduran Si 60 (40-63 μm) silica gel.

Thin layer chromatographic (TLC) analyses were carried out using POLYGRAM SIL G/UV254 or POLYGRAM ALOX N/UV254 plastic plates. TLC plates were visualised using a UV visualizer or stained using potassium permanganate dip followed by gentle heating.

3,3'-di-tert-butyl-5,5'-dimethoxy-[1,1'-biphenyl]-2,2'-diol[53] and (*meso*)-1-hydroxy-2,5-diphenyl-2,5-dihydrophosphole 1-oxide[25], **8,** were prepared via the synthetic routes reported previously.

1.1. High pressure infrared spectroscopy

High pressure infrared spectroscopy was performed in a Parr high pressure IR CSTR vessel constructed from Hastelloy C, fitted with CaF_2 windows and rated to 275 bar. The adjustable pathlength was set to 4 mm. The high pressure IR spectra were recorded using an Avatar 360 FT-IR.

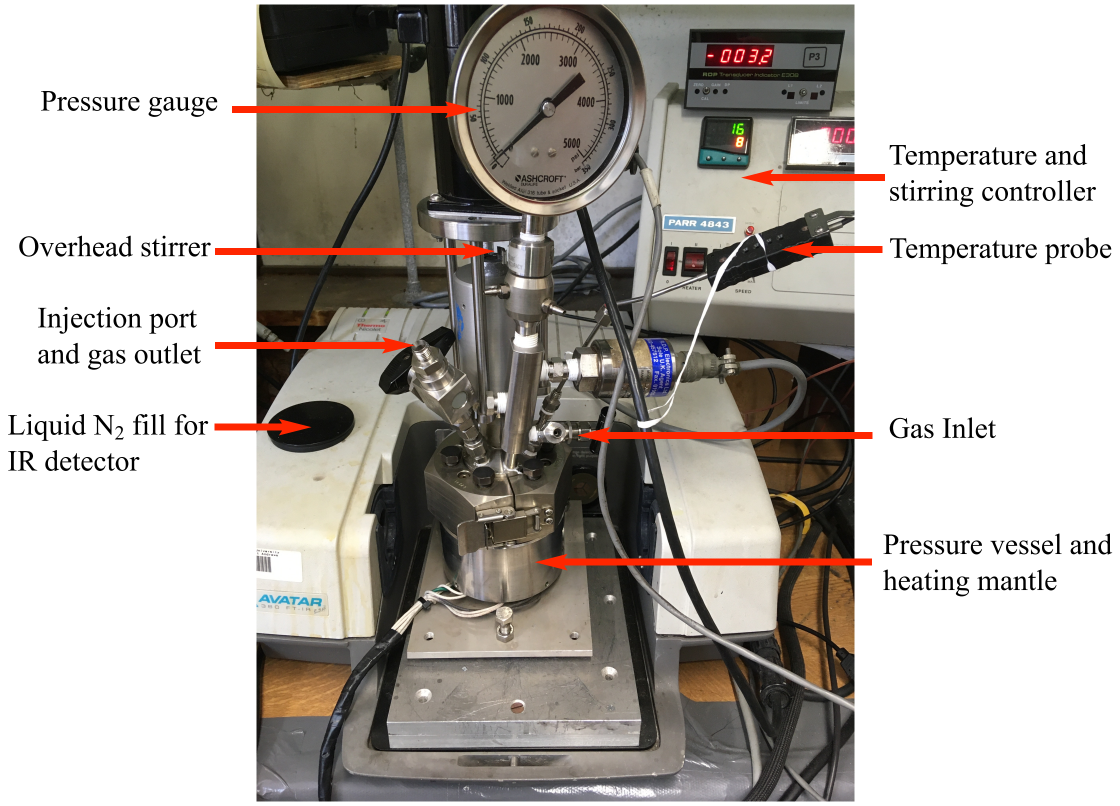

Figure S.1.1. High pressure infrared spectroscopy apparatus

In reference[38], we described further aspects of validating the HPIR set up. Catalyst decomposition of a phosphine modified Rh catalyst can take many forms, some of them giving inactive Rh catalysts such as Rh metal or certain dimeric species with bridging phosphido ligands. However, it is common for the decomposed catalysts to also be some form of hydroformylation catalyst, either with an altered phosphine ligand (such as a monodentate ligand formed after cleavage of a bidentate ligand into two or more components). Another possibility is that after ligand decoordination and decomposition, the so-called unmodified Rh catalysts can be present. In reference [3], HPIR spectra of these catalysts in this HPIR set up are presented in the ESI. These show that using [Rh_2(hexanoate)$_4$] as precursor, the unmodified catalyst takes a cluster resting state with peaks at 2074, 2043 and 1822 cm^{-1}. This is consistent with previous work[46] assigning this species as [$Rh_6(CO)_{16}$]. This is one resting state for the unmodified catalyst under hydroformylation conditions. [Rh(acac)$(CO)_2$] the precursor used in these experiments, tends to remain partially intact under hydroformylation conditions (IR bands at 2082 and 2015 cm^{-1}), but also accompanied by two clusters [2074, 2069, 2044 and 1888, which are assigned to [$Rh_4(CO)_{12}$)] and lower levels of [$Rh_6(CO)_{16}$] noted above.

In the stability studies of the ligands presented in this paper we did not find any evidence of the presence of [Rh(acac)$(CO)_2$], [$Rh_4(CO)_{12}$)] or [$Rh_6(CO)_{16}$] in noticeable amounts (any band in the 1888-1822 cm^{-1} region would be especially easily spotted since this is an uncluttered area in the spectra).

2. General procedures.

2.1 General Procedure for rhodium-catalysed hydroformylation of propene.

Hydroformylation reactions of propene were performed in a Parr 4590 Micro Reactor fitted with a gas entrainment stirrer; comprising of holes which gives better gas dispersion throughout the reaction mixture. The vessel had a volume capacity of 0.1 L, an overhead stirrer with gas entrainment head (set to 1200 r.p.m.), temperature controls, pressure gauge and the ability to be connected to a gas cylinder.

Ligand (10.24 µmol (Rh:L 1:2)) was added to a Schlenk tube, which was then purged with nitrogen (or argon). The internal standard 1-methylnaphthalene (0.1 mL) was then added. The mixture was dissolved in a stock solution of [Rh(acac)$(CO)_2$] in toluene (2 mg/mL, 0.65 mL, 5.12 µmol of [Rh(acac)$(CO)_2$]), followed by the addition of the designated solvent (19.35 mL). The solution was transferred *via* syringe to the pressure vessel (which had been purged with CO/H_2) through the injection port. CO/H_2(1:1) (20 bar) was added and the heating jacket set to the desired temperature while stirring. Once the desired temperature was reached, the reaction was stirred for the required time to fully activate the catalyst. Then pressure was slowly released and repressurised with propene/CO/H_2. The reaction was then run for the time specified in the tables. After this time, stirring was stopped and the reaction was cooled by placing the vessel in a basin of cold water. The pressure was released, and the crude sample was analysed immediately by GC (in C_7H_8). The GC method was run on a HP-5 Agilent column; with length 30 m, diameter 0.250 mm and film 0.25 µm. The oven was initially held at 25 °C for 6 minutes, and then increased to 60 °C at a rate of 10 °C per minute. The ramp was then increased to 20 °C per minute until the temperature reached 300 °C. The products could be identified with the following retention times; *iso*-butyraldehyde (1.02 min); *n*-butyraldehyde (1.15 min) and 1-methylnaphthalene (13.50 min). The GC was calibrated for propene hydroformylation using (1-methylnaphthalene) as an internal standard. Both the linear (*n*-butyraldehyde) and branched (*iso*-butyraldehyde) products were calibrated against the internal standard and against each other.

2.2 General procedure for monitoring the complexation of ligands using HPIR spectroscopy.

The HPIR spectroscopy pressure vessel was purged with CO/H_2 three times and then n-dodecane (22 mL) was injected into the pressure vessel. The heating jacket set to 90 °C (or the desired temperature) while stirring. Once the required temperature was reached, the vessel was pressurised at 20 bar of syngas and a background spectrum was recorded (1024 scans). [Rh(acac)$(CO)_2$] (0.06 mmol) and ligand (0.075 mmol (Rh:L 1:1.25)) was added to a flame dried schlenk tube, which was then purged with nitrogen (or argon). n-dodecane (8 mL) was added to dissolve the rhodium precursor and ligand and stirred for 10 minutes. The reaction mixture was then transferred *via* syringe to the pressure vessel (which had been depresurized before

addition) through the injection port, CO/H_2 (20 bar) was added and sampling initiated, 128 scans per spectrum. Active catalyst formation was then monitored by IR.

3. Synthesis of Ligands 13a-c precursors.

3.1 Synthesis of borane protected phospholene adduct 9.

Synthesis of (*meso*)-2,5-*cis*-diphenylphospholene-borane adduct 9: Borane protected-(*meso*)-2,5-diphenyl-2,5-dihydro-1*H*-phosphole, 9.

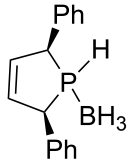

(*meso*)-1-Hydroxy-2,5-diphenyl-2,5-dihydrophosphole 1-oxide **(8)** (2.0 g, 7.4 mmol) was suspended in dry and degassed toluene (16 mL) under an inert atmosphere. To the reaction mixture was added phenyl silane (1.83 mL, 14.8 mmol, 2 eq.) slowly *via* syringe. The mixture was then heated to 110 °C and stirred for 17h. After that time, the reaction mixture was cooled to around 5 °C using an ice bath and borane-dimethylsulfide complex (0.783 mL, 8.14 mmol, 1.1 eq.) added over 1 min. The reaction mixture was then allowed to warm to room temperature and stirred for 4h. The resulting solution was then filtered through a plug of silica, eluted with toluene (40 mL) and the solvent removed under reduced pressure to leave a 'sticky' colourless solid. The solid was stirred in toluene:heptane 1:4 (10 mL) for 30 min, filtered, washed with toluene: heptane 1:4 (1 x 2 mL) and dried under vacuum to afford the desired product **9** as a white solid (0.654 g, 2.59 mmol, 35%). The organic fractions from the trituration and the washes were concentrated in *vacuo*. Purification by flash chromatography on silica gel (9:1 hexane:EtOAc) yielded more of the desired product **9** (0.757 g, 3.00 mmol, 40.6%) as a white solid. Combined isolated yield (1.411 g, 5.59 mmol, 75.6%). ^{1}H NMR ($CDCl_3$, 500 MHz) δ 7.37-7.26 (10H, m, ArH), 6.25 (2H, d, $^3J_{P-H}$ = 17.9 Hz, CH=CH), 4.33-4.31 (2H, m, P-CH), 4.29 (1H, dm, $^1J_{P-H}$ = 367.9 Hz, P-H), 0.84 (3H, q, J = 87.5 Hz, BH_3). ^{31}P{^{1}H} NMR ($CDCl_3$, 202 MHz) δ 57.1 (br d, J = 42.8 Hz). ^{31}P NMR ($CDCl_3$, 202 MHz) δ 57.1 (br dm, $^1J_{P-H}$ = 367.9 Hz). ^{13}C NMR ($CDCl_3$, 126 MHz) δ 138.54 (d, J = 7.2 Hz 2 x ArC), 133.63 (d, J = 2.5 Hz, *C*H=*C*H), 129.38 (d, J = 2.0 Hz, 4 x ArCH), 127.74 (d, J = 2.4 Hz, 2 x ArCH), 127.35 (d, J = 4.2 Hz, 4 x ArCH), 48.66 (d, $^1J_{C-P}$ = 27.7 Hz, 2 x P-CH). HRMS (ES$^+$) $C_{16}H_{18}BPNa$ [MNa]$^+$ m/z: 275.1128 found, 275.1131 required.

3.2. Synthesis of borane protected phospholene adduct 10.

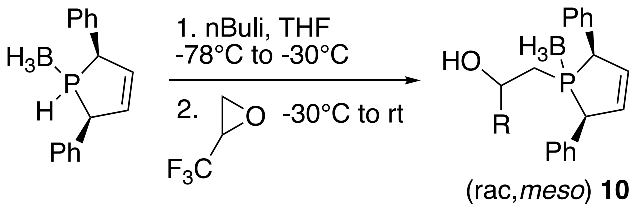

Scheme S.3.2. Preparation of precursors for ligands **13a**, **13b**, and **13c**

Synthesis of borane protected phospholene adduct 10: Borane-protected-3-((*meso*)-2,5-diphenyl-2,5-dihydro-1*H*-phosphol-1-yl)-1,1,1-trifluoropropan-2-ol

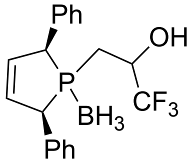

To a stirred solution of (*meso*)-2,5-*cis*-diphenylphospholene-borane adduct **9** (0.770 g, 3.06 mmol) in THF (13 mL) at –78°C, under an atmosphere of nitrogen, was added a 1.58 M solution of *n*-BuLi in hexanes (1.94 mL, 3.06 mmol) drop-wise *via* syringe. The reaction was then allowed to slowly warm to -30°C and after stirring for 3 h a solution of 2-(trifluoromethyl)oxirane (0.29 ml, 3.36 mmol) in THF (4 mL) was added drop-wise *via* syringe. Once the addition was complete the reaction was allowed to warm to room temperature and stirred for 2 h. The reaction was quenched by slow addition of saturated $NaHCO_3$ (aq) (5 mL) and water (5 mL), diluted with diethyl ether (10 mL) and the organic layer separated. The aqueous layer was extracted with diethyl ether (3 x 10 mL). The organic fractions were combined, dried ($MgSO_4$), filtered and concentrated in *vacuo* to give a white solid. Purification by flash chromatography on silica gel (3:1 hexane:Et_2O) yielded the desired product **10** (0.802 g, 2.20 mmol, 72%) as a white solid. ^{1}H NMR ($CDCl_3$, 500 MHz) δ 7.44-7.24 (10H, m, ArH), 6.32-6.24 (2H, m, CH=CH), 4.63-4.60 (2H, m, P-CH), 3.03-2.96 (1H, m, CH-O), 2.74 (1H, br s, OH), 1.59-0.70 (5H, m, P-CH_2, BH_3). ^{31}P{^{1}H} NMR ($CDCl_3$, 202 MHz) δ 46.9 (br d, *J* = 54.7 Hz). ^{19}F NMR ($CDCl_3$, 470 MHz) δ –80.85 (s). ^{13}C NMR ($CDCl_3$, 126 MHz) δ 134.46 (d, *J* = 7.6 Hz ArC), 134.33 (d, *J* = 7.4 Hz ArC), 132.87 (d, *J* = 3.2 Hz, *C*H=CH), 132.34 (d, *J* = 3.3 Hz, CH=*C*H), 129.51 (d, *J* = 2.0 Hz, 2 x ArCH), 129.37 (d, *J* = 2.0 Hz, 2 x ArCH), 128.27 (d, *J* = 2.5 Hz, ArCH), 128.16 (d, *J* = 2.5 Hz, ArCH), 127.82 (d, *J* = 3.6 Hz, 2 x ArCH), 127.73 (d, *J* = 3.5 Hz, 2 x ArCH), 123.47 (qd, $^1J_{C\text{-}F}$ = 281.2 Hz, *J* = 16.3 Hz, CF_3), 65.74 (q, $^2J_{C\text{-}F}$ = 32.7 Hz, OCH), 50.10 (d, $^1J_{C\text{-}P}$ = 28.1 Hz, P-CH), 50.06 (d, $^1J_{C\text{-}P}$ = 27.1 Hz, P-CH), 20.42 (d, $^1J_{C\text{-}P}$ = 28.8 Hz, P-CH_2). HRMS (ES^+) $C_{19}H_{21}OBF_3NaP$ $[MNa]^+$ m/z: 387.1256 found, 387.1267 required.

3.3. Synthesis of 3,3',5,5'-tetrakis(2-phenylpropan-2-yl)-[1,1'-biphenyl]-2,2'-diol, 11b.

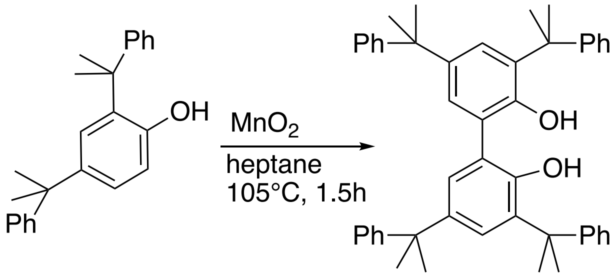

11b

2,4-Bis-(α,α-dimethylbenzyl)phenol (16.52 g, 50.0 mmol) was dissolved in heptane (85 mL). To this was added manganese(IV) oxide activated (6.6 g, 64.5 mmol) and the mixture was heated at 105°C for 1.5 hours. The reaction mixture was then allowed to cool down to room temperature, filtered through celite and the filtrate was concentrated *in vacuo*. The crude product was purified by trituration with hexane (40 mL) to afford the desired product **11b** (9.336 g, 14.17 mmol, 56.7%) as a pale yellow solid. ^{1}H NMR ($CDCl_3$, 500 MHz) δ 7.27-7.13 (22H, m, ArH), 6.93-6.92 (2H, m, ArH), 5.06 (2H, s, OH), 1.66 (12H, s, 4 x CH_3), 1.58 (12H, s, 4 x CH_3). ^{13}C NMR ($CDCl_3$, 126 MHz) δ 150.82 (2 x ArC), 149.88 (2 x ArC), 148.75 (2 x ArC), 142.64 (2 x ArC), 135.64 (2 x ArC), 125.75 (2 x ArC), 128.53-125.67 (24 x ArCH), 42.79 (2 x *C*(CH_3)$_2$Ph), 42.33 (2 x *C*(CH_3)$_2$Ph), 31.15 (8 x CH_3). HRMS (ES$^+$) $C_{48}H_{50}O_2Na$ [MNa]$^+$ m/z: 681.3709 found, 681.3703 required. Recrystallisation from hexane afforded X-Ray quality crystals to determine the structure of the compound (Figure **S.3.1**).

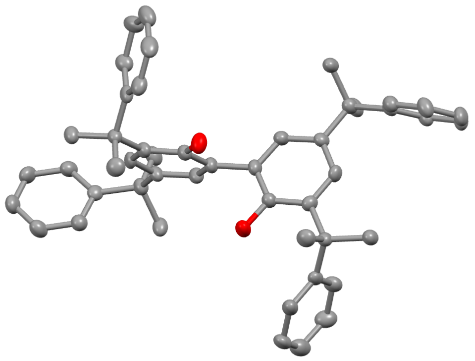

Figure S.3.1. View of an independent molecule in the structure of **11b**. Hydrogen atoms omitted for clarity.

3.4. Synthesis of 3,3'-di-*tert*-butyl-5,5'-dichloro-[1,1'-biphenyl]-2,2'-diol, 11c.

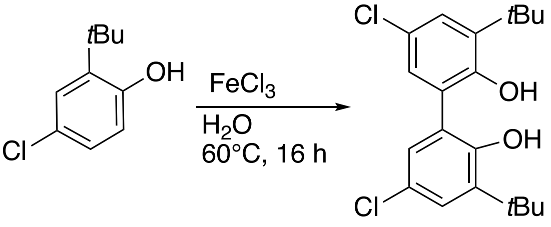

11c

2-(*tert*-butyl)-4-chlorophenol (277 mg, 1.5 mmol), $FeCl_3$ (1.0 g, 6 mmol) and water (12 mL) were placed in a flask and the mixture was heated at 60°C for 16 hours. The reaction mixture was then allowed to cool down to room temperature and extracted with dichloromethane (12 ml x 3). The organic fractions were combined, dried ($MgSO_4$), filtered and concentrated in *vacuo* to give the crude product. Purification by flash chromatography on silica gel (50:1 hexane:Et_2O) yielded the desired product **11c** (88 mg, 0.24 mmol, 16 %) as a pale orange solid. ^{1}H NMR ($CDCl_3$, 400 MHz) δ 7.35 (2H, d, *J* = 2.6 Hz, ArH), 7.09 (2H, d, *J* = 2.6 Hz, ArH), 5.21 (2H, s, OH), 1.44 (18H, s, 2 x CH_3). ^{13}C NMR ($CDCl_3$, 101 MHz) δ 150.78 (2 x ArC), 139.66 (2 x ArC), 128.63 (2 x ArCH), 128.10 (2 x ArCH), 125.85 (2 x ArC), 123.08 (2 x ArC), 35.43 (2 x *C*(CH_3)$_3$), 29.47 (2 x CH_3). HRMS (ES^-) $C_{20}H_{23}O_2Cl_2$ $[M-H]^-$ m/z: 365.1081 found, 365.1082.

4. Synthesis of phospholene-phosphite ligands 13a-13c.

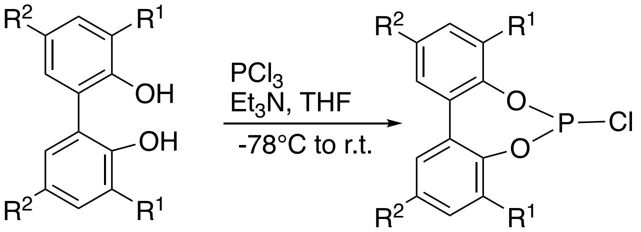

R¹= *t*Bu, R²= MeO,**11a**
R¹= PhMe$_2$C, R²= PhMe$_2$C, **11b**
R¹= *t*Bu, R²= Cl, **11c**

From **11a**: **12a**
From **11b**: **12b**
From **11c**: **12c**

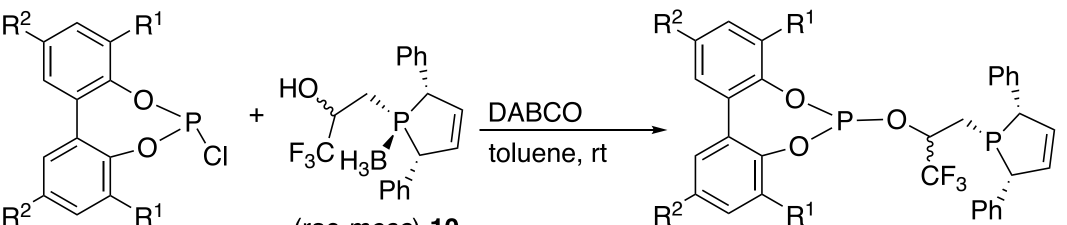

12a-c

(rac,*meso*)**-10**

R¹= *t*Bu, R²= MeO,**13a**
R¹= PhMe$_2$C, R²= PhMe$_2$C, **13b**
R¹= *t*Bu, R²= Cl, **13c**

Scheme S.4.1. Preparation of ligands **13a, 13b, and 13c**

4,8-di-*tert*-butyl-6-((3-((*meso*)-2,5-diphenyl-2,5-dihydro-1*H*-phosphol-1-yl)-1,1,1-trifluoropropan-2-yl)oxy)-2,10-dimethoxydibenzo[*d,f*][1,3,2]dioxaphosphepine, 13a.

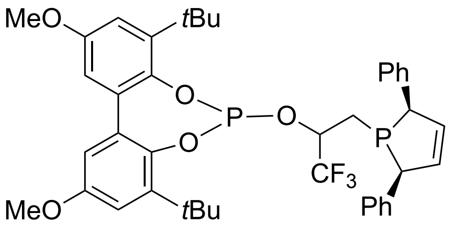

3,3'-di-*tert*-butyl-5,5'-dimethoxy-[1,1'-biphenyl]-2,2'-diol (0.271 g, 0.755 mmol) was placed in a Schlenk tube and dissolved in 3 mL of THF. The resulting solution was cooled to –78 ºC and PCl_3 (0.086 mL, 0.982 mmol) was added slowly. NEt_3 (0.315 mL, 2.265 mmol) was also added to the reaction mixture, which was then stirred and allowed to reach room temperature over 1 h and then stirred for another hour. The suspension was filtered using a frit under an inert atmosphere, and the filtrate was evaporated using a Schlenk line and dried under vacuum to remove any residual PCl_3. The crude $^{31}P\{^1H\}$ NMR (202.4 MHz, C_6D_6) spectrum showed a single peak at δ 172.0 ppm, corresponding to the chlorophosphite. The product was used in the next step without further purification. To a Schlenk flask containing a solution of the chlorophosphite from the previous step in toluene (6 mL) was added a solution of (*rac*,*meso*)-phospholene **10** (0.250 g, 0.687 mmol) in toluene (6 mL) followed by a solution of 1,4-diazabicyclo-[2,2,2]-octane (DABCO) (0.462 g, 4.12 mmol, 6 eq.) in toluene (5.5 mL). The reaction mixture was then allowed to stir at room temperature overnight (20 h). The resulting suspension was filtered through silica gel (previously dried overnight in an oven) under an inert atmosphere, using dry toluene to compact and wash the SiO_2 after filtration. Purification of (*tropos*,*meso*)-**13a** was achieved by recrystallisation. Heptane (2 mL) was added to a flask containing the reaction mixture then the flask was gently warmed with a heat gun causing the solid to dissolve. The resulting solution was left standing at room temperature which led to the formation of crystals (0.453 g, 0.615 mmol, 89.5 %). ^1H NMR (C_6D_6, 500 MHz) δ 7.17-7.02 (12H, m, ArH), 6.71 (1H, d, J = 3.0 Hz, ArH), 6.61 (1H, d, J = 3.0 Hz, ArH), 5.85-5.82 (2H, m, CH=CH), 4.39-4.28 (2H, m, P-CH), 3.34 (3H, s, OCH_3), 3.29 (3H, s, OCH_3), 3.19-3.08 (1H, m, CH-O), 1.82-1.79 (1H, m, P-CH_2), 1.47 (9H, s, 3 x CH_3), 1.44 (9H, s, 3 x CH_3), 1.18-1.12 (1H, m, P-CH_2). $^{31}P\{^1H\}$ NMR (C_6D_6, 202 MHz) δ 143.1 (ap dd, $J_{P\text{-}P}$ = 53.6 Hz, $J_{P\text{-}F}$ = 3.2 Hz), 5.0 (ap dd, $J_{P\text{-}P}$ = 53.6 Hz, $J_{P\text{-}F}$ = 3.2 Hz). ^{19}F NMR (C_6D_6, 470 MHz) δ –77.73 (ap t, $J_{P\text{-}F}$ = 4.2 Hz). ^{13}C NMR (C_6D_6, 126 MHz) δ 156.82 (ArC), 156.00 (ArC), 143.25 (ArC), 143.11 (d, J = 11.3 Hz ArC), 142.77 (ArC), 141.21 (ArC), 138.14 (d, J = 3.8 Hz ArC), 137.98 (d, J = 3.5 Hz ArC), 135.31 (d, J = 4.8 Hz ArC), 133.99 (ArC), 135.55 (d, J = 3.2 Hz, *C*H=CH), 134.04 (d, J = 3.0 Hz, CH=*C*H), 129.42 (2 x ArCH), 128.91 (2 x ArCH), 128.69 (d, J = 1.7 Hz, 2 x ArCH), 128.16 (d, J = 1.8 Hz, 2 x ArCH), 127.09 (ArCH), 126.97 (ArCH), 124.46 (qm, $^1J_{C\text{-}F}$ = 281.6 Hz, CF_3), 115.01 (ArCH), 114.82 (ArCH), 113.65 (ArCH), 112.87 (ArCH), 71.0-69.86 (m, OCH), 55.14 (OCH_3), 54.08 (OCH_3), 51.95 (dd, $^1J_{C\text{-}P}$ = 22.2, J = 3 Hz, P-CH), 51.68 (d, $^1J_{C\text{-}P}$ = 22.3 Hz, P-CH), 35.67 (*C*(CH_3)$_3$), 35.61 (*C*(CH_3)$_3$), 31.49 (C(*C*H_3)$_3$), 31.24 (d, $J_{C\text{-}P}$ = 3.7 Hz, C(*C*H_3)$_3$), 21.64 (d, $^1J_{C\text{-}P}$ = 30.6 Hz, P-CH_2). HRMS (ES^+) $C_{41}H_{46}O_5F_3P_2$ $[MH]^+$ m/z: 737.2767 found, 737.2753 required. Recrystallisation from heptane

afforded X-Ray quality crystals to determine the absolute configuration of the ligand (in racemic form) (**Figure S4.1**).

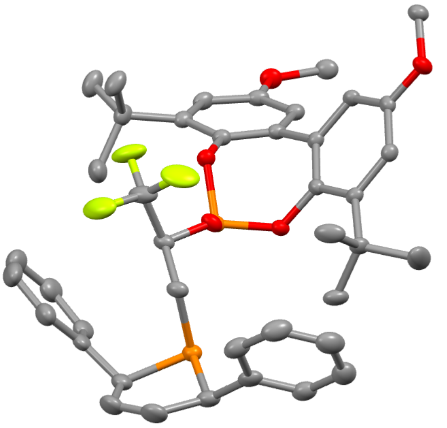

Figure S.4.1. View of an independent molecule in the structure of **13a**. Hydrogen atoms omitted for clarity.

6-((3-((*meso*)-2,5-diphenyl-2,5-dihydro-1*H*-phosphol-1-yl)-1,1,1-trifluoropropan-2-yl)oxy)-2,4,8,10-tetrakis(2-phenylpropan-2-yl)dibenzo[*d,f*][1,3,2]dioxaphosphepine, 13b.

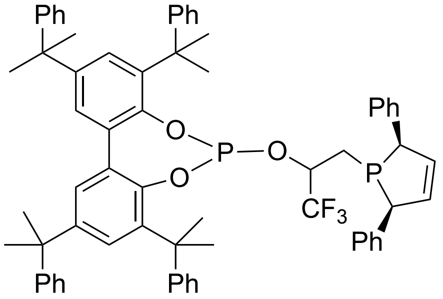

3,3',5,5'-Tetrakis(2-phenylpropan-2-yl)-[1,1'-biphenyl]-2,2'-diol (0.738 g, 1.12 mmol) was placed in a Schlenk tube and dissolved in 6 mL of THF. The resulting solution was cooled to –78 ºC and PCl_3 (0.172 mL, 1.97 mmol) was added slowly. NEt_3 (0.468 mL, 3.36 mmol) was also added to the reaction mixture, which was then stirred and allowed to reach room temperature over 1 h and then stirred for another hour. The suspension was filtered using a frit under an inert atmosphere, and the filtrate was evaporated using a Schlenk line and dried under vacuum to remove any residual PCl_3. The crude $^{31}P\{^1H\}$ NMR (202.4 MHz, C_6D_6) spectrum showed a single peak at δ 172.8 ppm, corresponding to the chlorophosphite. The product was used in the next step without further purification. To a Schlenk flask containing a solution of the chlorophosphite from the previous step in toluene (7 mL) was added a solution of (*rac,meso*)-phospholane **10** (0.328 g, 0.9 mmol) in toluene (7 mL) followed by a solution of 1,4-diazabicyclo-[2,2,2]-octane (DABCO) (0.706 g, 6.3 mmol, 7 eq.) in toluene (5 mL). The reaction mixture was then allowed to stir at

room temperature overnight (18 h). The resulting suspension was filtered through silica gel (previously dried overnight in an oven) under an inert atmosphere, using dry toluene to compact and wash the SiO_2 after filtration. Purification of (*tropos*,*meso*)-**13b** was achieved by flash chromatography on silica gel (previously deactivated by eluting Et_3N/hexane 0.25% solution before running the column) (20:1 hexane:EtOAc) yielded the desired product **13b** (0.511 g, 0.49 mmol, 55%) as a pale yellow solid. ^{1}H NMR (C_6D_6, 500 MHz) δ 7.34-6.91 (34H, m, ArH), 5.91-5.82 (2H, m, CH=CH), 4.39-4.30 (2H, m, P-CH), 3.51-3.37 (1H, m, CH-O), 1.97 (3H, s, CH_3), 1.64 (3H, s, CH_3), 1.60 (6H, s, 2 x CH_3), 1.60-1.57 (1H, m, P-CH_2), 1.57 (3H, s, CH_3), 1.57 (3H, s, CH_3), 1.54 (3H, s, CH_3), 1.51 (3H, s, CH_3), 1.16-1.11 (1H, m, P-CH_2). ^{31}P{^{1}H} NMR (C_6D_6), 202 MHz δ 148.64-148.47 (br m), 8.64 (br s). ^{19}F NMR (C_6D_6, 470 MHz) δ –77.14 - –77.20 (m). ^{13}C NMR (C_6D_6, 126 MHz) δ 151.47 (ArC), 151.27 (ArC), 150.57 (ArC), 150.38 (ArC), 146.70 (ArC), 146.16 (ArC), 141.31 (ArC), 141.13 (ArC), 138.45 (d, J = 3.2 Hz ArC), 138.35 (d, J = 3.5 Hz ArC), 133.41 (ArC), 132.89 (ArC), 134.87 (d, J = 2.8 Hz, *C*H=CH), 134.28 (d, J = 2.1 Hz, CH=*C*H), 129.31-125.37 (34 x ArCH), 124.29 (qm, $^1J_{C-F}$ = 285.215 Hz, CF_3), 71.55-70.51 (m, OCH), 51.82 (br d, $^1J_{C-P}$ = 21.8Hz, 2 x P-CH), 43.45 (*C*(CH_3)$_2$Ph), 43.32 (*C*(CH_3)$_2$Ph), 43.00 (*C*(CH_3)$_2$Ph), 42.91 (*C*(CH_3)$_2$Ph), 33.21 (CH_3), 32.49 (CH_3), 30.88 (2 x CH_3), 30.84 (CH_3), 30.75 (CH_3), 29.37 (CH_3), 28.72 (CH_3), 20.22 (d, $^1J_{C-P}$ = 29.1 Hz, P-CH_2). HRMS (ES$^+$) $C_{67}H_{66}O_3F_3P_2$ [MH]$^+$ m/z: 1037.4427 found, 1037.4434 required.

4,8-di-*tert*-butyl-2,10-dichloro-6-((3-((2*R*,5*S*)-2,5-diphenyl-2,5-dihydro-1*H*-phosphol-1-yl)-1,1,1-trifluoropropan-2-yl)oxy)dibenzo[*d*,*f*][1,3,2]dioxaphosphepine, 13c.

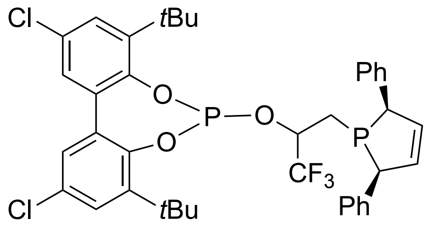

3,3'-di-*tert*-butyl-5,5'-dichloro-[1,1'-biphenyl]-2,2'-diol, **11c** (0.239 g, 0.65 mmol) was placed in a Schlenk tube and dissolved in 3 mL of THF. The resulting solution was cooled to –78 ºC and PCl_3 (0.1 mL, 1.146 mmol) was added slowly. NEt_3 (0.27 mL, 1.95 mmol) was also added to the reaction mixture, which was then stirred and allowed to reach room temperature over 1 h and then stirred for another hour. The suspension was filtered using a frit under an inert atmosphere, and the filtrate was evaporated using a Schlenk line and dried under vacuum to remove any residual PCl_3. The crude ^{31}P{^{1}H} NMR (202.4 MHz, C_6D_6) spectrum showed the peak corresponding to the chlorophosphite at δ 176.0 ppm. The product was used in the next step without further purification. To a Schlenk flask containing a solution of the chlorophosphite from the previous step in toluene (4 mL) was added a solution of (*rac*,*meso*)-phospholene **10** (0.190 g, 0.52 mmol) in toluene (4 mL) followed by a solution of 1,4-diazabicyclo-[2,2,2]-octane (DABCO) (0.400 g, 3.57 mmol, 6.9 eq.) in toluene (4 mL). The reaction mixture was then allowed to stir at room temperature overnight (20 h). The resulting suspension was filtered through silica gel (previously

dried overnight in an oven) under an inert atmosphere, using dry toluene to compact and wash the SiO_2 after filtration. Purification of (*tropos*,*meso*)-**13c** was achieved by flash chromatography on silica gel (previously deactivated by eluting Et_3N/hexane 0.25% solution before running the column) (20:1 hexane:EtOAc) yielded the desired product **13c** (0.310 g, 0.42 mmol, 80%) as a pale orange solid. ^{1}H NMR (C_6D_6, 500 MHz) δ 7.41 (1H, d, J = 2.6 Hz, ArH), 7.35 (1H, d, J = 2.6 Hz, ArH), 7.16-6.97 (10H, m, ArH), 6.89 (1H, d, J = 2.5 Hz, ArH), 6.78 (1H, d, J = 2.6 Hz, ArH), 5.82-5.78 (2H, m, CH=CH), 4.35-4.23 (2H, m, P-CH), 3.09-3.00 (1H, m, CH-O), 1.79-1.74 (1H, m, P-CH_2), 1.29 (9H, s, 3 x CH_3), 1.27 (9H, s, 3 x CH_3), 1.08-1.04 (1H, m, P-CH_2). ^{31}P{^{1}H} NMR (C_6D_6, 162 MHz) δ 144.1 (ap dd, J_{P-P} = 54.8 Hz, J_{P-F} = 4.2 Hz), 5.0 (ap dd, J_{P-P} = 54.8 Hz, J_{P-F} = 3.0 Hz). ^{19}F NMR (C_6D_6, 376 MHz) δ –77.92 (ap dd, J_{P-F} = 4.9 Hz, J_{P-F} = 3.8 Hz). ^{13}C NMR (C_6D_6, 126 MHz) δ 147.35 (d, J = 11.7 Hz ArC), 145.80 (d, J = 2.1 Hz ArC), 143.50 (ArC), 143.14 (ArC), 137.59 (d, J = 4.0 Hz ArC), 137.47 (d, J = 3.2 Hz ArC), 132.53 (ArC), 135.21 (d, J = 3.3 Hz, *C*H=CH), 134.24 (d, J = 4.9 Hz ArC), 133.57 (d, J = 3.2 Hz, CH=*C*H), 130.22 (ArC), 129.35 (ArC), 129.24-126.64 (14 x ArCH), 123.82 (qm, $^1J_{C-F}$ = 283.5 Hz, CF_3), 70.62-70.10 (m, OCH), 51.53 (dd, $^1J_{C-P}$ = 22.0, J = 3.1 Hz, P-CH), 51.27 (d, $^1J_{C-P}$ = 22.0 Hz, P-CH), 35.21 (*C*(CH_3)$_3$), 34.62 (*C*(CH_3)$_3$), 30.71 (C(*C*H_3)$_3$), 30.52 (d, J_{C-P} = 3.5 Hz, C(*C*H_3)$_3$), 21.02 (d, $^1J_{C-P}$ = 31.1 Hz, P-CH_2). HRMS (ES$^+$) $C_{39}H_{39}Cl_2O_3F_3P_2Na$ [MNa]$^+$ m/z: 767.1590 found, 767.1596 required.

5. Preparation of [$PdCl_2$13a], 14.

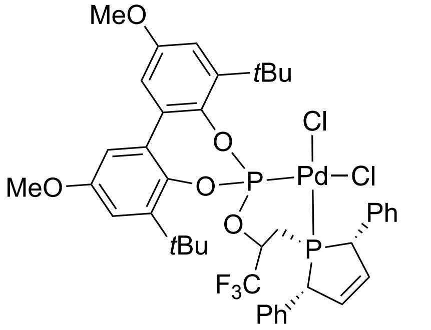

A Schlenk flask was charged with [(C_6H_5CN)$_2$$PdCl_2$] (30 mg, 0.078 mmol) and **13a** (59 mg, 0.08 mmol). The solids were purged with three vacuum/argon cycles and left under an argon atmosphere. CH_2Cl_2 (3.0 mL) was added, and the solution was stirred at room temperature for 3 h. The solvent was then removed under reduced pressure and then 4 ml of a mixture of hexane and ether (3:1) were added and the mixture vigorously stirred for 10 minutes. The resulting solid was filtered off and washed with 3 ml of the same solvents mixture to afford the required complex as an off-white solid. Further purification was achieved by dissolving back the complex in CH_2Cl_2 (2 ml) and filtering through a plug of celite. Off-white solid (65 mg, 0.071 mmol, 91%). X-Ray quality crystals were grown by layering hexane over a $CDCl_3$ solution of the complex. (**Figure S.5.1**). ^{1}H NMR ($CDCl_3$, 500 MHz) δ 7.36-7.50 (10H, m, ArH), 7.00 (1H, d, J = 2.7 Hz, ArH), 6.87 (1H, d, J = 2.8 Hz, ArH), 6.67 (1H, d, J = 2.9 Hz, ArH), 6.60 (1H, d, J = 2.9 Hz, ArH), 6.38-6.29 (2H, m, CH=CH), 5.87-5.81 (2H, m, P-CH), 3.82 (3H, s, OCH_3), 3.77 (3H, s, OCH_3), 3.16-3.04

(1H, m, CH-O), 1.76-1.67 (1H, m, P-CH_2), 1.64 (9H, s, 3 x CH_3), 1.15 (9H, s, 3 x CH_3), 0.97-0.91 (1H, m, P-CH_2). $^{31}P\{^1H\}$ NMR ($CDCl_3$, 202 MHz) δ 99.14 (d, $^2J_{P\text{-}P}$ = 12.6 Hz), 45.90 (d, $^2J_{P\text{-}P}$ = 12.6 Hz). ^{19}F NMR ($CDCl_3$, 471 MHz) δ –79.88 (d, $J_{P\text{-}F}$ = 5.0 Hz). ^{13}C NMR ($CDCl_3$, 126 MHz) δ 156.70 (ArC), 156.53 (ArC), 142.50 (d, J = 10.4 Hz ArC), 141.87 (d, J = 3.3 Hz, ArC), 141.39 (d, J = 16.1 Hz, ArC), 140.85 (d, J = 4.8 Hz, ArC), 134.02 (d, J = 8.2 Hz ArC), 133.50 (d, J = 8.1 Hz ArC), 131.60 (ArC), 130.35 (ArC), 134.43 (d, J = 4.0 Hz, *C*H=CH), 132.65 (d, J = 4.1 Hz, CH=*C*H), 129.94 (4 x ArCH), 128.84 (d, J = 3.0 Hz ArCH), 128.50-128.42 (m, 5 x ArCH), 121.46 (qm, $^1J_{C\text{-}F}$ = 280.9 Hz, CF_3), 115.21 (ArCH), 114.77 (ArCH), 114.12 (ArCH), 113.97 (ArCH), 71.43-70.55 (m, OCH), 55.67 (OCH_3), 55.60 (OCH_3), 52.65 (d, $^1J_{C\text{-}P}$ = 26.6, J = 3 Hz, P-CH), 52.45 (d, $^1J_{C\text{-}P}$ = 23.9 Hz, P-CH), 35.92 ($C(CH_3)_3$), 35.17 ($C(CH_3)_3$), 32.50 ($C(CH_3)_3$), 31.15 ($C(CH_3)_3$), 15.15 (dd, $^1J_{C\text{-}P}$ = 25.2 Hz, $^3J_{C\text{-}F}$ = 5.7 Hz, P-CH_2). HRMS (ES^+) $C_{41}H_{45}O_5ClF_3P_2Pd$ $[M\text{-}Cl]^+$ m/z: 877.1413 found, 877.1412 required.

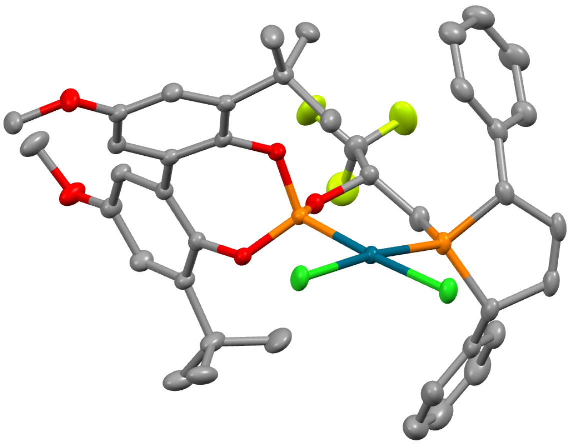

Figure S.5.1. View of an independent molecule in the structure of complex **14**. Hydrogen atoms omitted for clarity.

6. 1D gs-NOESY Spectra of 5 and 9

Figure S.6.1. a) ^{1}H NMR spectrum of **5**. b) and c) 1D gs-NOESY (selnogpzs) with mixing time d8 = 800 ms and selective irradiation set on H^1. Strong NOE signal observed for H^2 in agreement to a *cis* stereochemistry of H^1 and H^2.

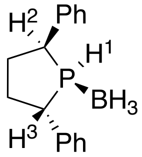

5

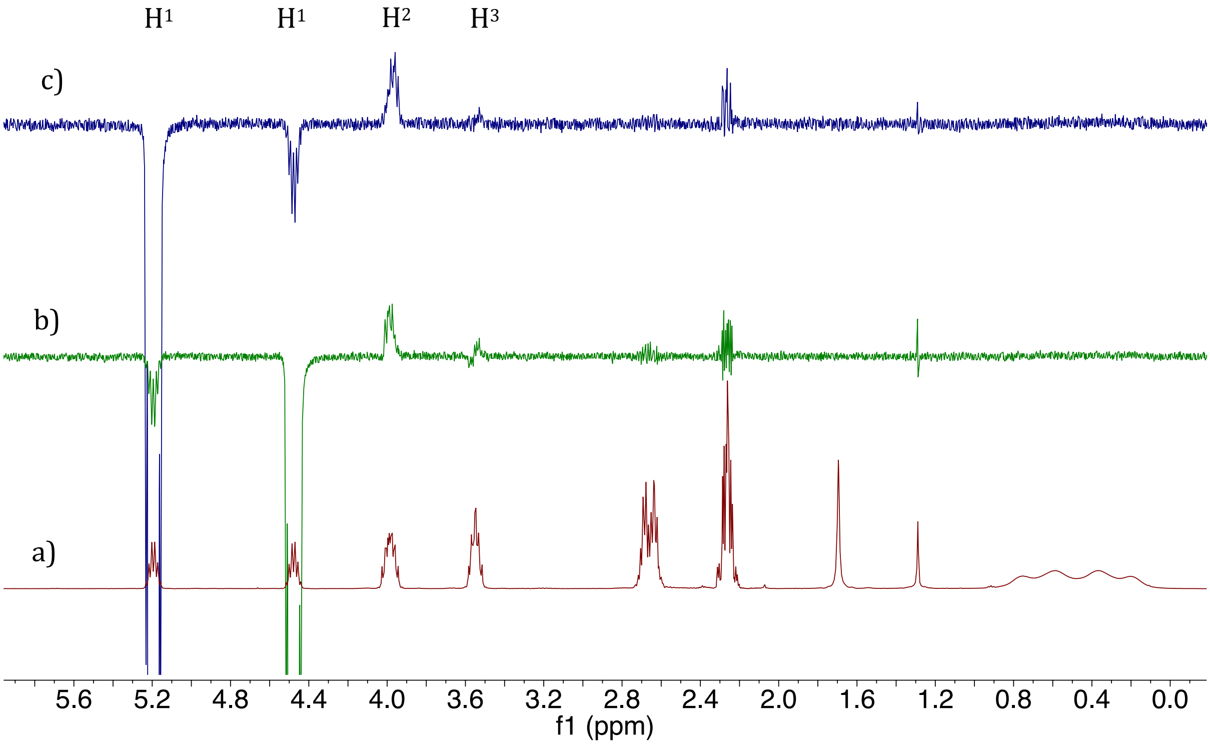

Figure S.6.2. a) ^{1}H NMR spectrum of **5**. b) 1D gs-NOESY (selnogpzs) with mixing time d8 = 800 ms and selective irradiation set on H^3. No NOE signal observed for H^3 with H^1 c) 1D gs-NOESY (selnogpzs) with mixing time d8 = 800 ms and selective irradiation set on H^2. Strong NOE signal observed for H^1, confirming the *cis* relative stereochemistry of H^1 and H^2.

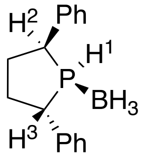

5

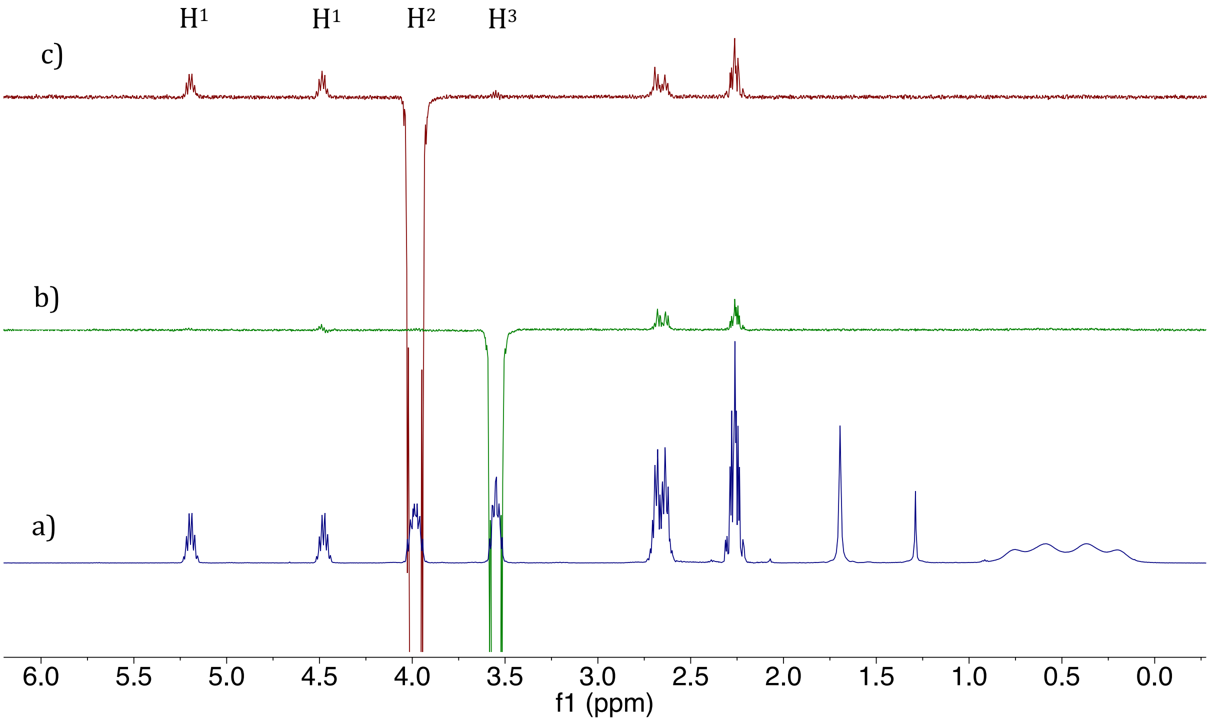

Figure S.6.3. a) ^{1}H NMR spectrum of **9**. b) and c) 1D gs-NOESY (selnogpzs) with mixing time d8 = 800 ms and selective irradiation set on H^1. Strong NOE signal observed for ArCH of the phenyl groups which is consistent to a *trans* stereochemistry of H^1 and H^2. Since H^1 and H^2 are coupled spins (J = 8.2 Hz) the antiphase doublet observed for proton H^2 is likely ZQC artefact.

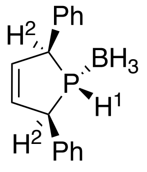

9

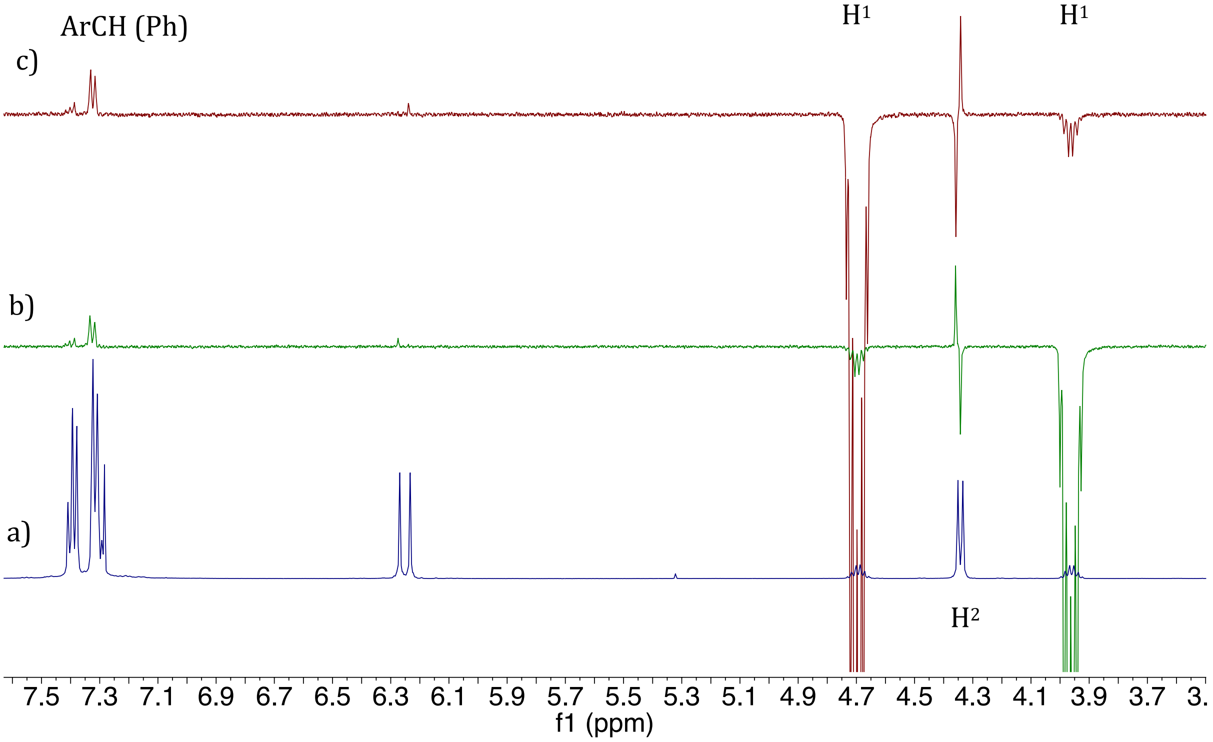

Figure S.6.3. a) ^{1}H NMR spectrum of **9**. b) 1D gs-NOESY (selnogpzs) with mixing time d8 = 800 ms and selective irradiation set on H^2. Since H^1 and H^2 are coupled spins (J = 8.2 Hz) the antiphase signal observed for proton H^1 is likely ZQC artefact.

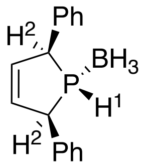

9

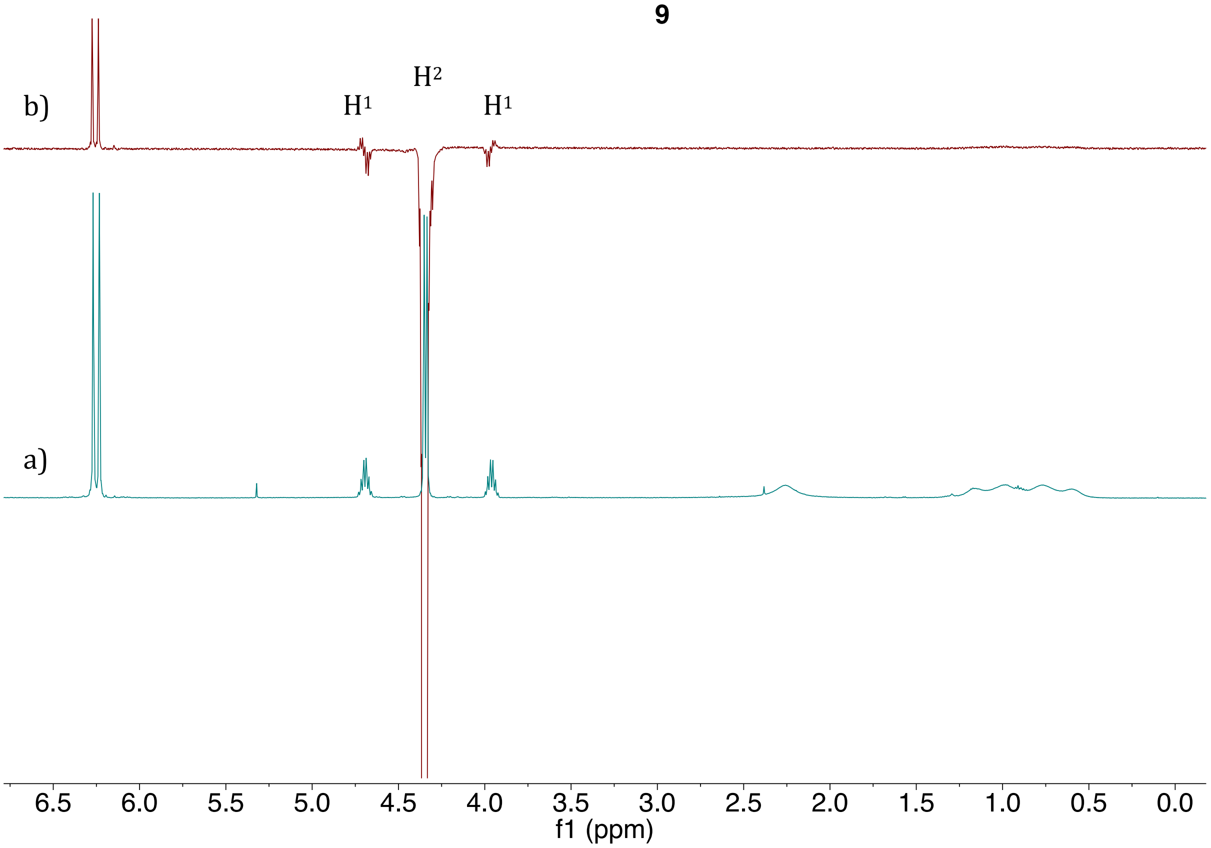

7. Coordination and stability studies.

7.1 NMR spectroscopic investigation of the coordination mode of [RhH(CO)$_2$(*tropos*,*meso*)-13a)].

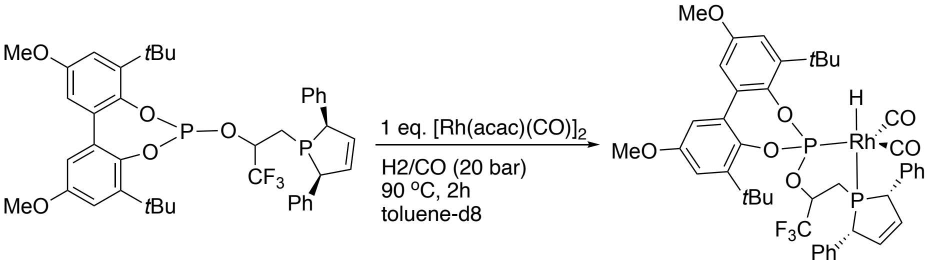

Scheme S.7.1. Preparation of [RhH(CO)$_2$(*tropos*,*meso*)-**13a**].

General procedure

A vial containing a stirring bar was charged with [Rh(acac)(CO)$_2$] (15.7 mg, 0.061 mmol), (*tropos*,*meso*)-**13a**) (45 mg, 0.061mmol). The vial was sealed with a crimp cap, purged with two vacuum/argon cycles and left under a N_2 atmosphere. Toluene-d8 (2.5 mL) was added, two needles were pierced into the septum of the vial cap and the vial was placed into the autoclave (The autoclave was previously purged with three vacuum/N_2 cycles). The autoclave was then purged three times with syngas (50/50, CO/H_2), pressurised to 20 bar, immersed into an oil bath preheated at 90 °C and stirred at 700 r.p.m for the desired time (2h). After this time, the autoclave was cooled down to room temperature by partial immersion in cold water and the pressure released to 1 atm of syngas. A sample was taken via syringe, introduced in a NMR tube previously purged with syngas and analysed by ^{1}H NMR (toluene-d8) and ^{31}P{^{1}H}-NMR (toluene-d8).

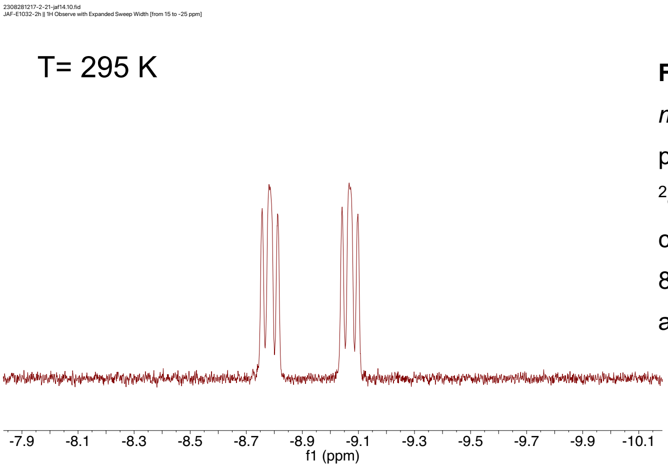

Fig S.7.1. **^{1}H-NMR** spectrum of [RhH(CO)$_2$(*tropos*, *meso*)-**13a**)] showing the hydride region at rt. The presence of a doublet of doublets of doublets and the $^2J_{P\text{-}H}$ values suggest an equatorial-axial (ea) coordination mode. **^{1}H NMR** (400 MHz, Toluene-*d*$_8$) δ -8.93 (ddd, $^2J_{P\text{-}H}$ = 114.7 Hz, *cis* $^2J_{P\text{-}H}$ and $^1J_{Rh\text{-}H}$ = 12.4 and 10.0 Hz, 1H)

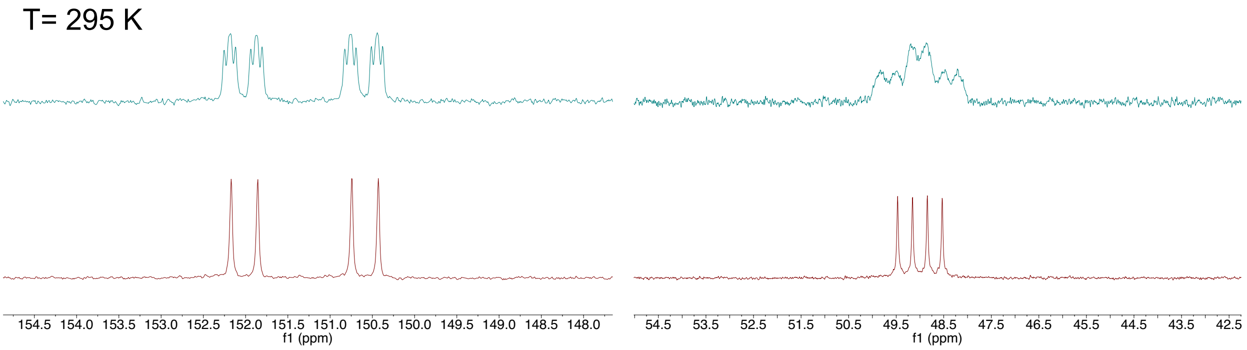

Fig S.7.2. ^{31}P{^{1}H}-NMR and **^{31}P-NMR** spectra of [RhH(CO)$_2$(*tropos*, *meso*)-**13a**)] at rt. ^{31}P{^{1}H}-NMR (162 MHz, Toluene-d_8) δ 151.3 (dd, $^{1}J_{Rh\text{-}P}$ = 231 Hz, $^{2}J_{P\text{-}P}$ = 51 Hz), 49.00 (dd, $^{1}J_{Rh\text{-}P}$ = 101 Hz, $^{2}J_{P\text{-}P}$ = 51 Hz). ^{31}P NMR (162 MHz, Toluene-d_8) δ 151.3 (ddt, $^{1}J_{Rh\text{-}P}$ = 231 Hz, $^{2}J_{P\text{-}P}$ = 51 Hz, *cis* $^{2}J_{P\text{-}H}$ 11 Hz), 49.00 (td, $^{1}J_{Rh\text{-}P}$ = 104, *trans* $^{2}J_{P\text{-}H}$ 103, $^{2}J_{P\text{-}P}$ = 51 Hz).

The presence of a double of doublets suggests an equatorial-axial (ea) coordination mode. Comparing the ^{31}P{^{1}H} and ^{31}P-NMR, a larger $^{2}J_{P\text{-}H}$ can be observed in the phosphine region suggesting a preferential equatorial-axial (ea) coordination mode with the phosphine *trans* to the hydride. Both apical isomers exist in a temperature dependant dynamic equilibrium. The magnitude of the $^{2}J_{P\text{-}H}$ in the phosphine region (103 Hz), indicates that the equilibrium is largely displaced towards the isomer having the phospholene trans to the hydride.

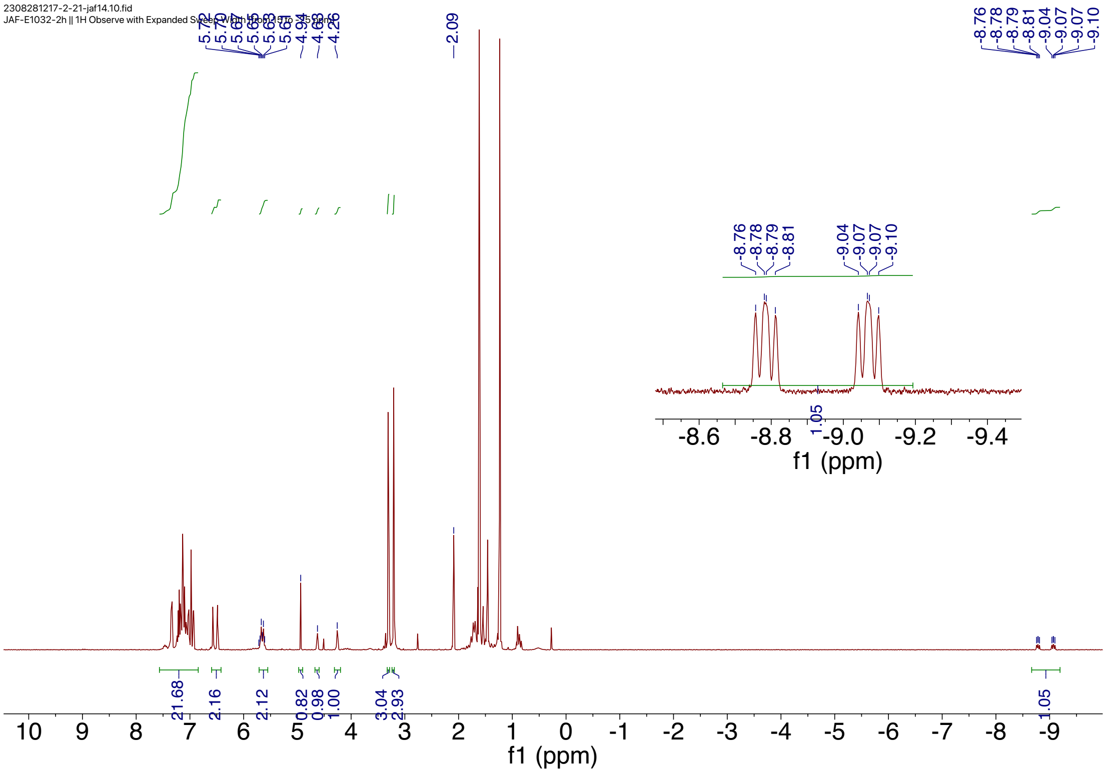

Figure S.7.3. ^{1}H-NMR spectrum of [RhH(CO)$_2$(*tropos*,*meso*)-**13a**] after 2h at 90 °C.

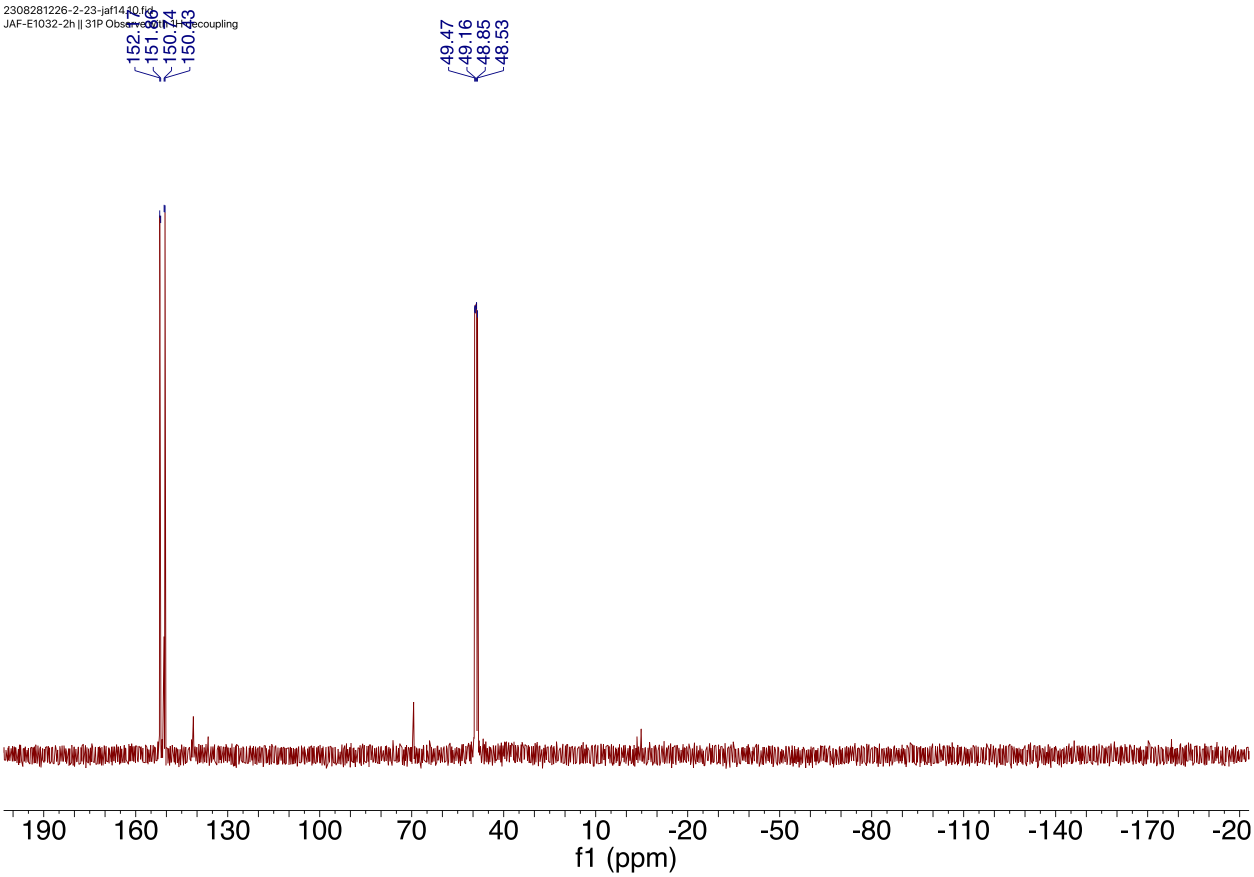

Figure S.7.4. ^{31}**P{^{1}H}-NMR** spectrum of [RhH(CO)$_2$(*tropos*,*meso*)-**13a**] after 2h at 90 °C.

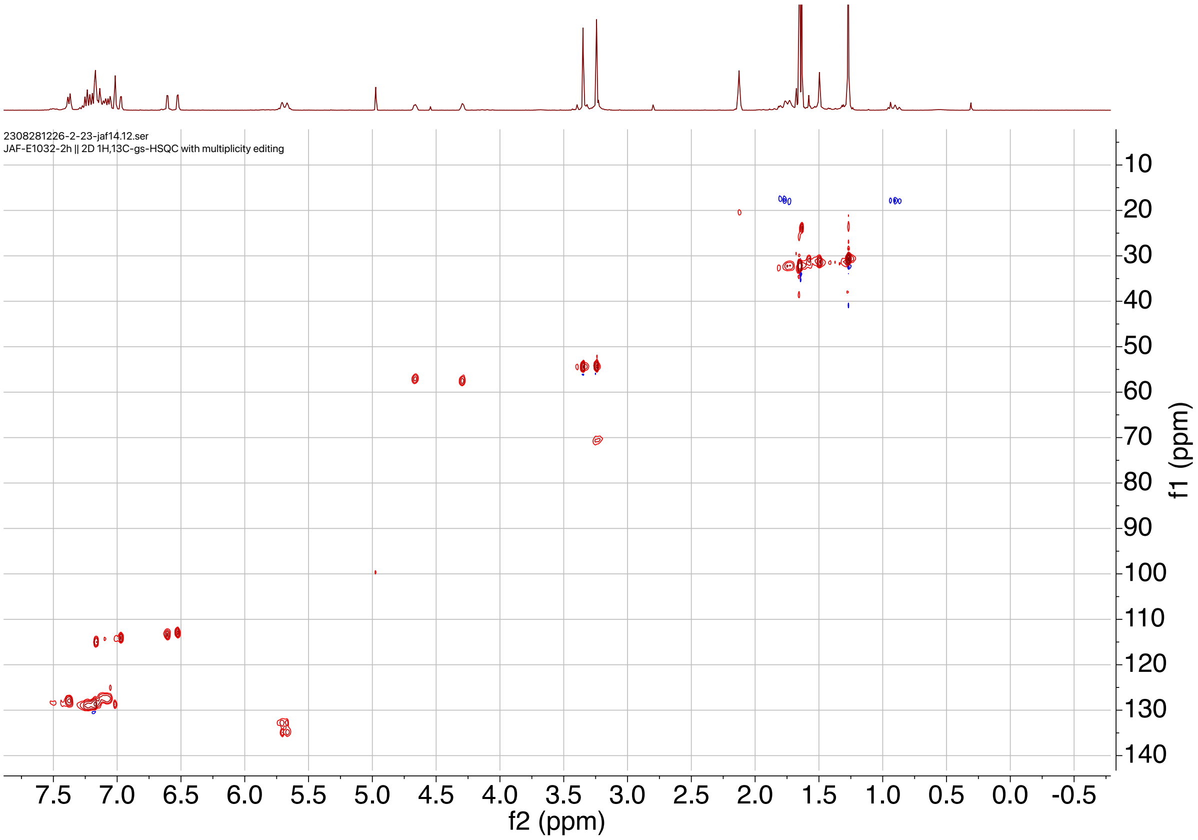

Figure S.7.5. HSQC-NMR spectrum of [RhH(CO)$_2$(*tropos*,*meso*)-**13a**] after 2h at 90 °C.

7.2 NMR spectroscopic investigation of the stability under syngas at 90 °C of [RhH(CO)$_2$(*tropos*,*meso*)-13a)]. One week-long experiment.

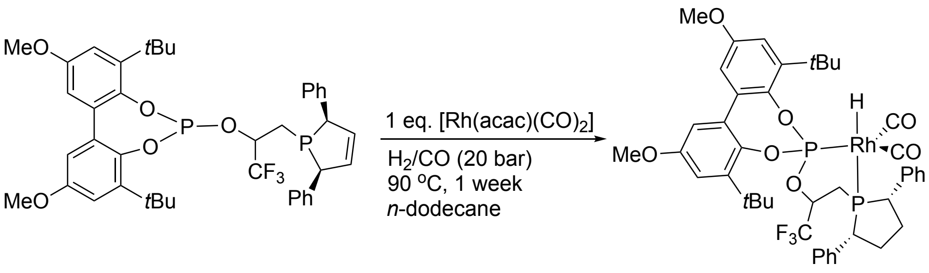

Scheme S.7.2. Preparation of [RhH(CO)$_2$(*tropos*,*meso*-**13a-H2**)]

[RhH(CO)$_2$(*tropos*,*meso*)-**13a**)] complex was formed according to the general procedure. After 1 week there was a clear pale-yellow solution. A sample of the solution was taken via syringe, introduced in a NMR tube previously purged with syngas and analysed by ^{1}H NMR and ^{31}P-NMR using a benzene-d6 capillary. The solution did not contain any phosphorus-bearing species. The orange solid was decanted, dissolved in d8-toluene and analysed by ^{1}H NMR and ^{31}P-NMR. Both NMR showed the presence in solution of a new Rh-complex, [RhH(CO)$_2$(*tropos*,*meso*)-**13a-H2**)] in excellent purity even after a whole week at 90 °C.

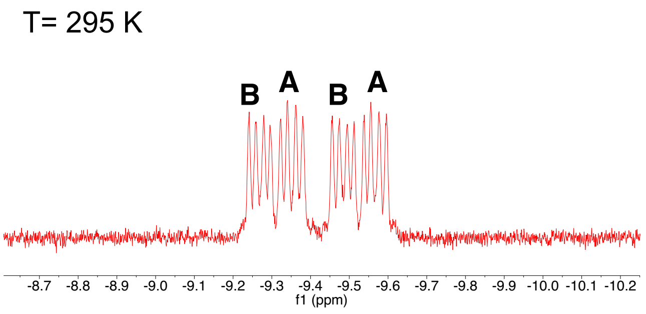

Figure S.7.6. ^{1}H-NMR spectrum of [RhH(CO)$_2$(*tropos*,*meso*)-**13a-H2**)], after one week stirring under syngas atmosphere at 90 °C, showing the hydride region at rt. The presence of two doublet of doublets of doublets and the $^{2}J_{P\text{-}H}$ values suggest two isomers with an equatorial-axial (ea) coordination mode for both of them. **^{1}H NMR** (500 MHz, Toluene-*d*$_8$) δ Isomer B: -9.38 (ddd, $^{2}J_{P\text{-}H}$ = 107.5 Hz, *cis* $^{2}J_{P\text{-}H}$ and $^{1}J_{Rh\text{-}H}$ = 18.9 and 8.9 Hz, 1H); Isomer A: -9.46 (ddd, $^{2}J_{P\text{-}H}$ = 108.0 Hz, *cis* $^{2}J_{P\text{-}H}$ and $^{1}J_{Rh\text{-}H}$ = 20.0 and 9.2 Hz, 1H).

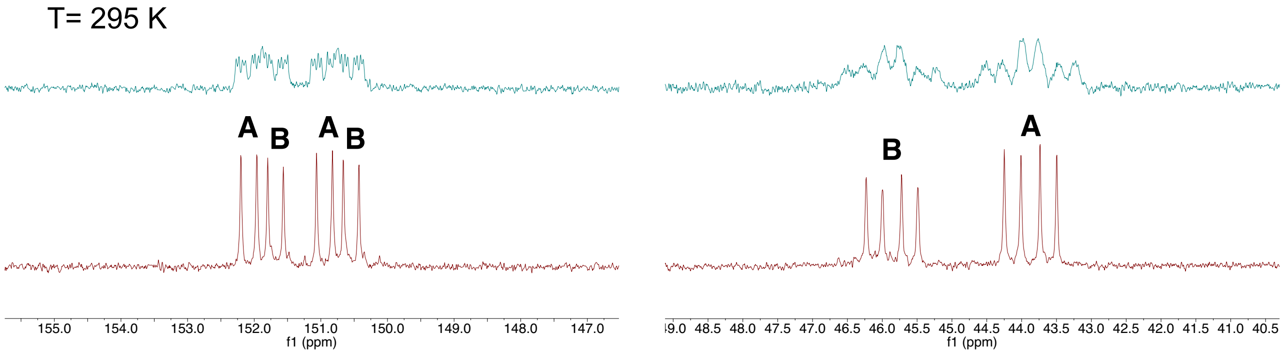

Figure S.7.7. ^{31}P{^{1}H}-NMR and **^{31}P-NMR** spectra of [RhH(CO)$_2$(*tropos*,*meso*)-**13a-H2**)] after one week stirring under syngas atmosphere at 90 °C. ^{31}P{^{1}H}-NMR (202 MHz, Toluene-*d*$_8$) δ Isomer A: 151.5 (dd, $^{1}J_{Rh\text{-}P}$ = 230 Hz, $^{2}J_{P\text{-}P}$ = 48 Hz), 43.87 (dd, $^{1}J_{Rh\text{-}P}$ = 104 Hz, $^{2}J_{P\text{-}P}$ = 48 Hz). Isomer B: 151.5 (dd, $^{1}J_{Rh\text{-}P}$ = 230 Hz, $^{2}J_{P\text{-}P}$ = 47 Hz), 45.86 (dd, $^{1}J_{Rh\text{-}P}$ = 103 Hz, $^{2}J_{P\text{-}P}$ = 47 Hz).

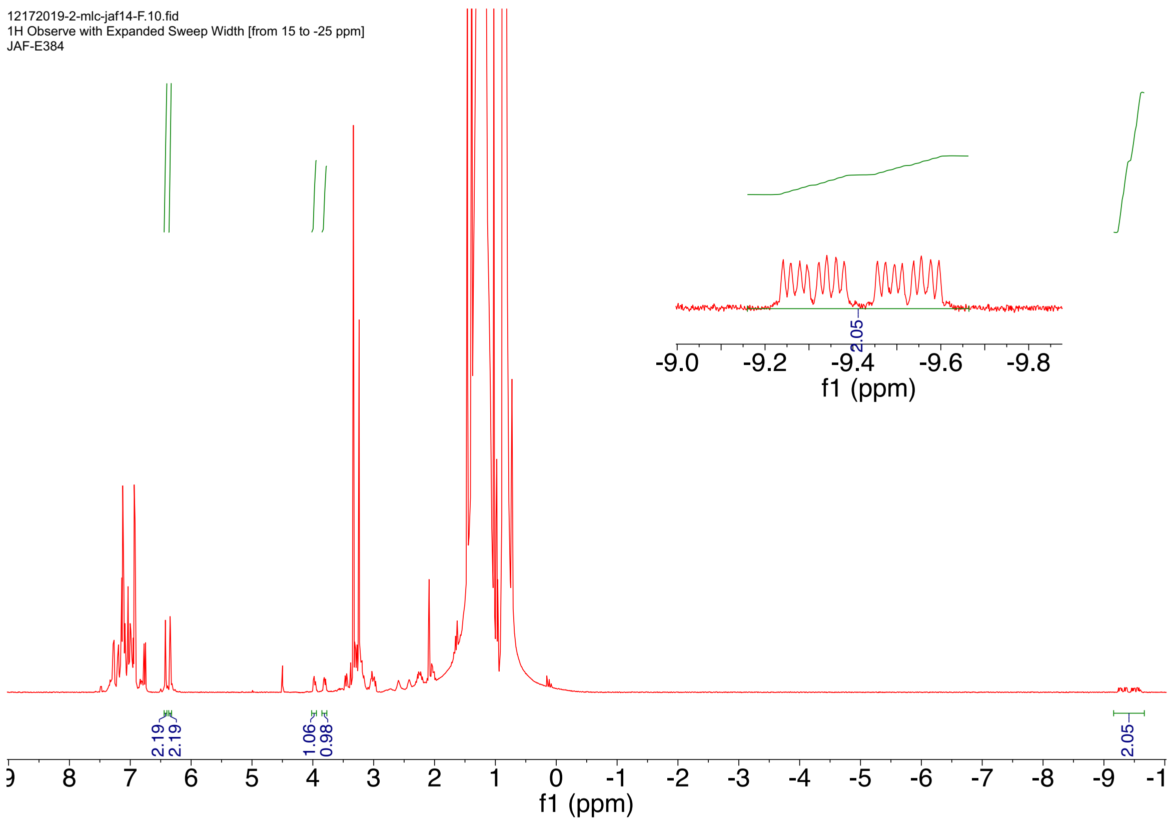

Figure S.7.8. **^{1}H-NMR** spectrum of [RhH(CO)$_2$(*tropos*,*meso*)-**13a-H2**)] after one week stirring under syngas atmosphere at 90 °C.

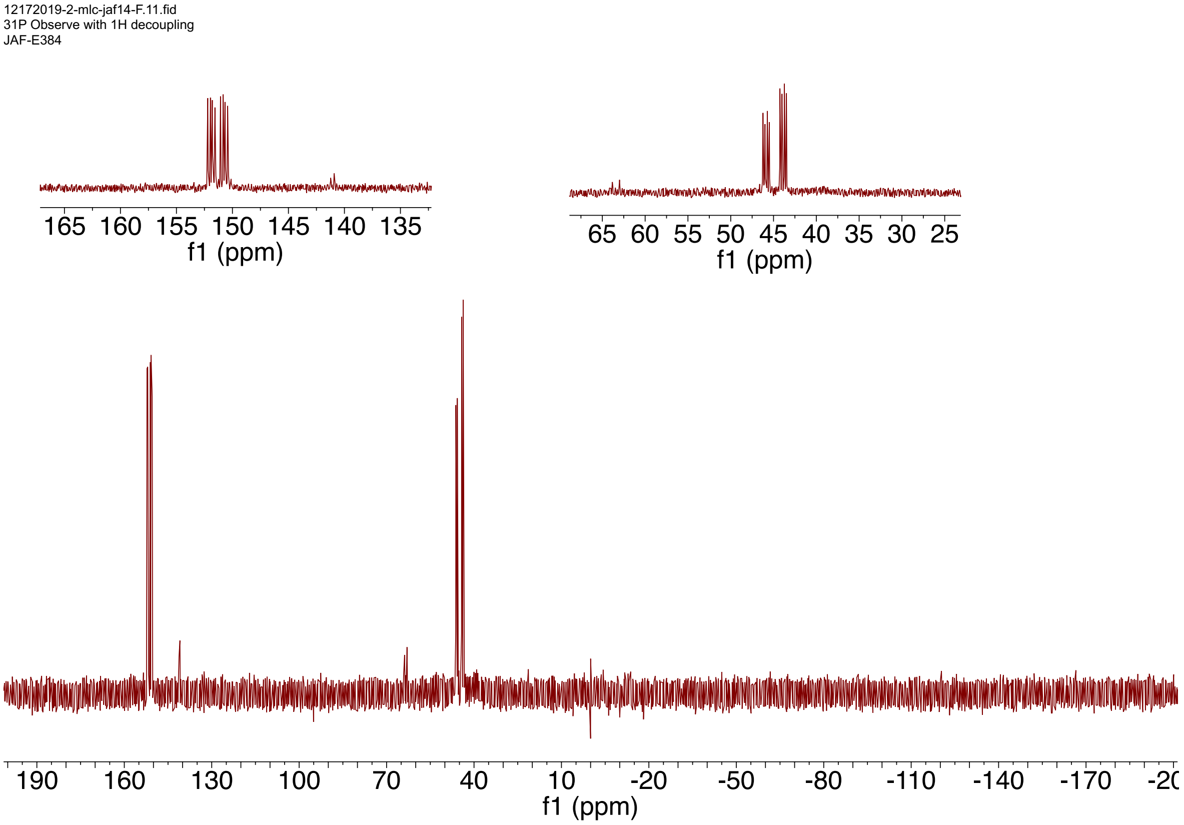

Figure S.7.9. **^{31}P{^{1}H}-NMR** spectrum of [RhH(CO)$_2$(*tropos*,*meso*)-**13a-H2**)] after one week stirring under syngas atmosphere at 90 °C.

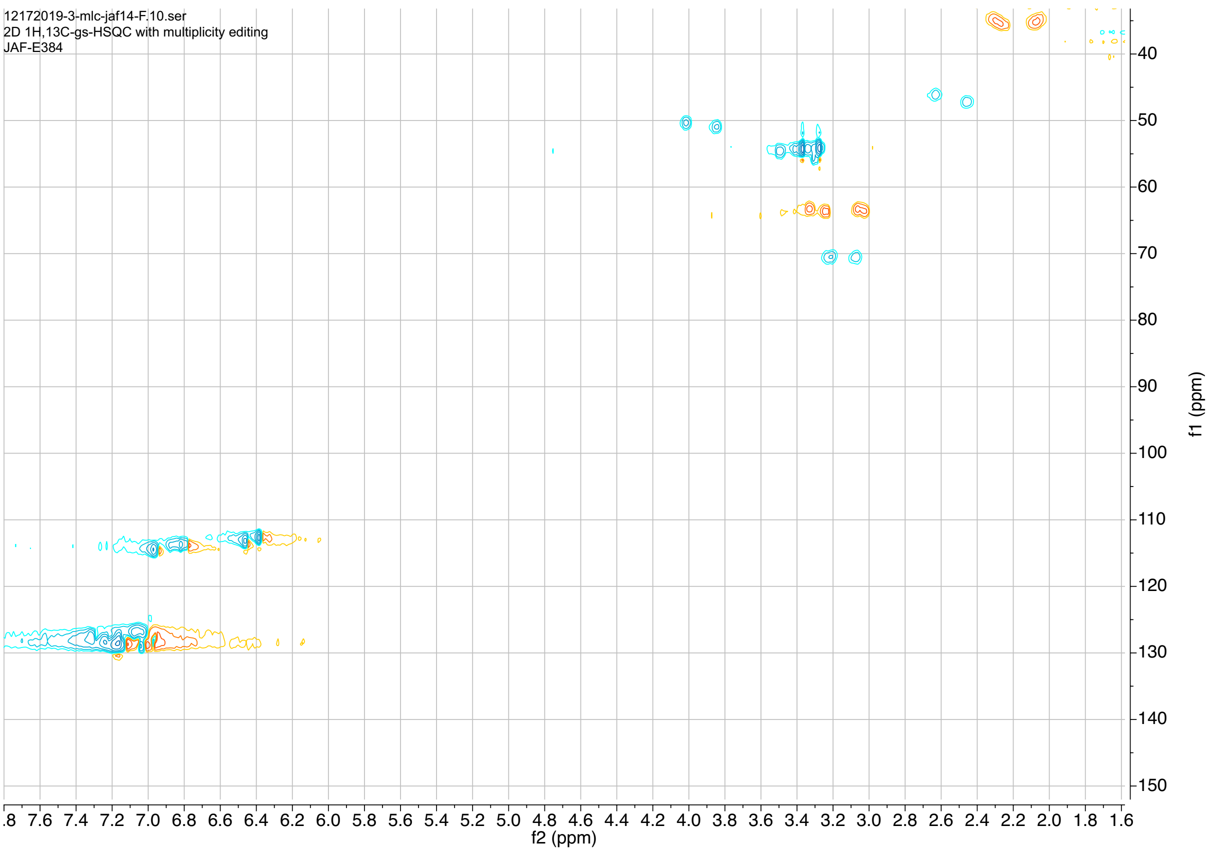

Figure S.7.10. **HSQC-NMR** spectrum of [RhH(CO)$_2$(*tropos*,*meso*)-**13a-H2**] after one week stirring under syngas atmosphere at 90 °C.

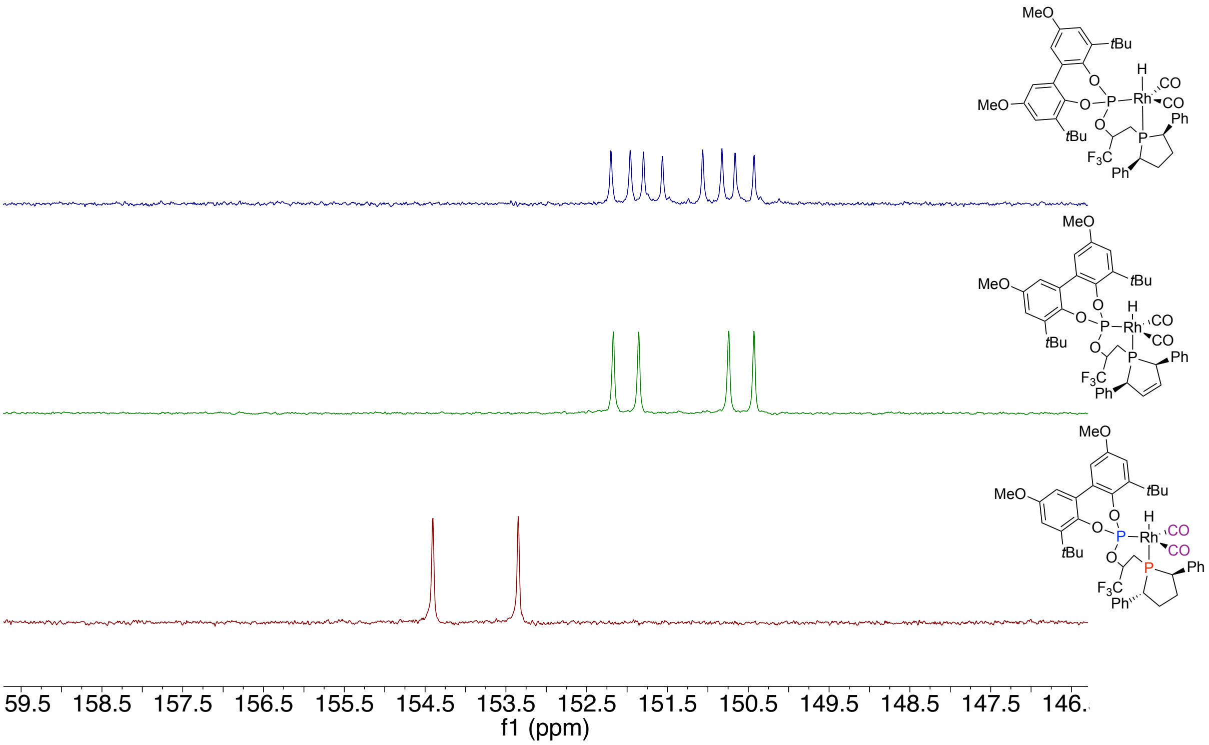

Figure S.7.11. **^{31}P{^{1}H}-NMR** spectra (phosphite region) of [RhH(CO)$_2$(*tropos*,*meso*)-**13a-H2**)], [RhH(CO)$_2$(*tropos*,*meso*)-**13a**)] and [RhH(CO)$_2$(*tropos*,*trans*)-**1**)].

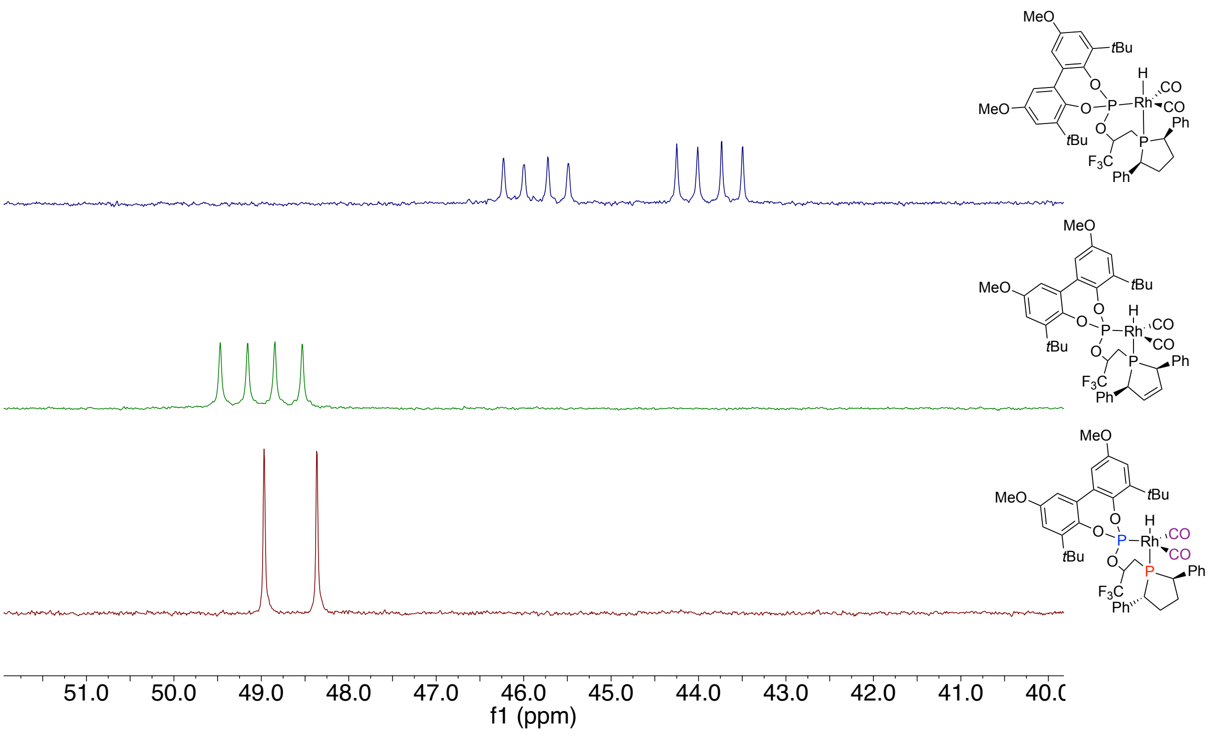

Figure S.7.12. ^{31}**P{^{1}H}-NMR** spectra (phosphine region) of [RhH(CO)$_2$(*tropos*,*meso*)-**13a-H2**)], [RhH(CO)$_2$(*tropos*,*meso*)-**13a**)] and [RhH(CO)$_2$(*tropos*,*trans*)-**1**)].

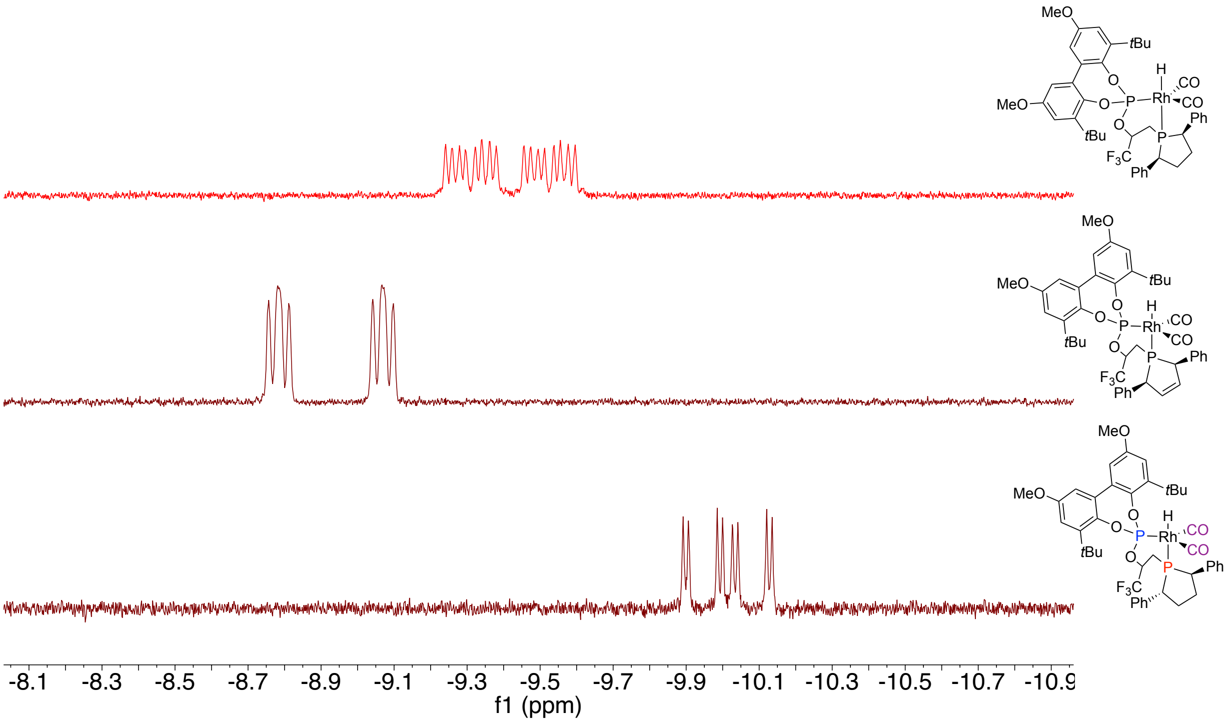

Figure S.7.13. ^{1}**H-NMR** spectra (hydride region) of [RhH(CO)$_2$(*tropos*,*meso*)-**13a-H2**)], [RhH(CO)$_2$(*tropos*,*meso*)-**13a**)] and [RhH(CO)$_2$(*tropos*,*trans*)-**1**)].

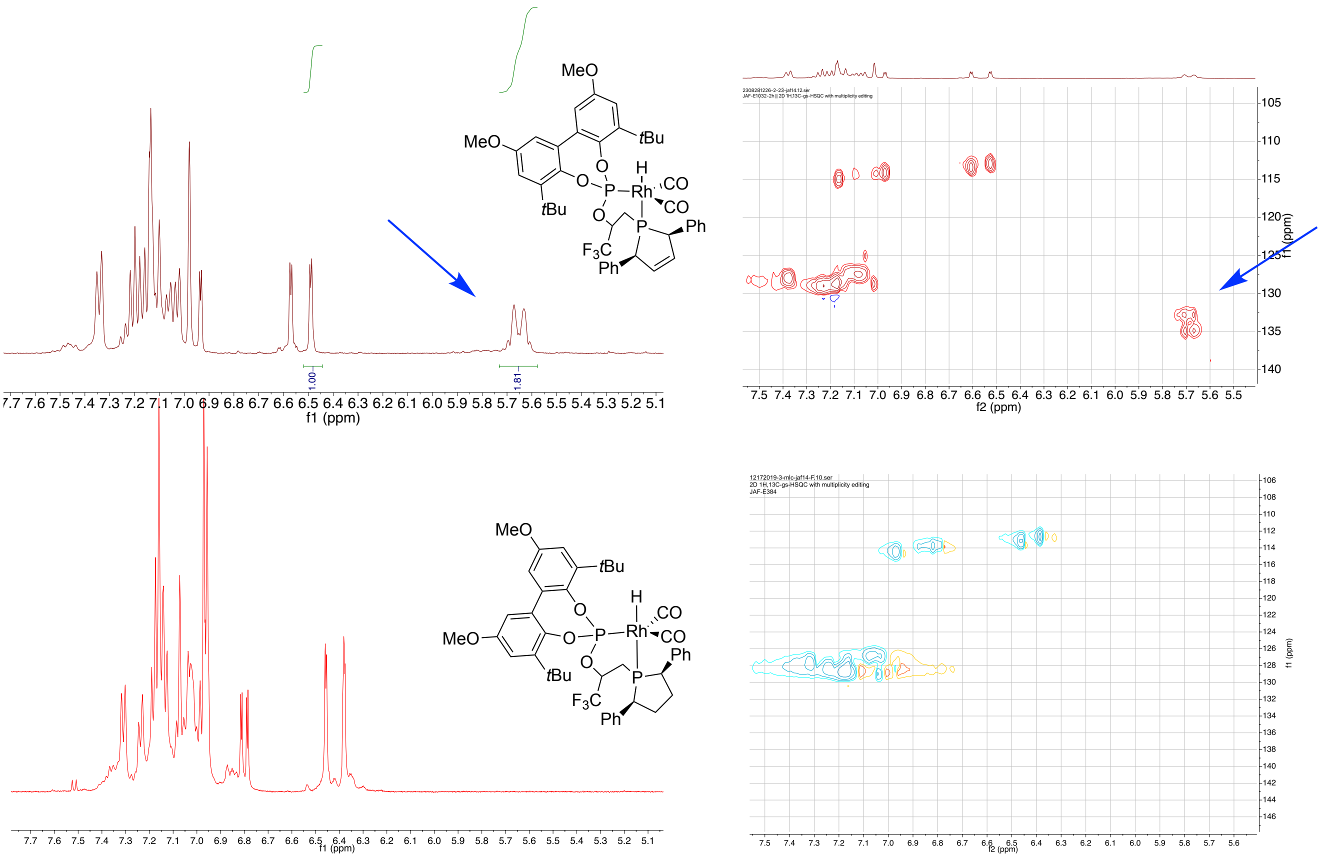

1H NMR (500 MHz, Toluene-d8)

1H13C HSQC NMR (Toluene-d8)

Figure S.7.14. **^{1}H-NMR** and **^{1}H^{13}C-HSQC NMR** spectra (alkene region) [RhH(CO)$_2$(*tropos*,*meso*)-**13a**)] and [RhH(CO)$_2$(*tropos*,*meso*)-**13a-H2**)], Data suggest that the double bond gets hydrogenated overtime.

8. HPIR spectroscopic activation study of phospholane-phosphites 13a-13c with [Rh(acac)(CO)$_2$].

Fig S.8.1. HPIR spectra of **[RhH(CO)$_2$(*tropos*,*meso*)-13a]**. Conditions: Rh:L = 1:1.25 (C_{Rh} 1 mM in dodecane), T = 90 °C, P= 20 bar, CO:H_2 1:1. Full activation achieved after 40 min.

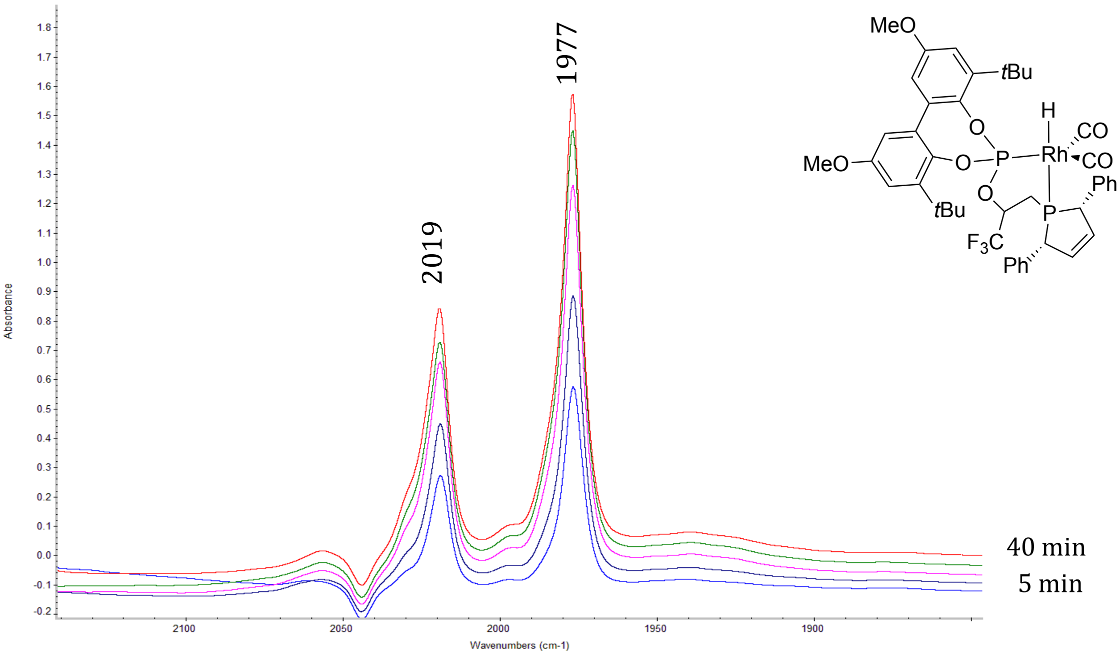

Fig S.8.2. HPIR spectra of **[RhH(CO)$_2$(*tropos*,*meso*)-13b]**. Conditions: Rh:L = 1:1.25 (C_{Rh} 1 mM in dodecane), T = 90 °C, P= 20 bar, CO:H_2 1:1. Full activation achieved after 45 min.

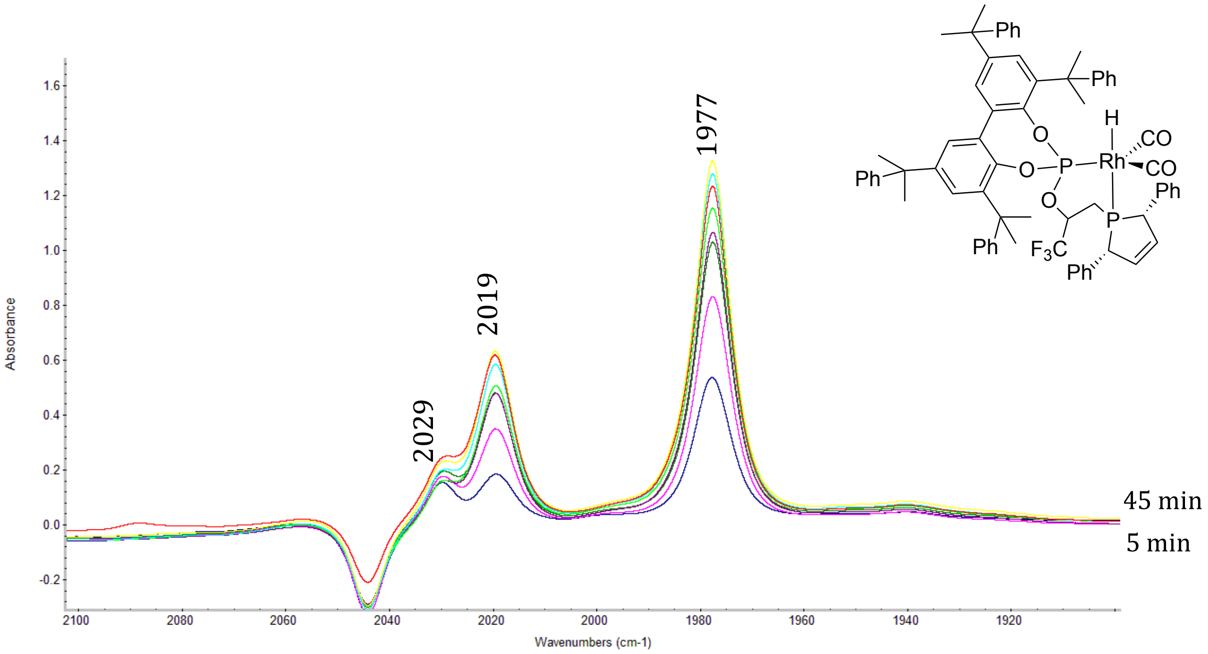

Fig S.8.3. HPIR spectra of **[RhH(CO)$_2$(*tropos*,*meso*)-13c]**. Conditions: Rh:L = 1:1.25 (C_{Rh} 1 mM in dodecane), T = 90 °C, P= 20 bar, CO:H_2 1:1. Full activation achieved after 25 min.

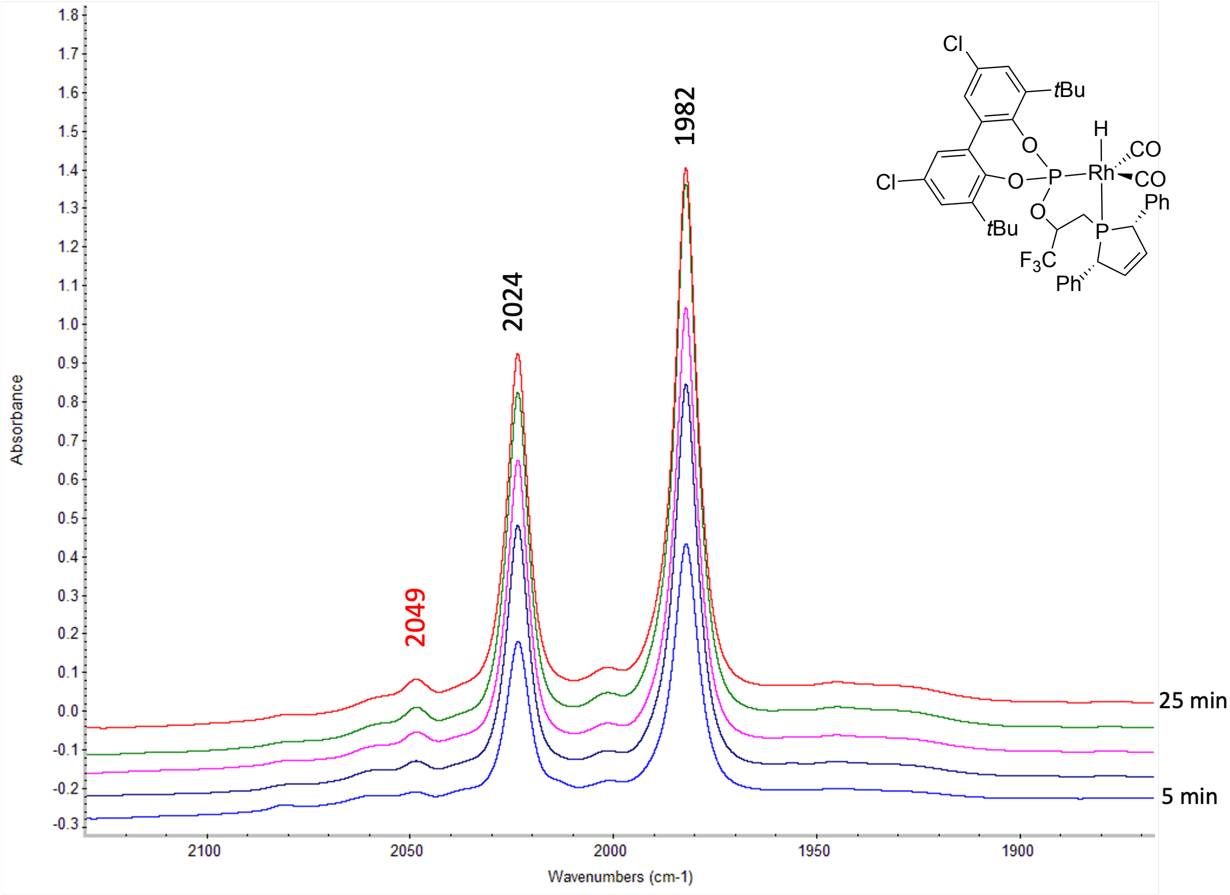

9. HPIR spectroscopic thermal stability study of phospholane-phosphites 13a-13c with [Rh(acac)(CO)2].

Fig S.9.1. HPIR spectra of **[RhH(CO)2(*tropos,meso*)-13a]**. Conditions: Rh:L = 1:1.25 (C_{Rh} 1 mM in dodecane), T = 90 °C, P= 20 bar, CO:H_2 1:1.

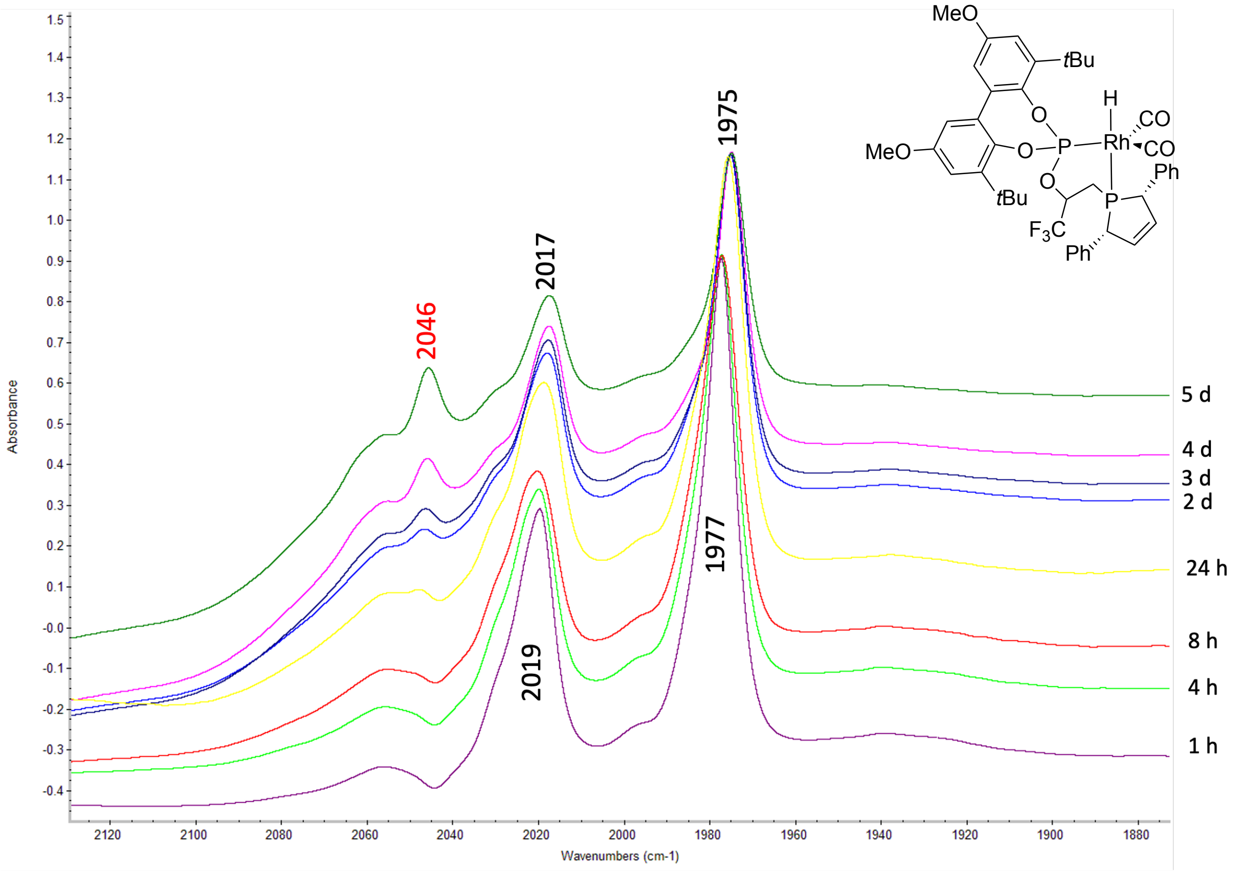

The drifting of the baseline observed in the spectra of **[RhH(CO)2(*tropos,cis*)-13a]** is not due to Rh deposition but a slight pressure change that occurred during this specific experiment due to a slight technical failure. This was confirmed in another experiment showing the reversible change in the drifting of the baseline observed in the IR spectra.[18]

Fig S.9.2. HPIR spectra of **[RhH(CO)$_2$(*tropos,meso*)-13a]**. Conditions: Rh:L = 1:1.25 (C_{Rh} 1 mM in dodecane), T = 105 °C, P= 20 bar, CO:H_2 1:1.

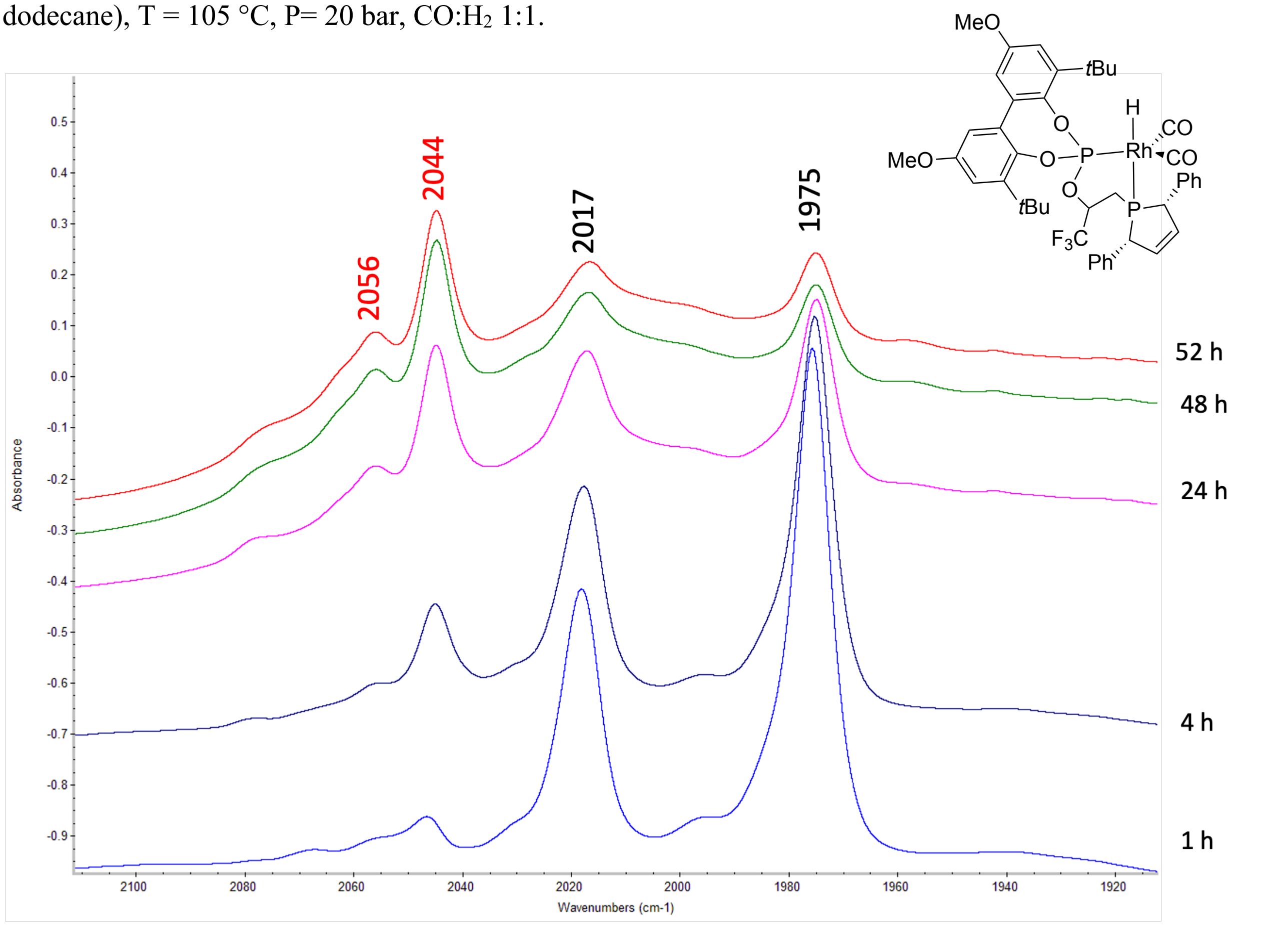

Fig S.9.3. HPIR spectra of **[RhH(CO)$_2$(*tropos,meso*)-13b]**. Conditions: Rh:L = 1:1.25 (C_{Rh} 1 mM in dodecane), T = 105 °C, P= 20 bar, CO:H_2 1:1.

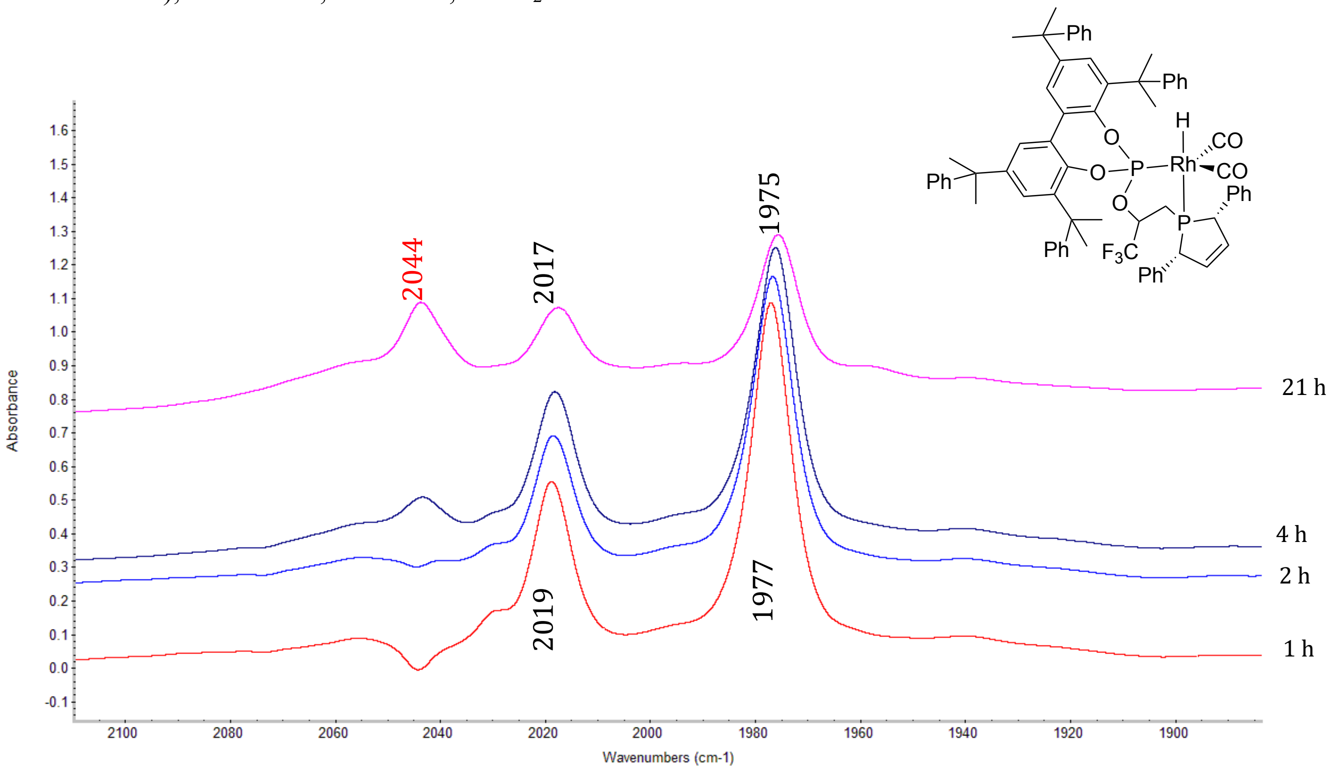

Fig S.9.4. HPIR spectra of **[RhH(CO)$_2$(*tropos*,*cis*)-13c]**. Conditions: Rh:L = 1:2.0 (C_{Rh}1 mM in dodecane), T = 105 °C, P_i= 20 bar, CO:H_2 1:1.

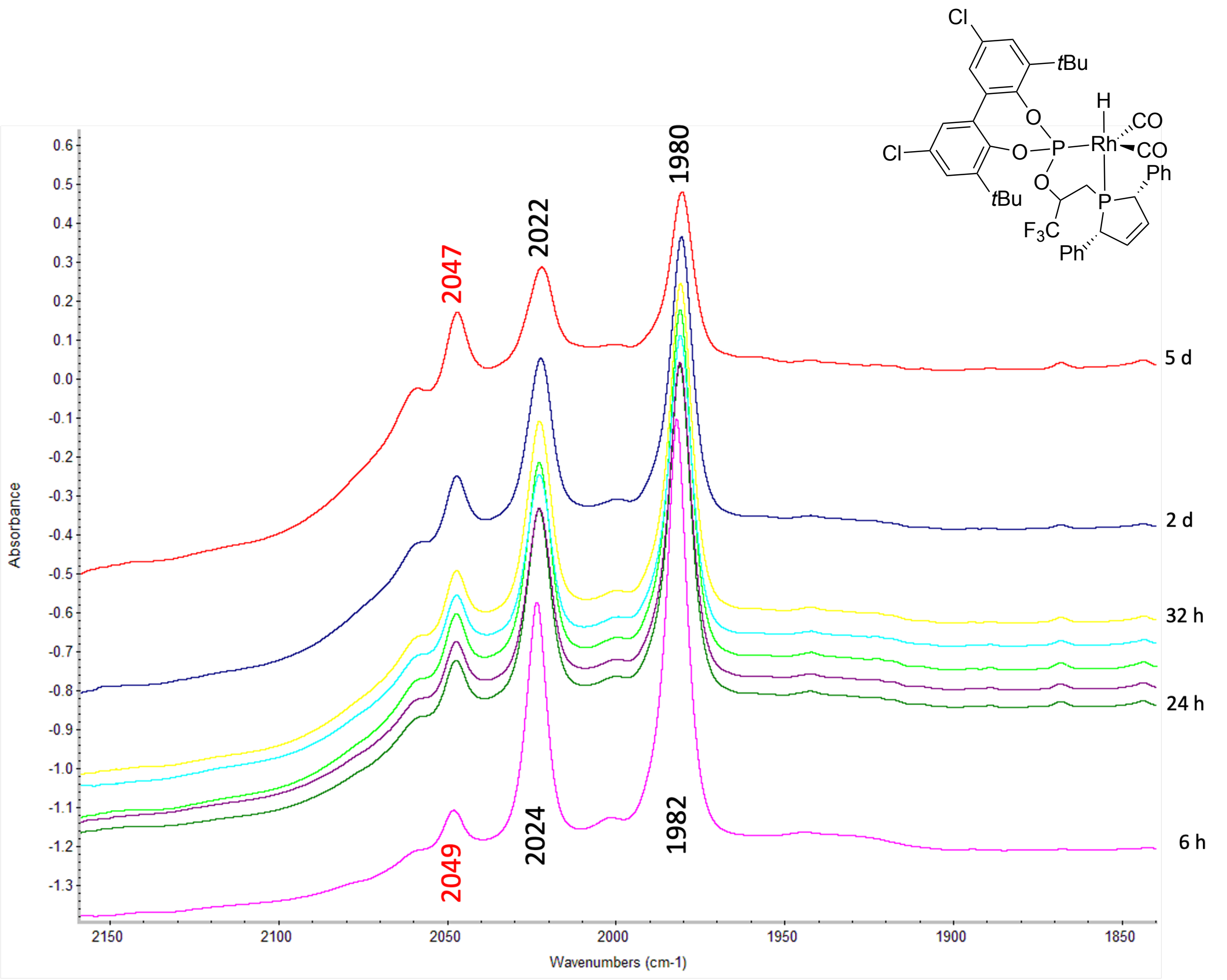

10. NMRs of selected compounds

Fig S.10.1. Borane protected-(*meso*)-2,5-diphenyl-2,5-dihydro-1*H*-phosphole, **9**, ^{1}H NMR (below), ^{13}C NMR (bottom), $^{31}P\{^{1}H\}$NMR (top next page) and ^{31}P NMR (bottom next page).

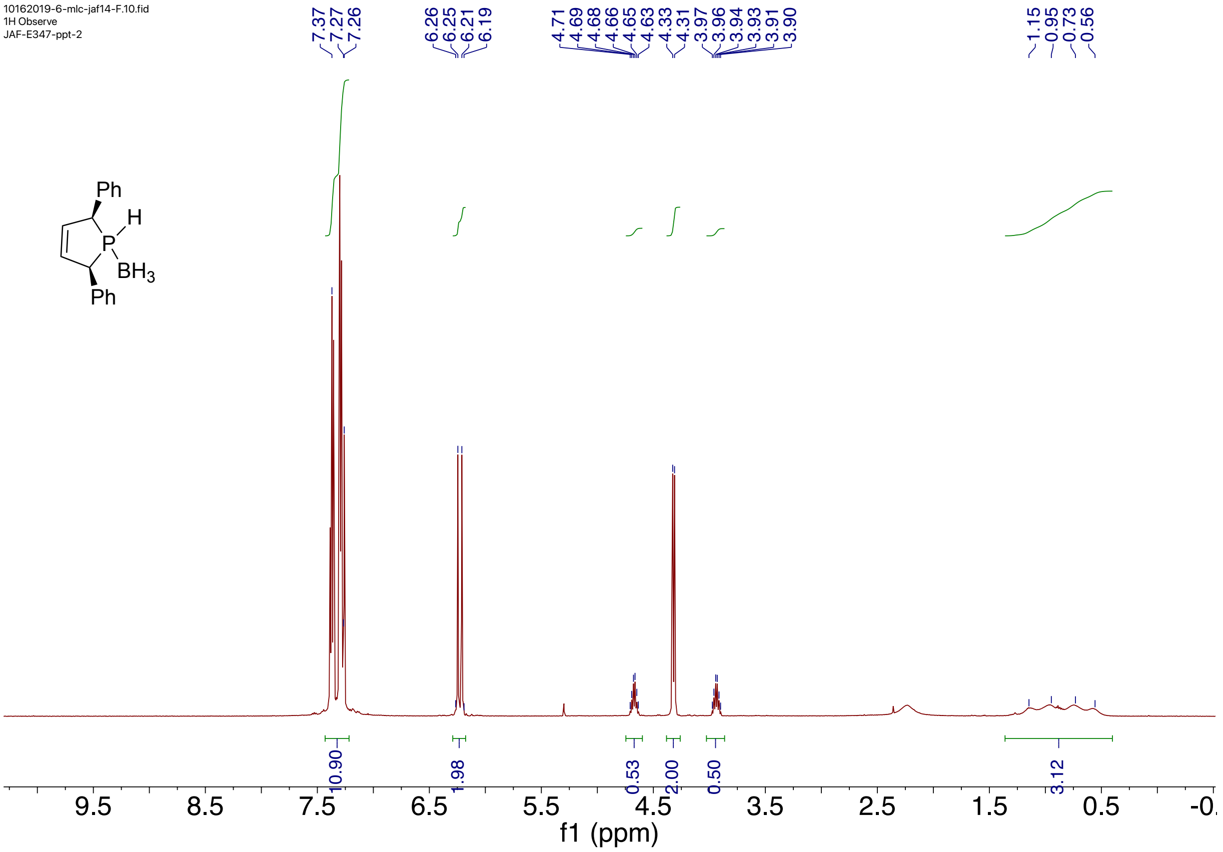

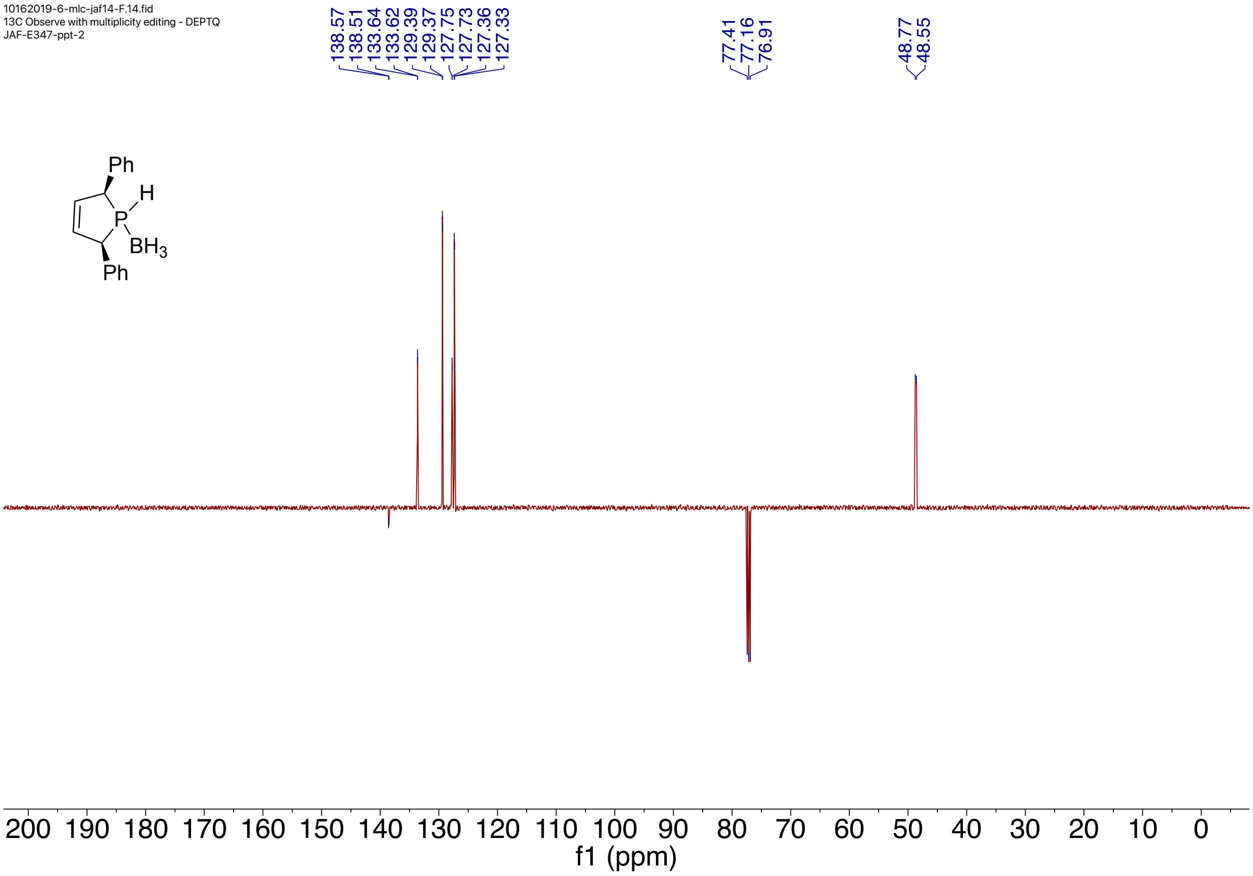

10162019-6-mlc-jaf14-F.11.fid
31P Observe with 1H decoupling
JAF-E347-ppt-2

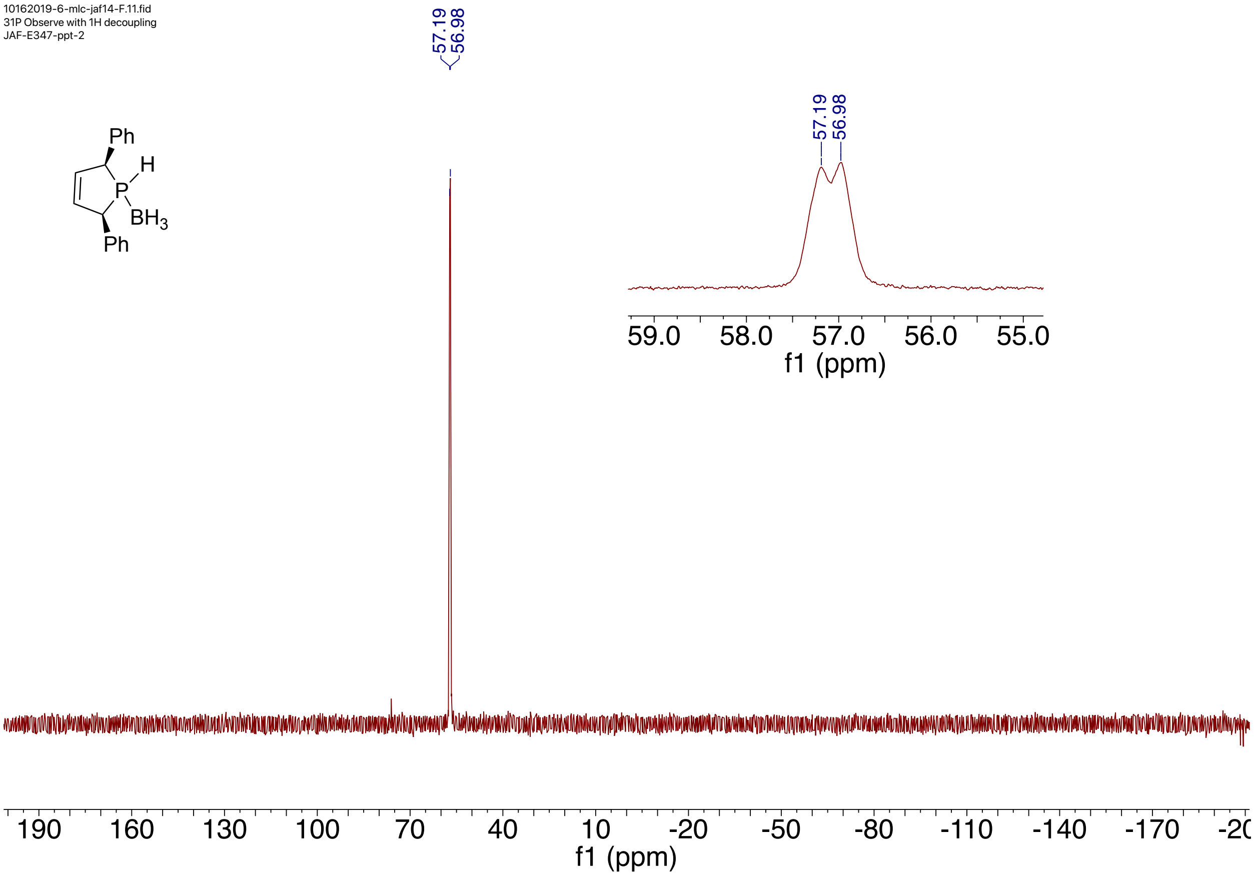

10162019-6-mlc-jaf14-F.12.fid
31P Observe without 1H decoupling
JAF-E347-ppt-2

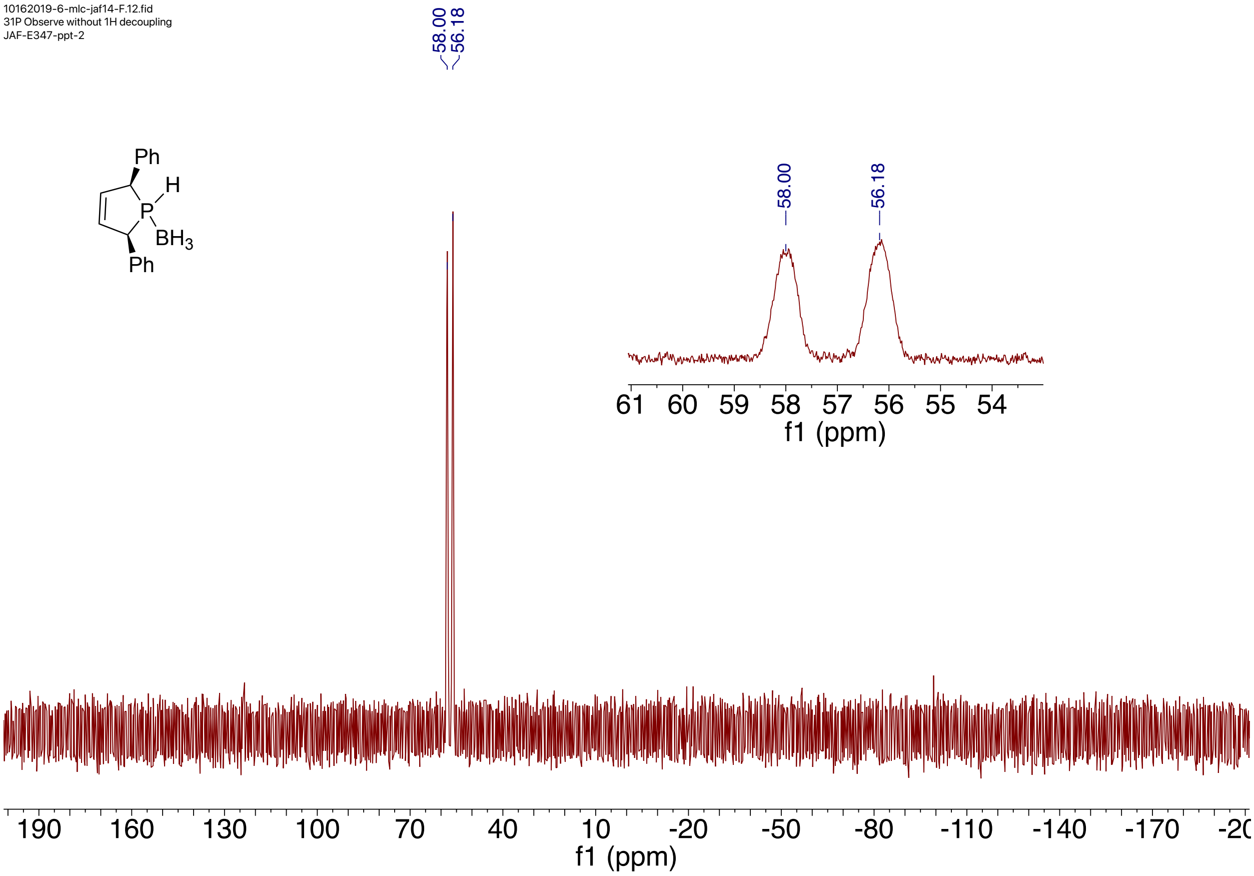

Fig S.10.2. Borane-protected-3-((*meso*)-2,5-diphenyl-2,5-dihydro-1*H*-phosphol-1-yl)-1,1,1-trifluoropropan-2-ol, **10**, ^{1}H NMR (top) ^{13}C NMR (bottom), $^{31}P\{^{1}H\}$NMR (top next page) and ^{19}F NMR (bottom next page).

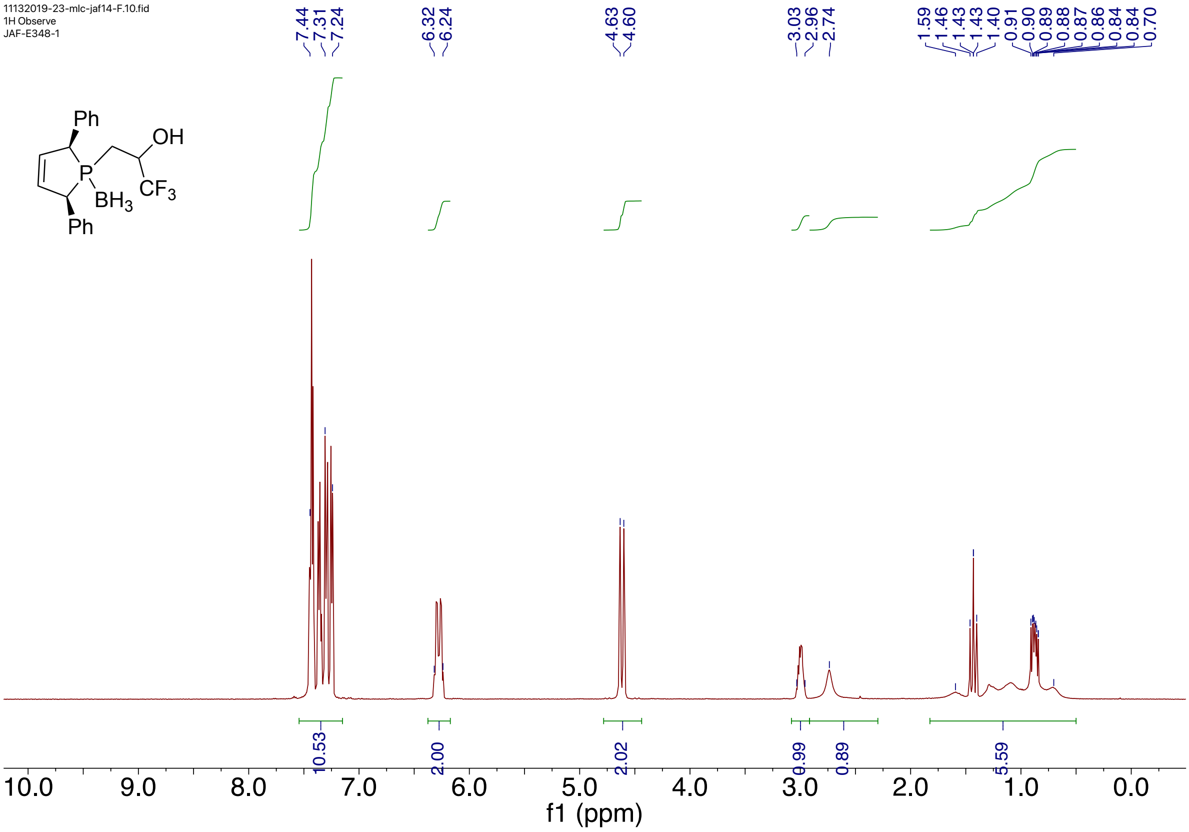

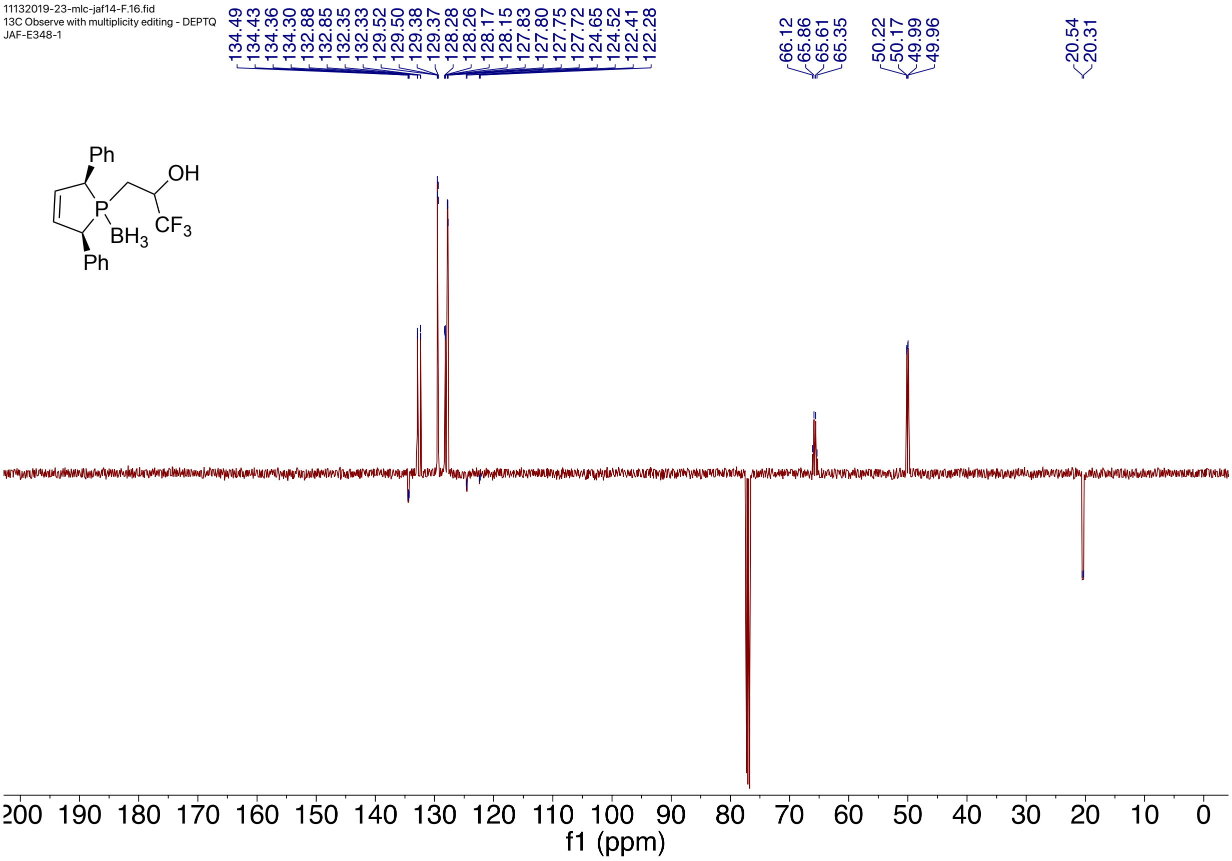

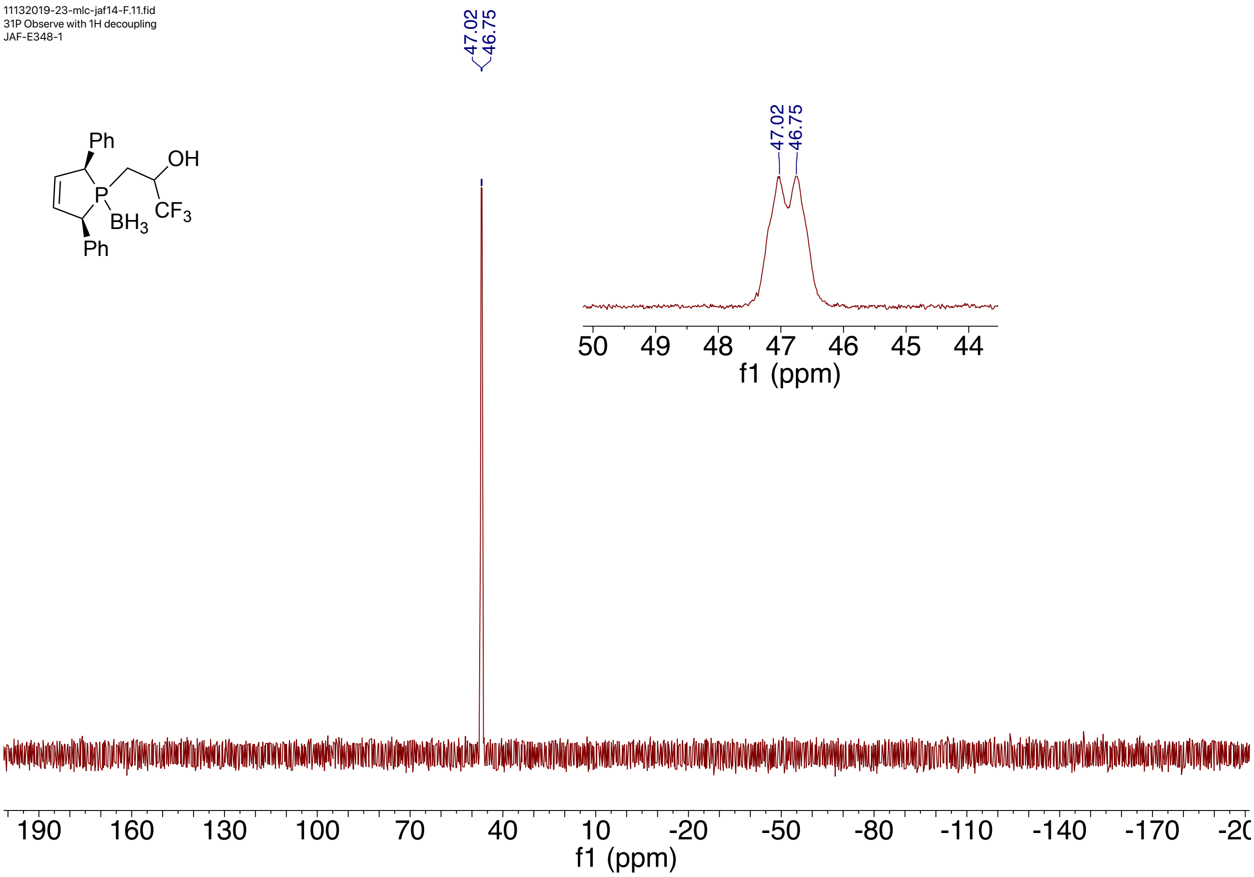
11132019-23-mlc-jaf14-F.11.fid
31P Observe with 1H decoupling
JAF-E348-1
47.02
46.75
Ph
OH
P
BH3
CF3
Ph
50
49
48
47
46
45
44
f1 (ppm)
190
160
130
100
70
40
10
-20
-50
-80
-110
-140
-170
f1 (ppm)

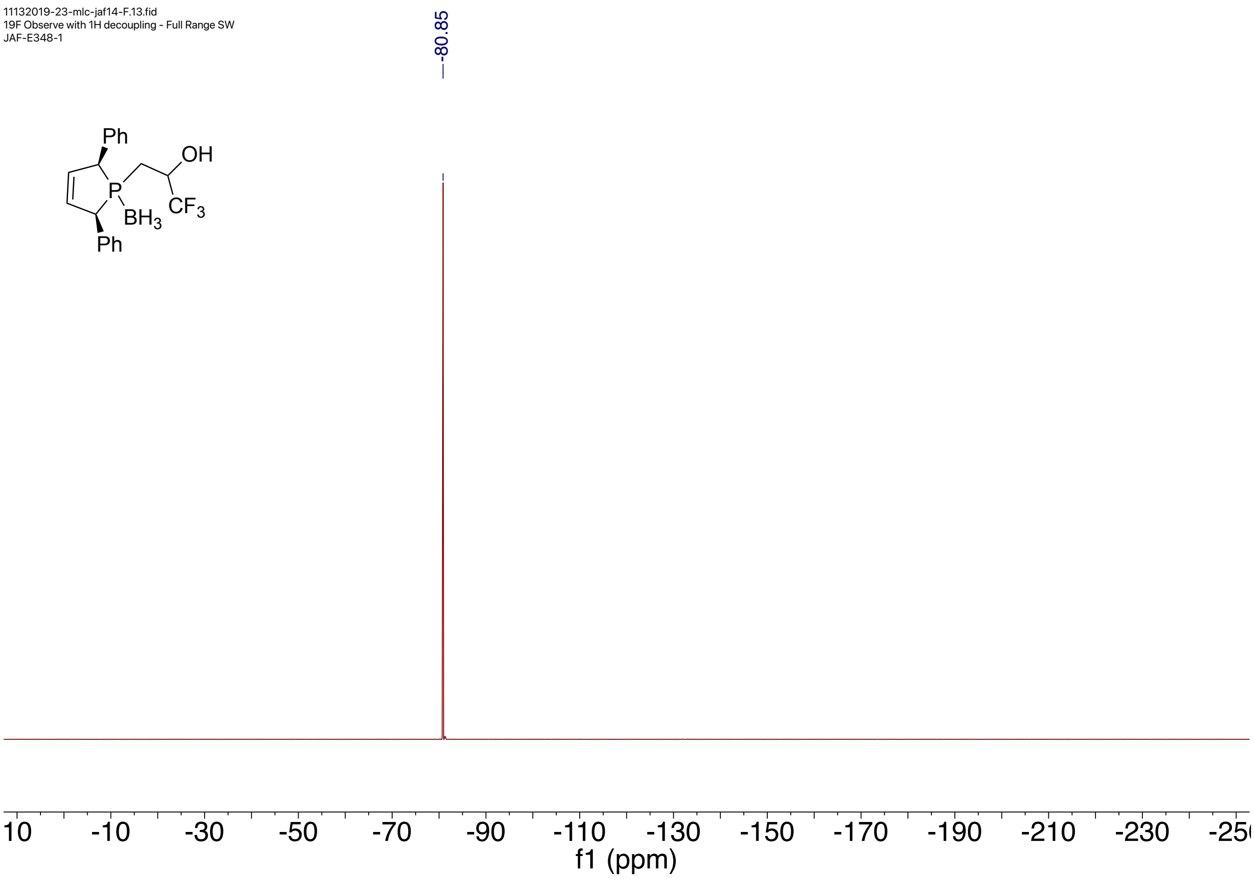
11132019-23-mlc-jaf14-F.13.fid
19F Observe with 1H decoupling - Full Range SW
JAF-E348-1
-80.85
Ph
OH
P
BH3
CF3
Ph
10
-10
-30
-50
-70
-90
-110
-130
-150
-170
-190
-210
-230
f1 (ppm)

Fig S.10.3. 3,3',5,5'-tetrakis(2-phenylpropan-2-yl)-[1,1'-biphenyl]-2,2'-diol, **11b.** ^{1}H NMR (top), ^{13}C NMR (bottom). ^{1}H NMR (acetone-d$_6$) (top next page) and ^{13}C NMR (acetone-d$_6$) (bottom next page).

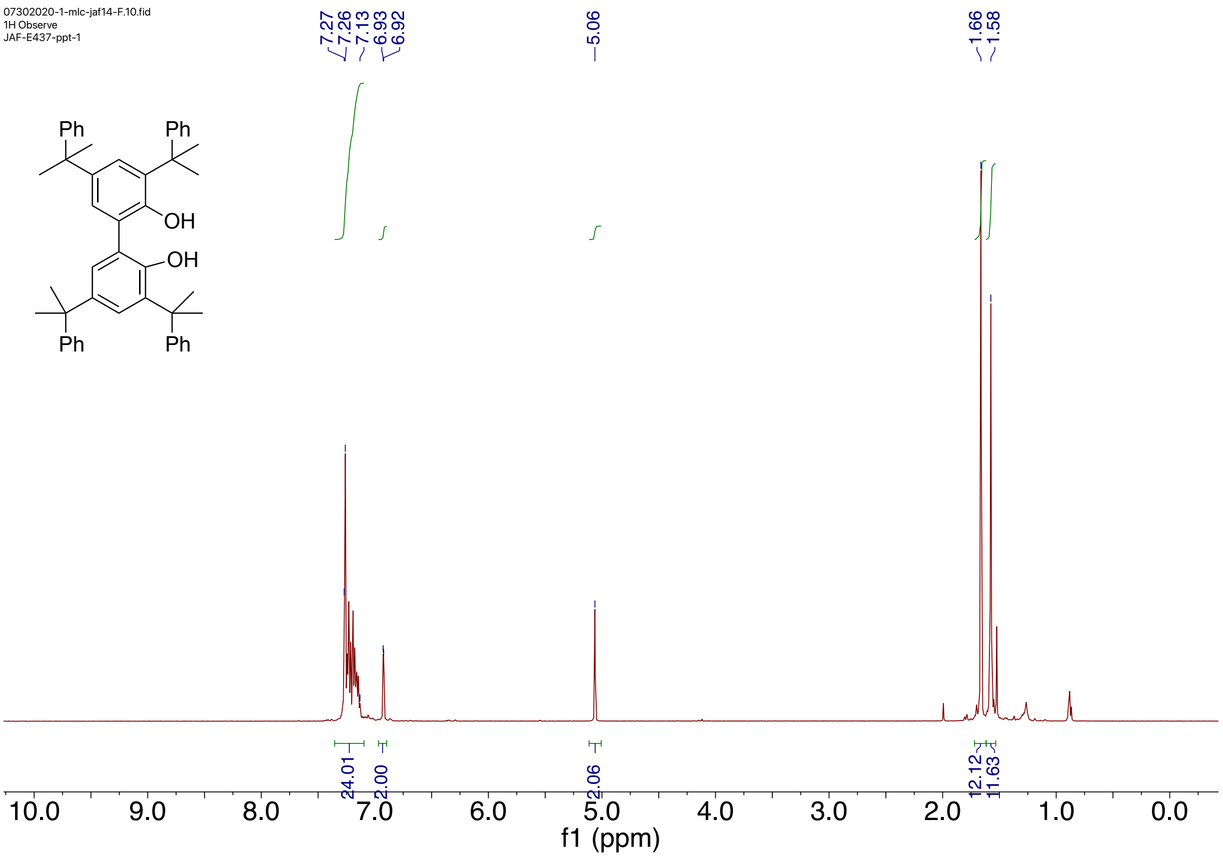

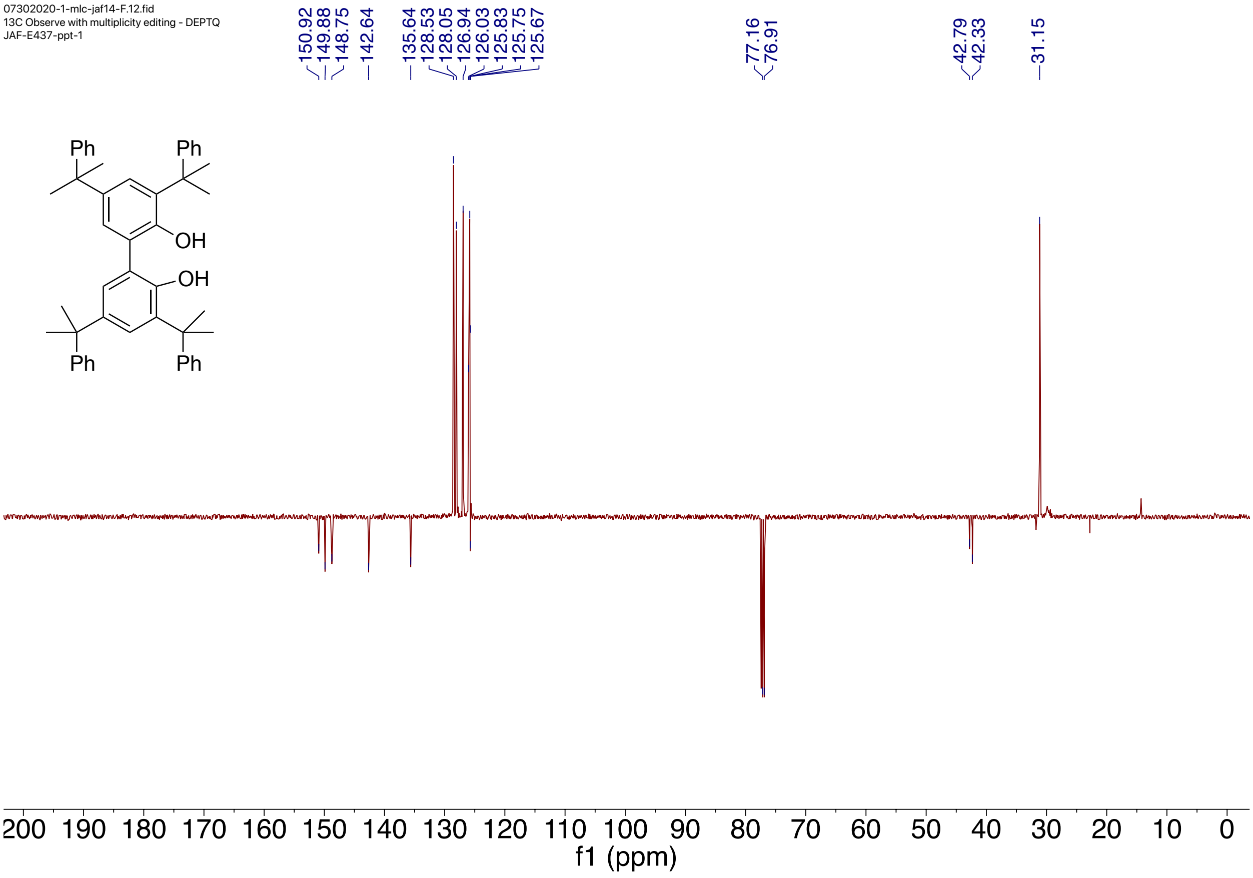

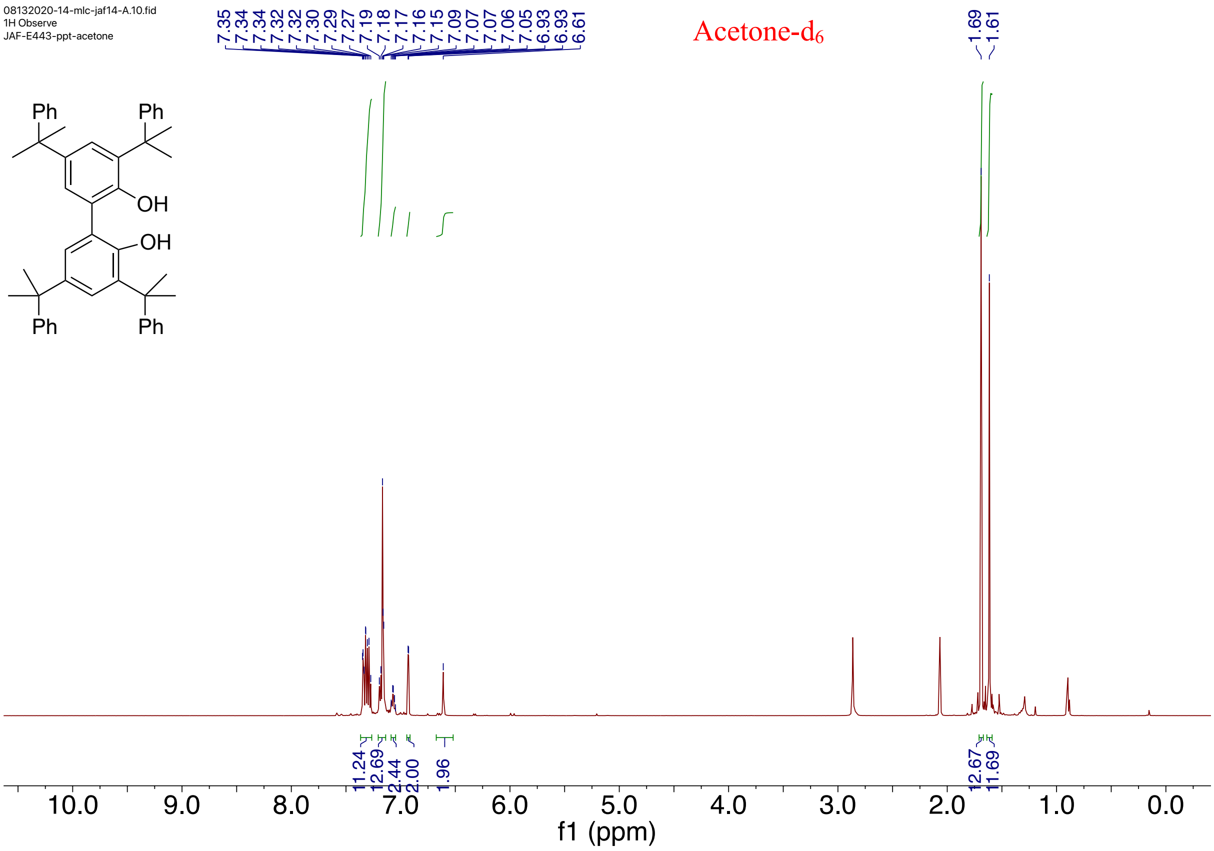
08132020-14-mlc-jaf14-A.10.fid
1H Observe
JAF-E443-ppt-acetone
7.35 7.34 7.34 7.32 7.32 7.30 7.29 7.27 7.19 7.18 7.17 7.16 7.15 7.09 7.07 7.06 7.05 6.93 6.93 6.61
Acetone-d6
1.69 1.61
Ph Ph OH OH Ph Ph
11.24 12.69 2.44 2.00 1.96
12.67 11.69
10.0 9.0 8.0 7.0 6.0 5.0 4.0 3.0 2.0 1.0 0.0
f1 (ppm)

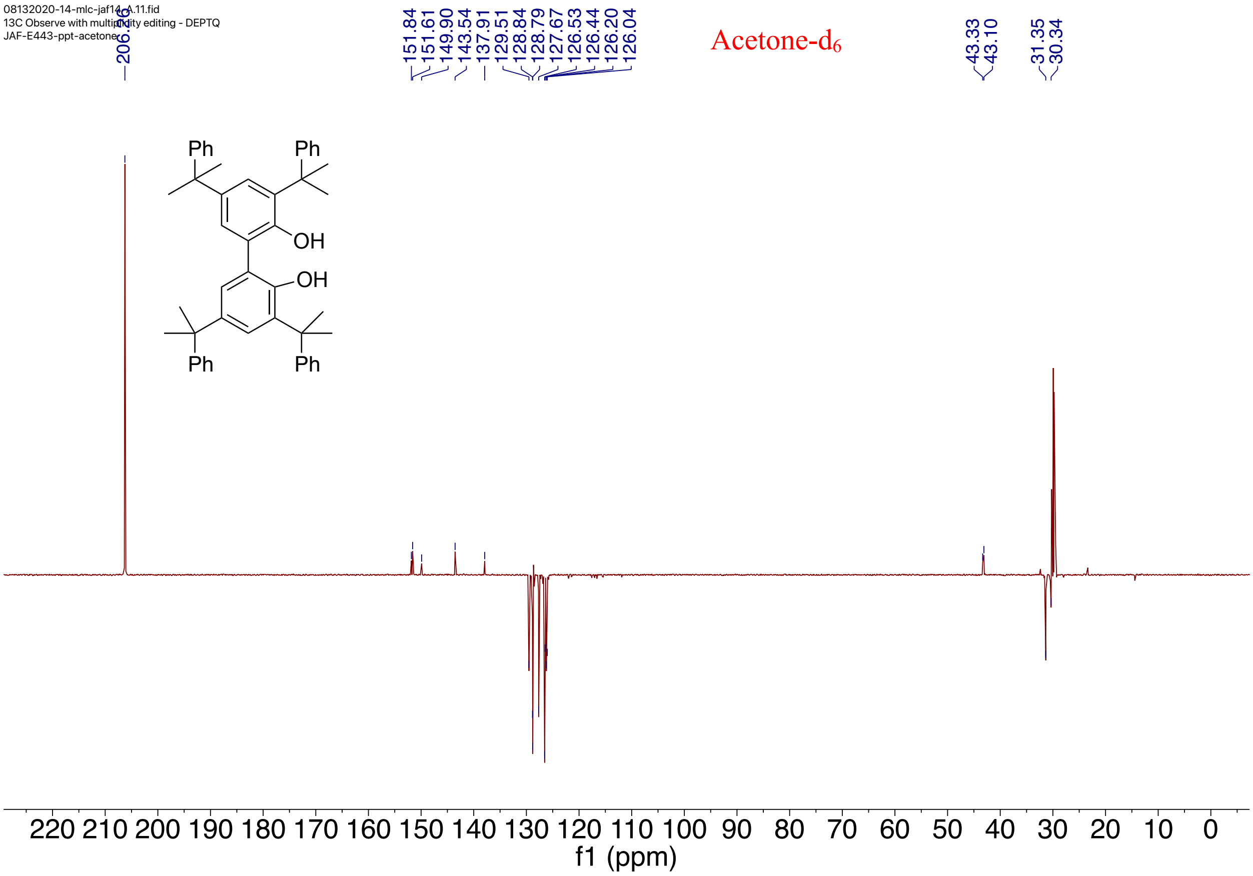
08132020-14-mlc-jaf14-A.11.fid
13C Observe with multiplicity editing - DEPTQ
JAF-E443-ppt-acetone
151.84 151.61 149.90 143.54 137.91 129.51 128.84 128.79 127.67 126.53 126.44 126.20 126.04
Acetone-d6
43.33 43.10
31.35 30.34
Ph Ph OH OH Ph Ph
220 210 200 190 180 170 160 150 140 130 120 110 100 90 80 70 60 50 40 30 20 10 0
f1 (ppm)

Fig S.10.4. 3,3'-di-*tert*-butyl-5,5'-dichloro-[1,1'-biphenyl]-2,2'-diol, **11c.** ^{1}H NMR (top) ^{13}C NMR (bottom).

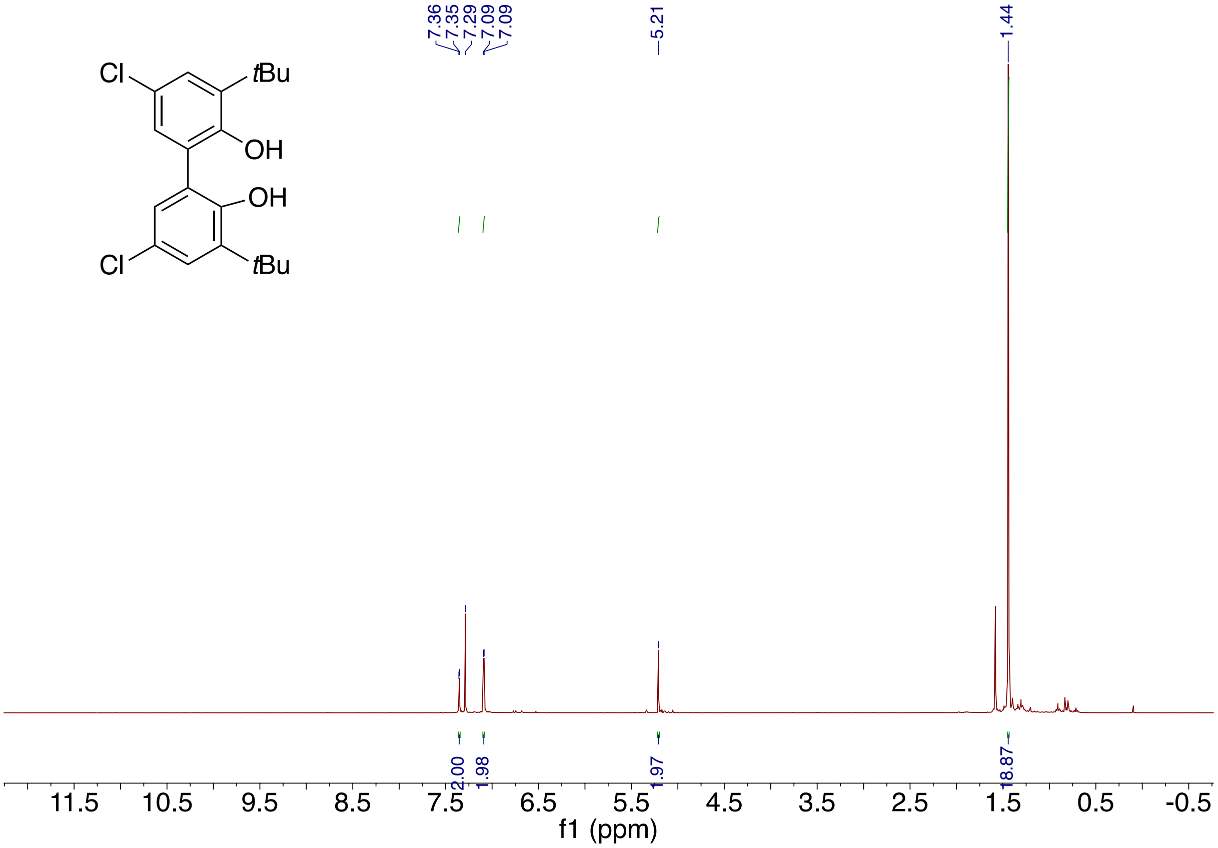

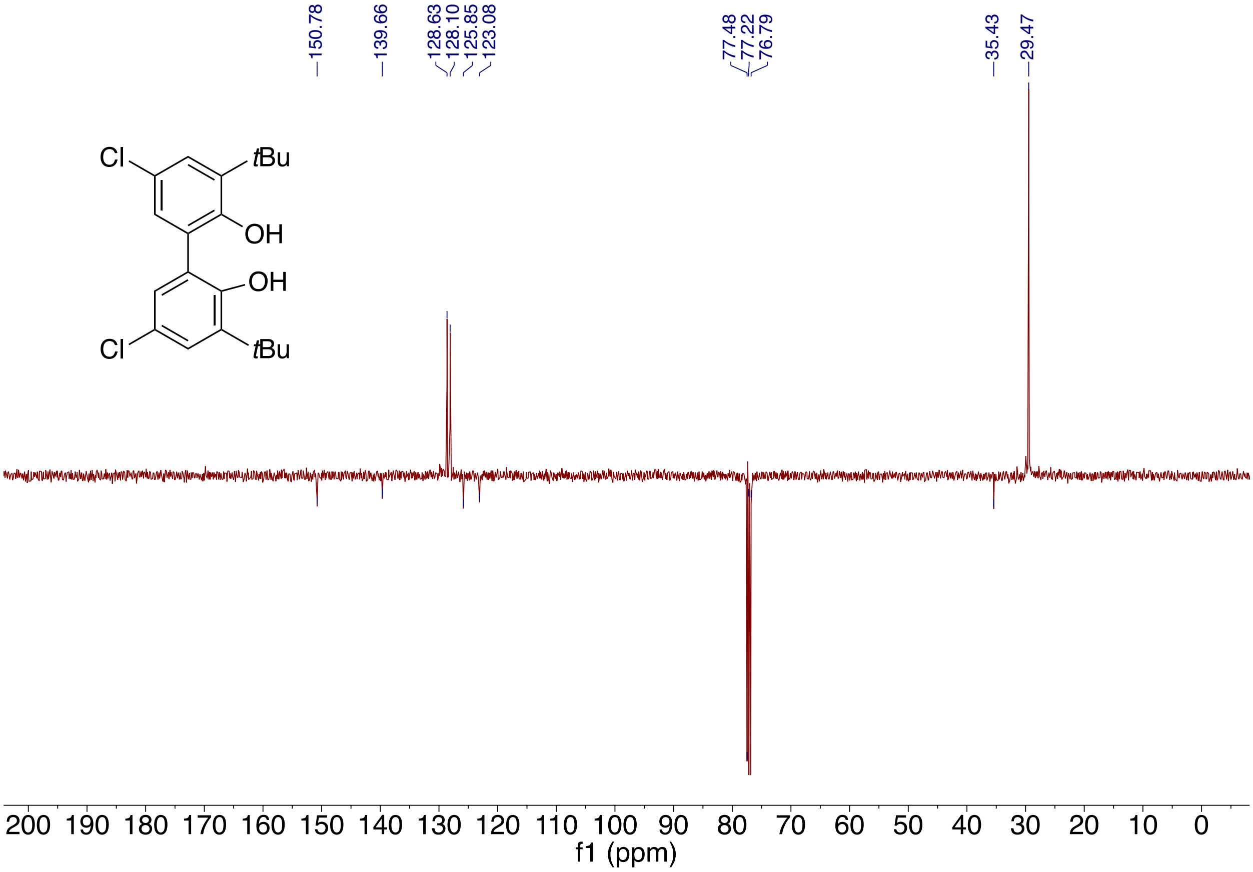

Fig S.10.5. 4,8-di-*tert*-butyl-6-((3-((*meso*)-2,5-diphenyl-2,5-dihydro-1*H*-phosphol-1-yl)-1,1,1-trifluoropropan-2-yl)oxy)-2,10-dimethoxydibenzo[*d*,*f*][1,3,2]dioxaphosphepine, **13a**, ^{1}H NMR (top), ^{13}C NMR (bottom), ^{31}P{^{1}H}NMR (top next page) and ^{19}F NMR (bottom next page).

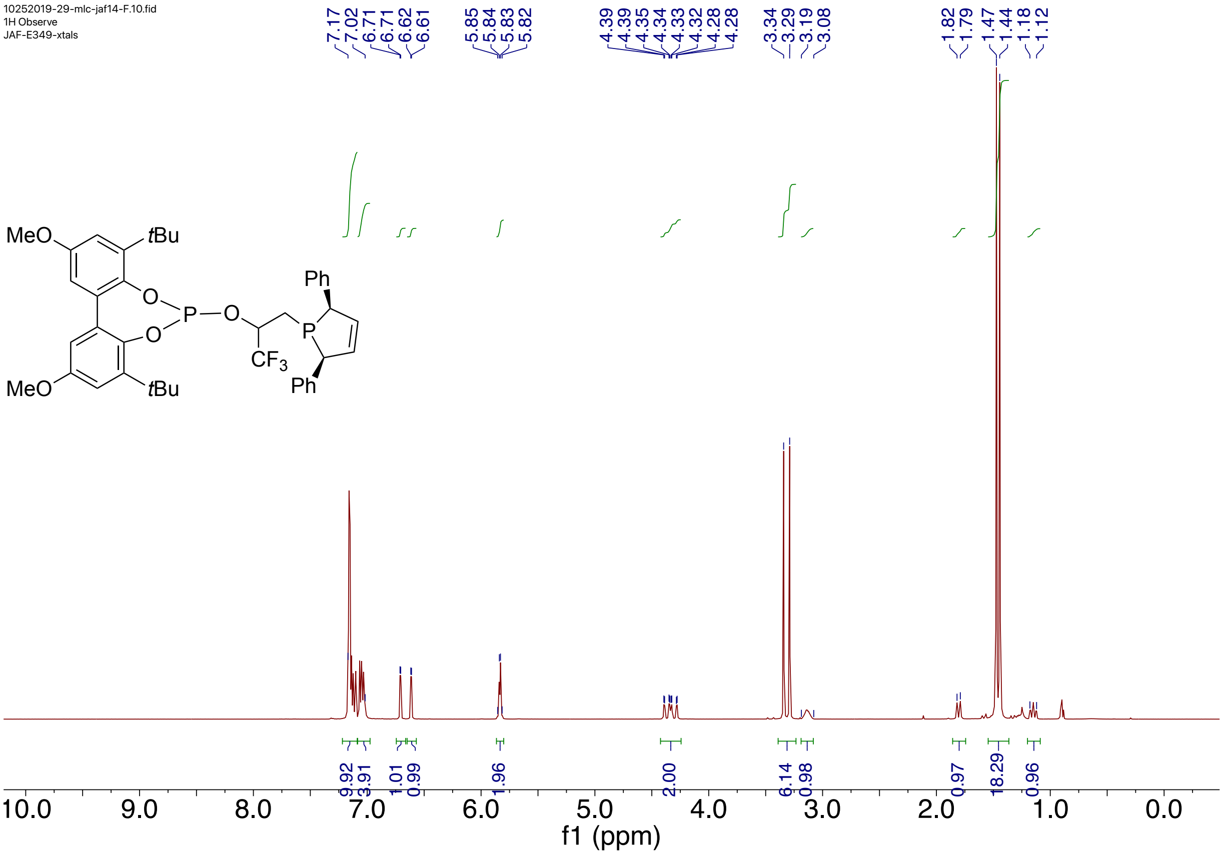

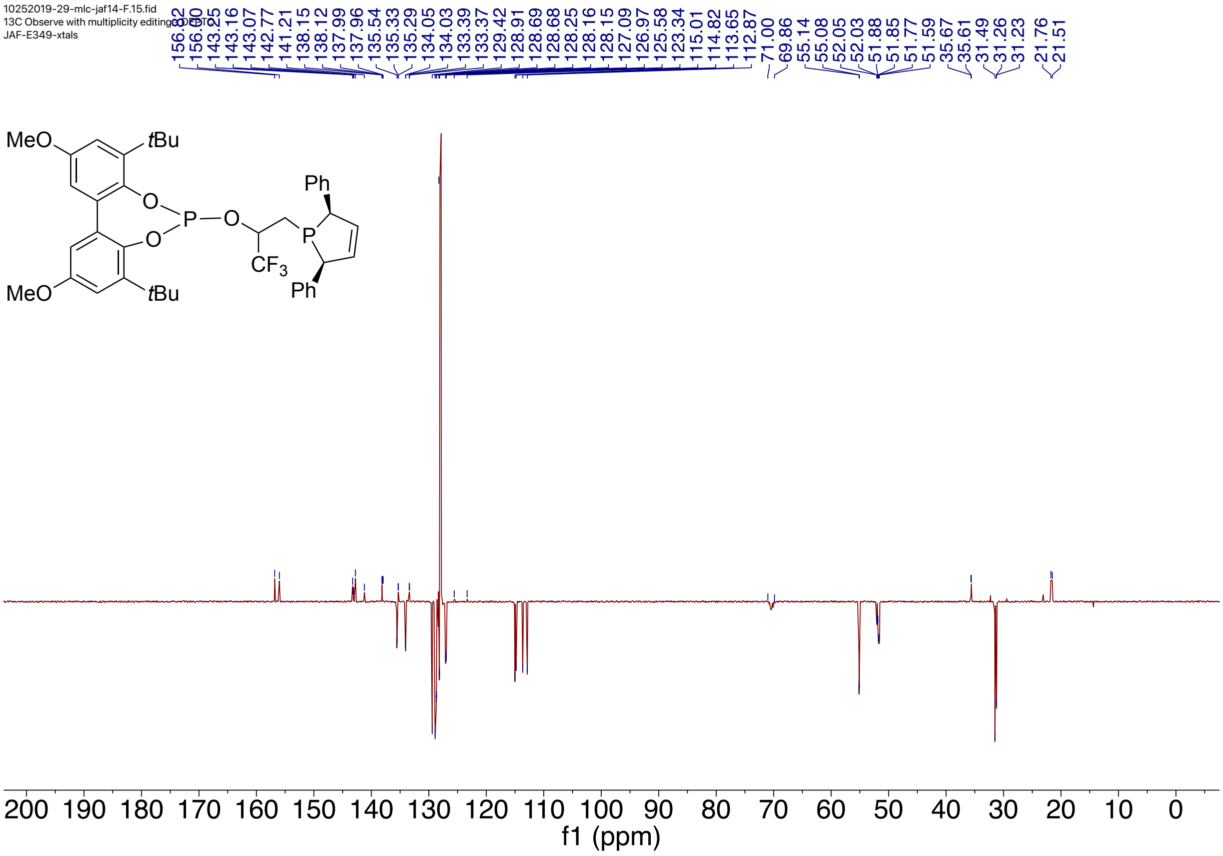

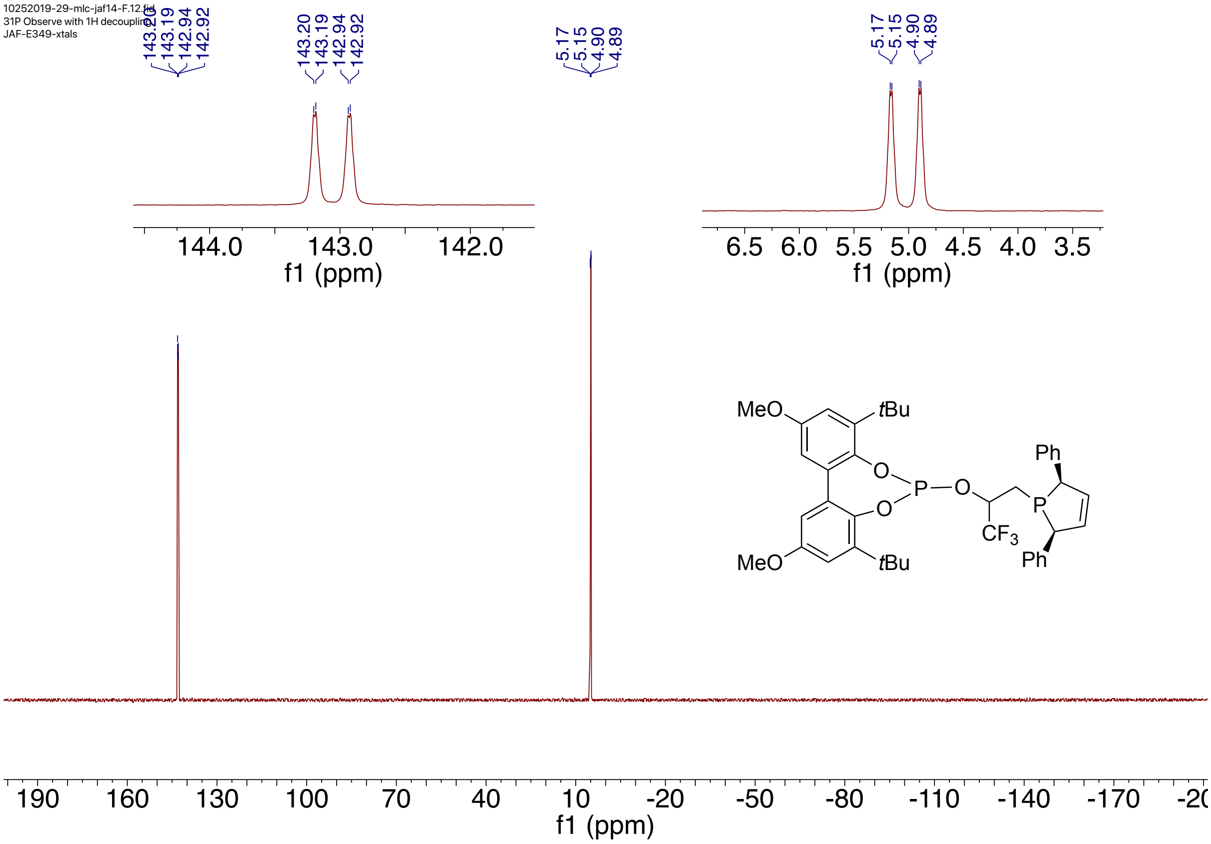

10252019-29-mlc-jaf14-F.12.fid
31P Observe with 1H decoupling
JAF-E349-xtals
143.20
143.19
142.94
142.92
5.17
5.15
4.90
4.89
144.0 143.0 142.0
f1 (ppm)
6.5 6.0 5.5 5.0 4.5 4.0 3.5
f1 (ppm)
MeO
tBu
O
P
O
CF3
Ph
MeO
tBu
190 160 130 100 70 40 10 -20 -50 -80 -110 -140 -170
f1 (ppm)

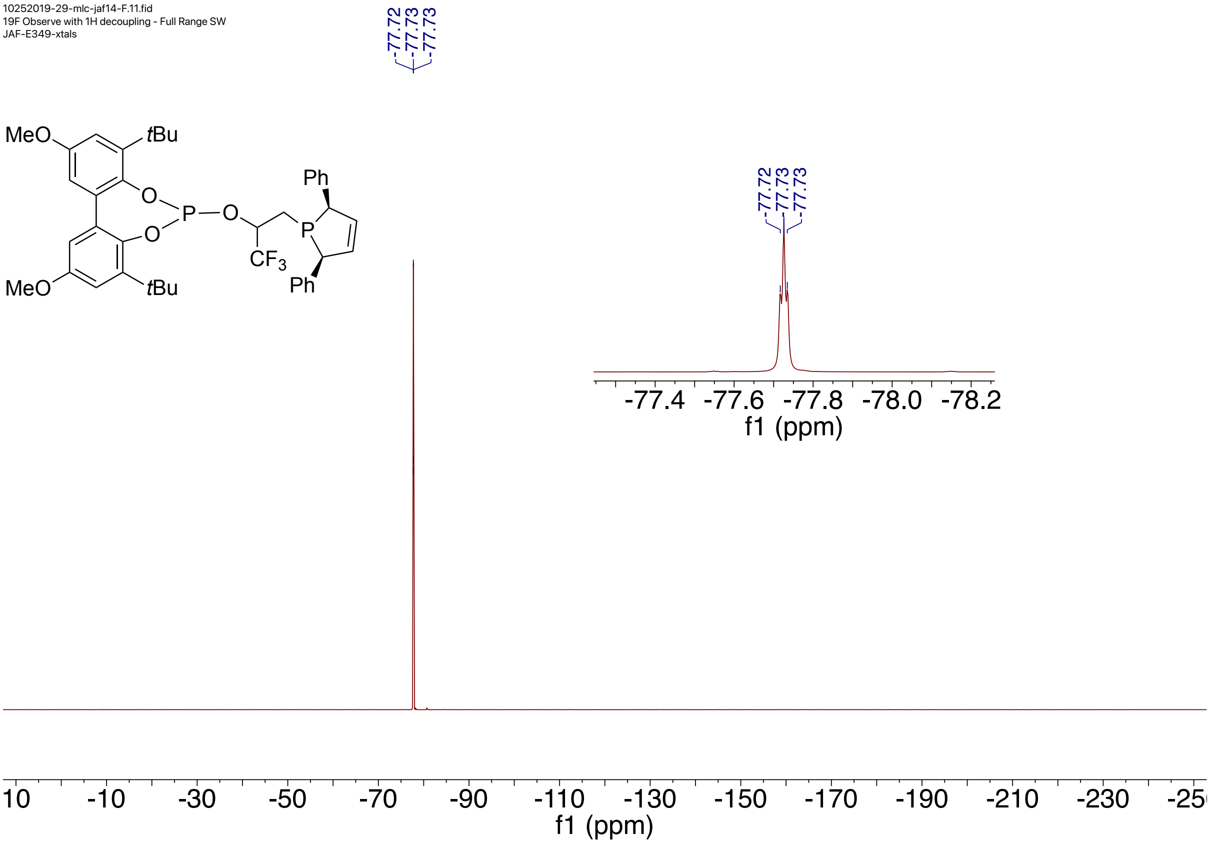

10252019-29-mlc-jaf14-F.11.fid
19F Observe with 1H decoupling - Full Range SW
JAF-E349-xtals
-77.72
-77.73
-77.73
MeO
tBu
O
P
O
CF3
Ph
MeO
tBu
-77.4 -77.6 -77.8 -78.0 -78.2
f1 (ppm)
10 -10 -30 -50 -70 -90 -110 -130 -150 -170 -190 -210 -230
f1 (ppm)

Fig S.10.6. 6-((3-((*meso*)-2,5-diphenyl-2,5-dihydro-1*H*-phosphol-1-yl)-1,1,1-trifluoropropan-2-yl)oxy)-2,4,8,10-tetrakis(2-phenylpropan-2-yl)dibenzo[*d*,*f*][1,3,2]dioxaphosphepine, **13b**. ^{1}H NMR (top), ^{13}C NMR (bottom), ^{31}P{^{1}H}NMR (top next page) and ^{19}F NMR (bottom next page).

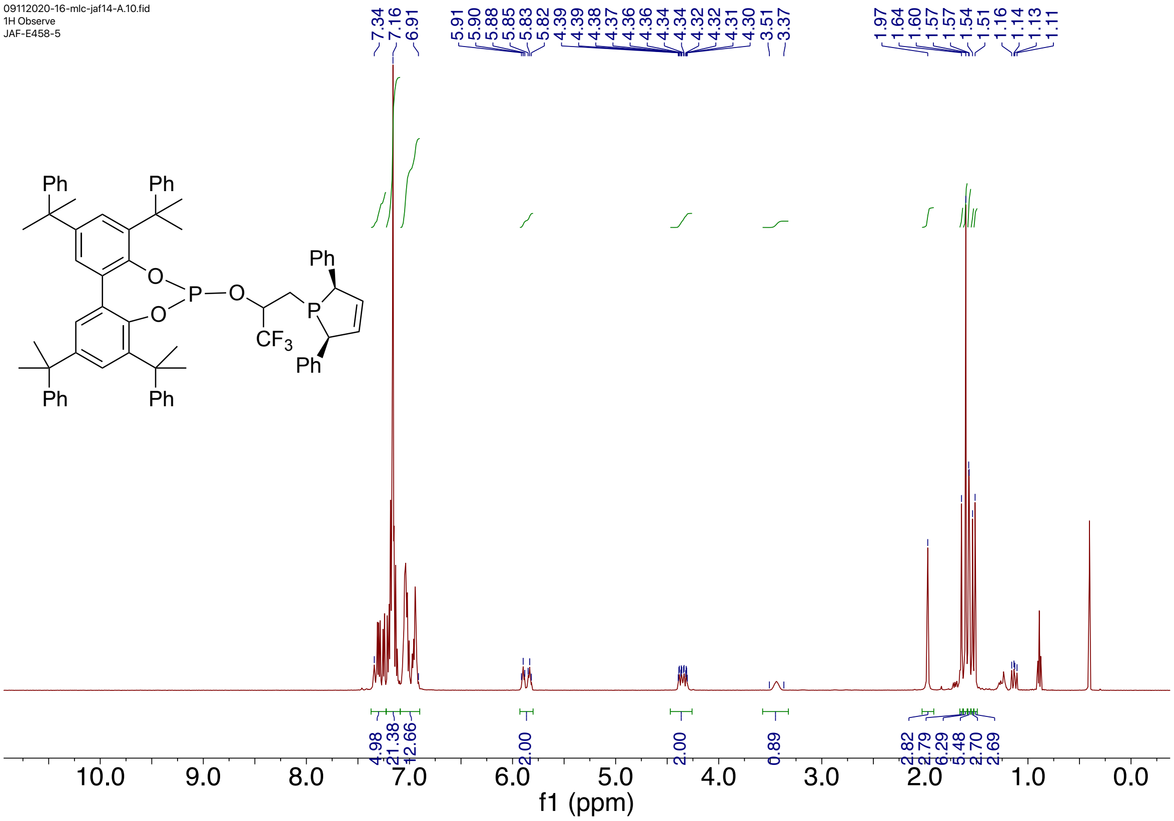

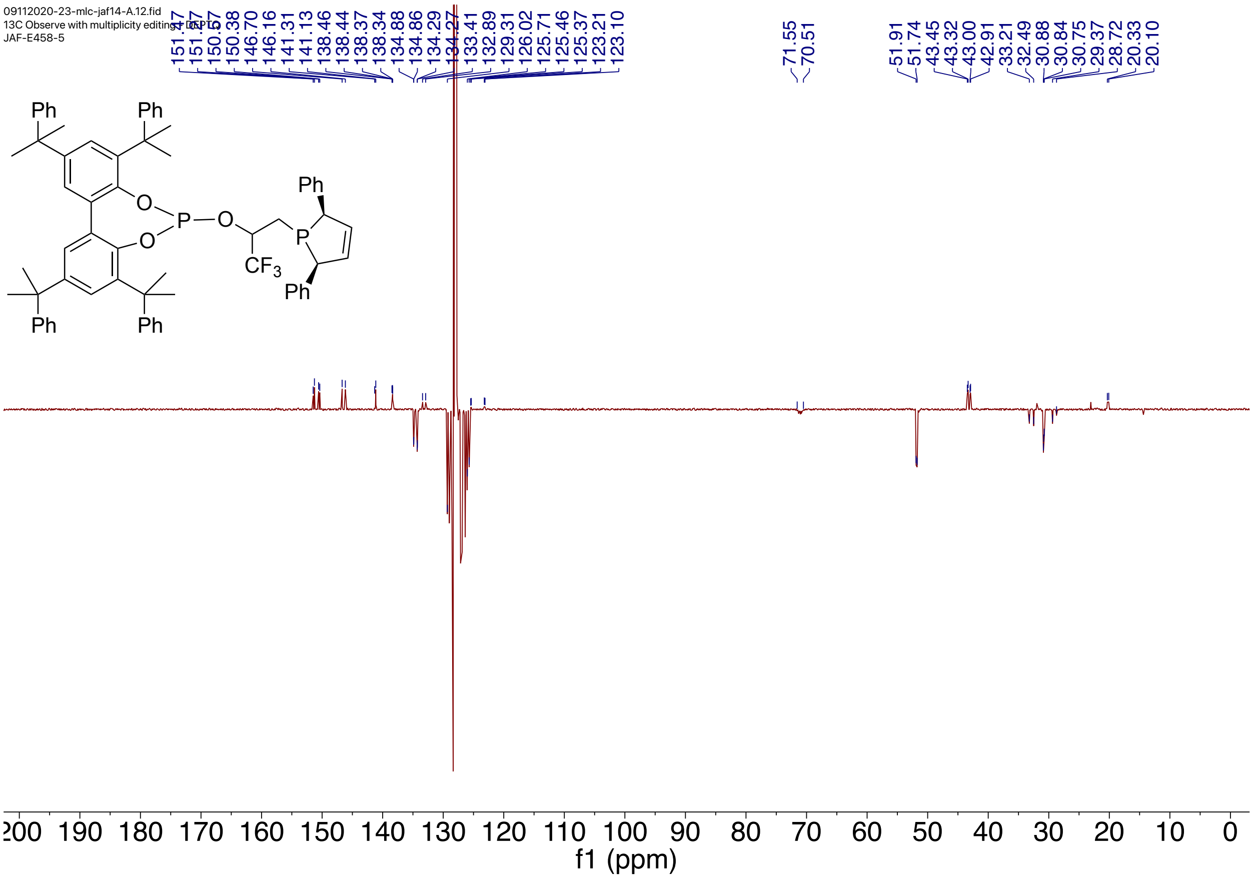

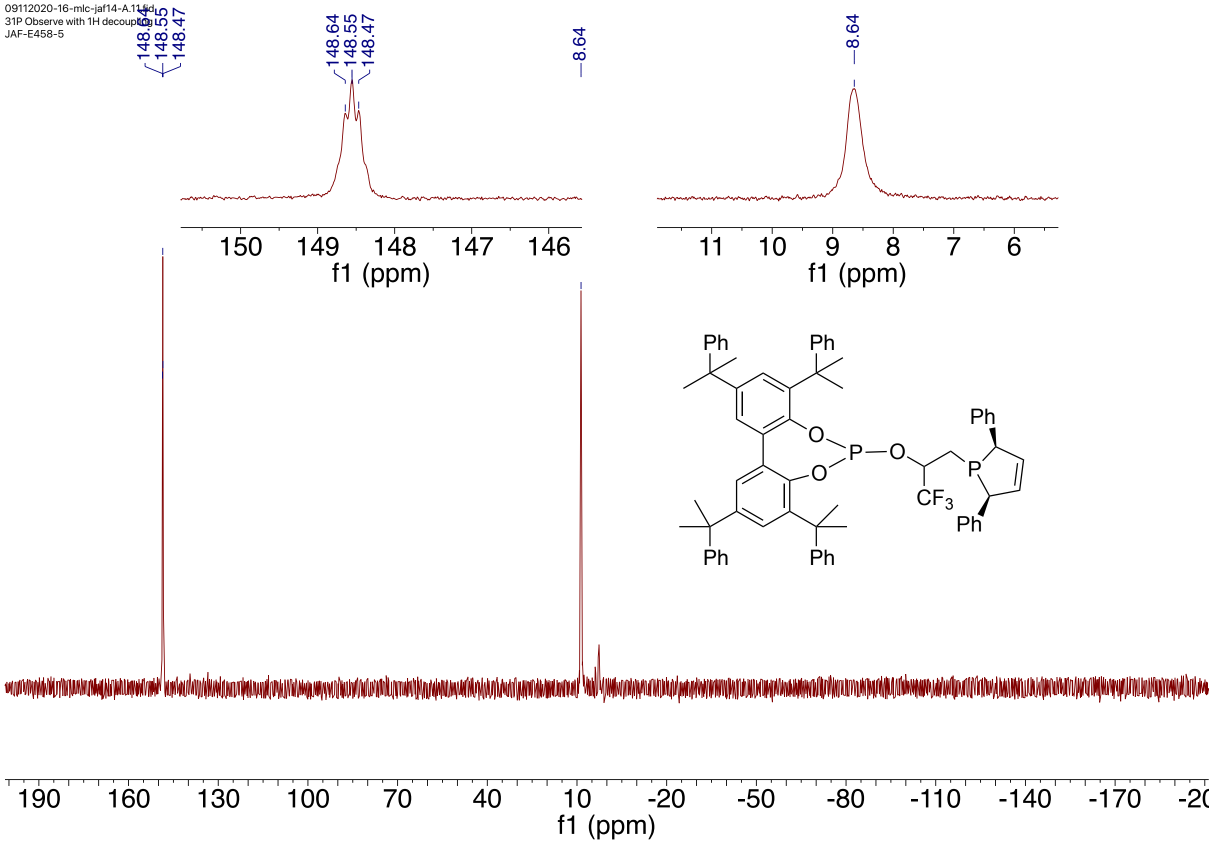
09112020-16-mlc-jaf14-A.11.fid
31P Observe with 1H decoupling
JAF-E458-5
148.64
148.55
148.47
8.64
150
149
148
147
146
f1 (ppm)
11
10
9
8
7
6
f1 (ppm)
Ph
O
P
O
CF3
190
160
130
100
70
40
10
-20
-50
-80
-110
-140
-170
f1 (ppm)

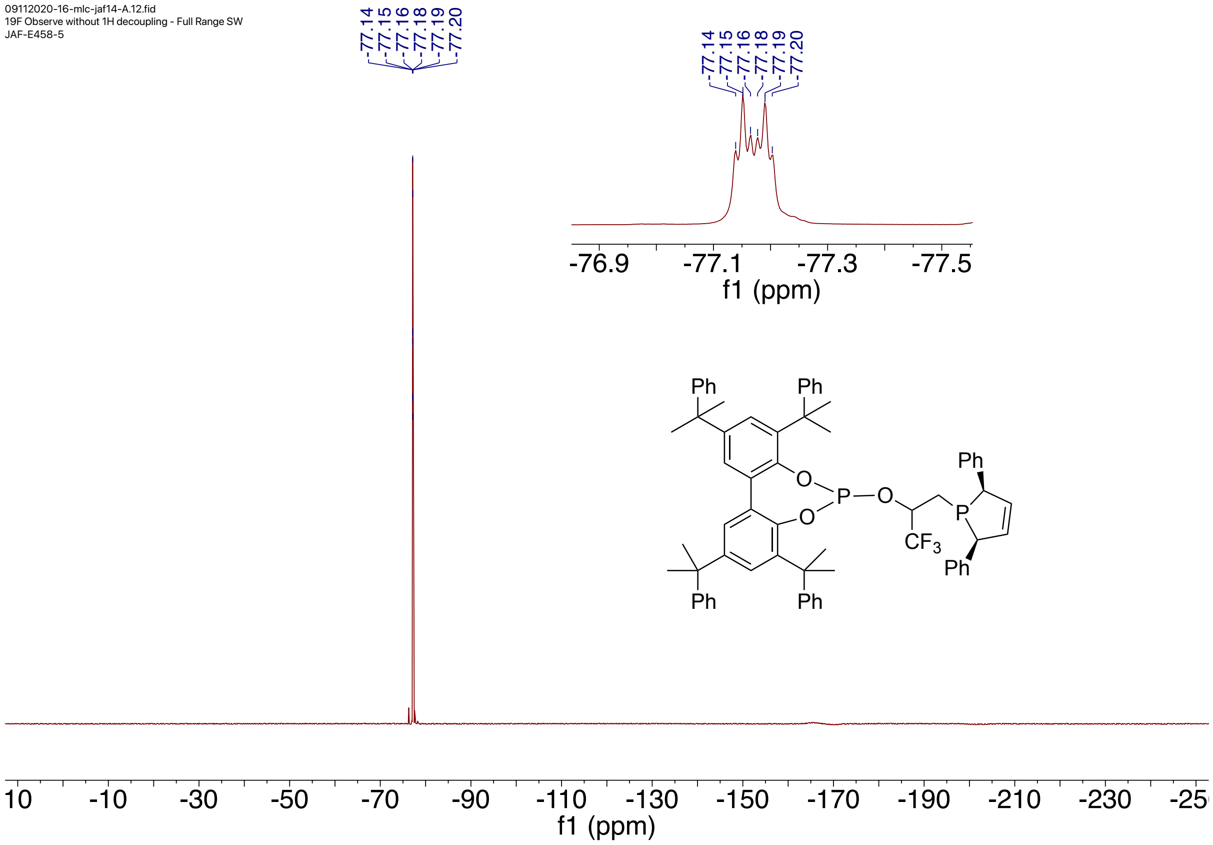
09112020-16-mlc-jaf14-A.12.fid
19F Observe without 1H decoupling - Full Range SW
JAF-E458-5
-77.14
-77.15
-77.16
-77.18
-77.19
-77.20
-76.9
-77.1
-77.3
-77.5
f1 (ppm)
Ph
O
P
O
CF3
10
-10
-30
-50
-70
-90
-110
-130
-150
-170
-190
-210
-230
f1 (ppm)

Fig S.10.7. 4,8-di-*tert*-butyl-2,10-dichloro-6-((3-((2*R*,5*S*)-2,5-diphenyl-2,5-dihydro-1*H*-phosphol-1-yl)-1,1,1-trifluoropropan-2-yl)oxy)dibenzo[*d*,*f*][1,3,2]dioxaphosphepine, **13c**. ^{1}H NMR (top) ^{13}C NMR (bottom), ^{31}P{^{1}H}NMR (top next page) and ^{19}F NMR (bottom next page).

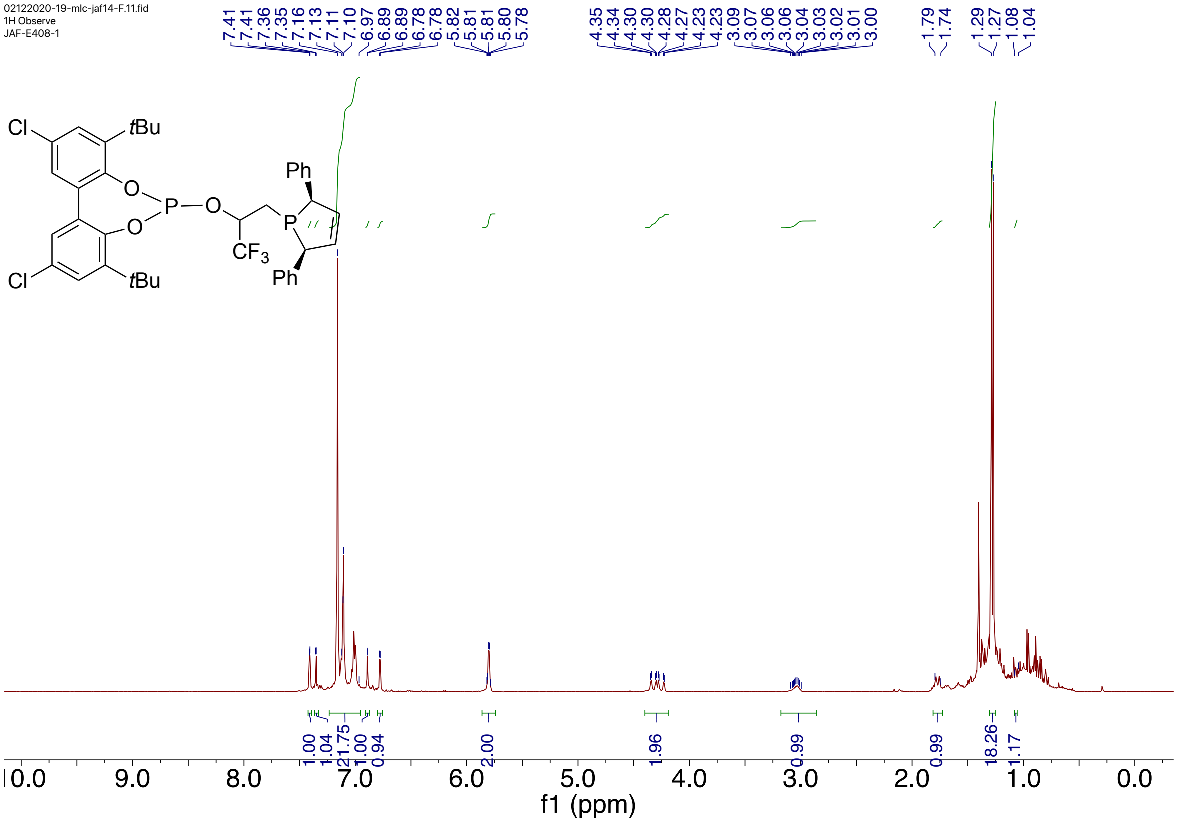

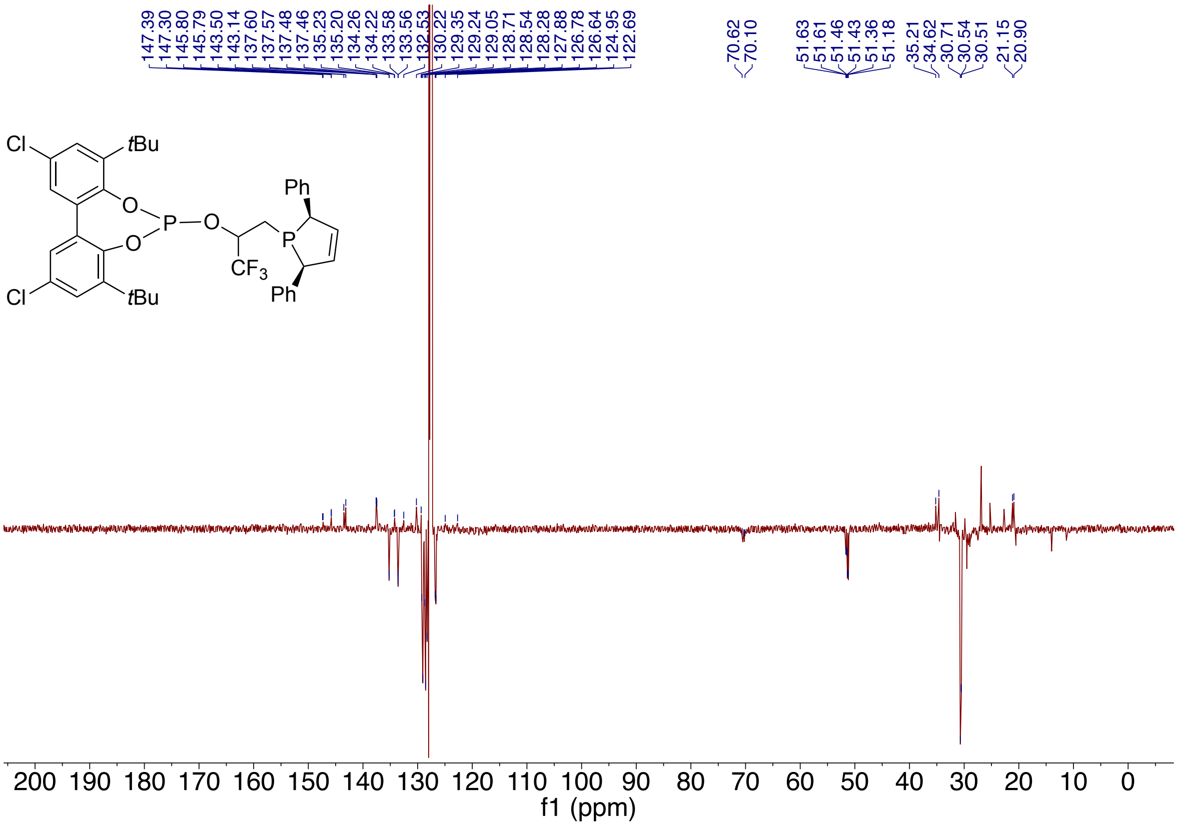

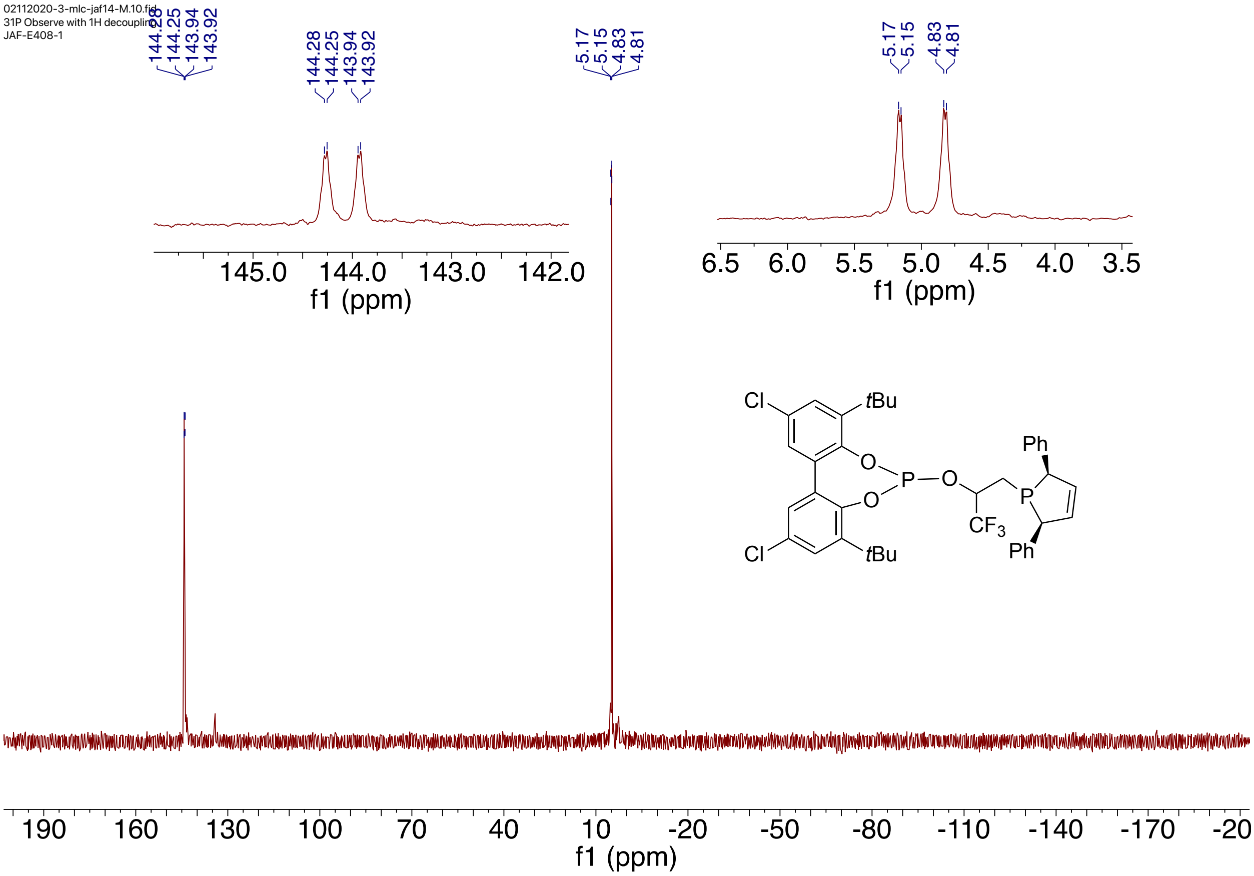

02112020-3-mlc-jaf14-M.10.fid
31P Observe with 1H decoupling
JAF-E408-1
144.28
144.25
143.94
143.92
5.17
5.15
4.83
4.81
145.0
144.0
143.0
142.0
f1 (ppm)
6.5
6.0
5.5
5.0
4.5
4.0
3.5
f1 (ppm)
Cl
tBu
O
P
O
Cl
tBu
CF3
P
Ph
Ph
190
160
130
100
70
40
10
-20
-50
-80
-110
-140
-170
f1 (ppm)

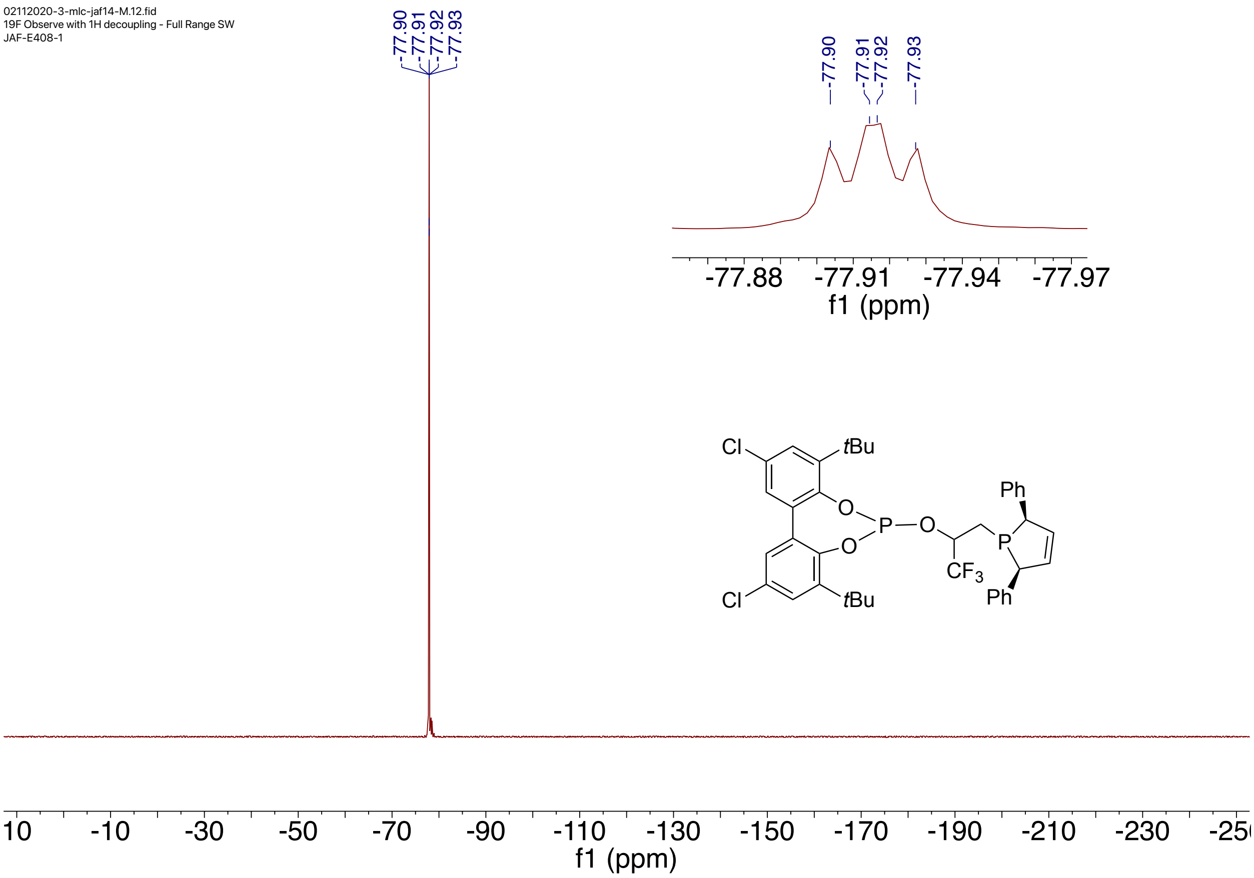

02112020-3-mlc-jaf14-M.12.fid
19F Observe with 1H decoupling - Full Range SW
JAF-E408-1
-77.90
-77.91
-77.92
-77.93
-77.88
-77.91
-77.94
-77.97
f1 (ppm)
Cl
tBu
O
P
O
Cl
tBu
CF3
P
Ph
Ph
10
-10
-30
-50
-70
-90
-110
-130
-150
-170
-190
-210
-230
f1 (ppm)

Fig S.10.8. [$PdCl_2$(**13a**)], complex **14**.

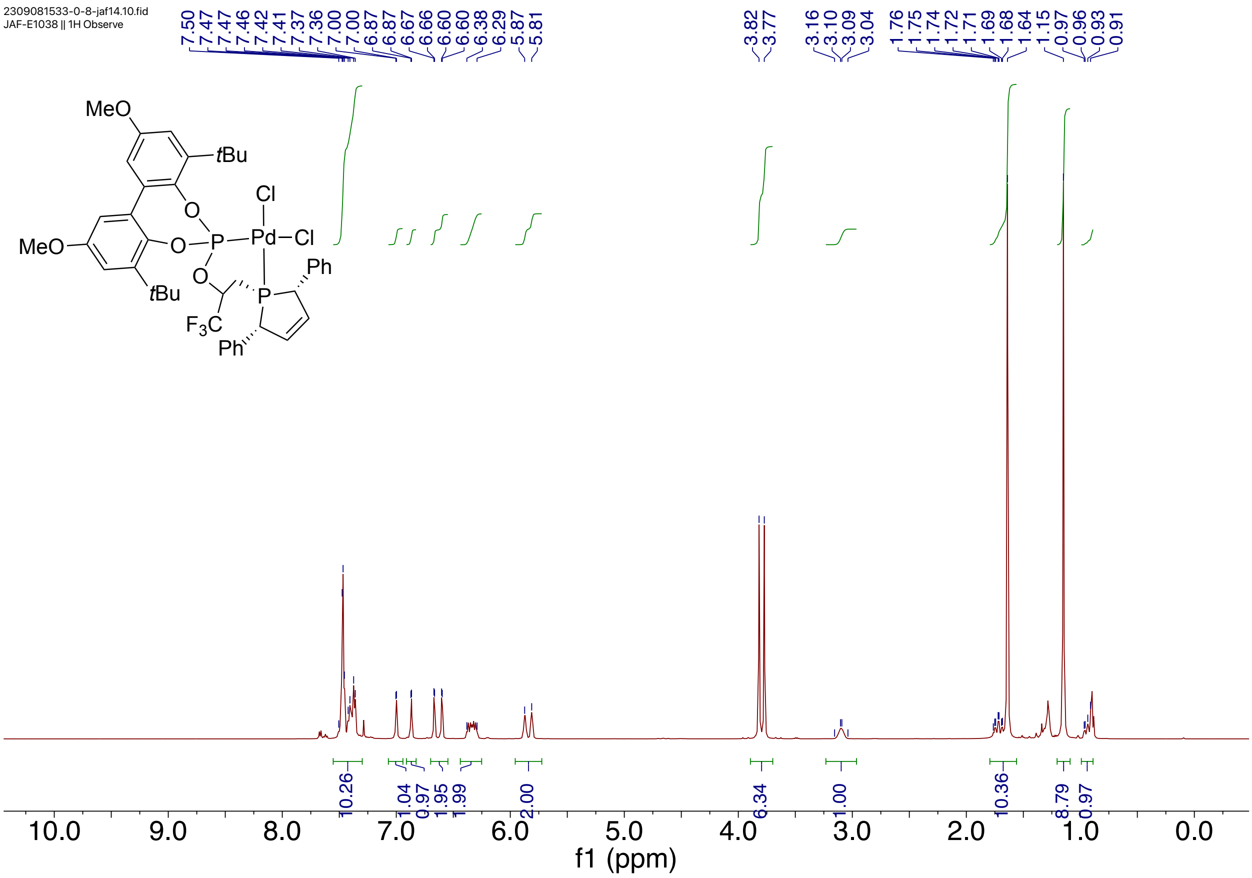

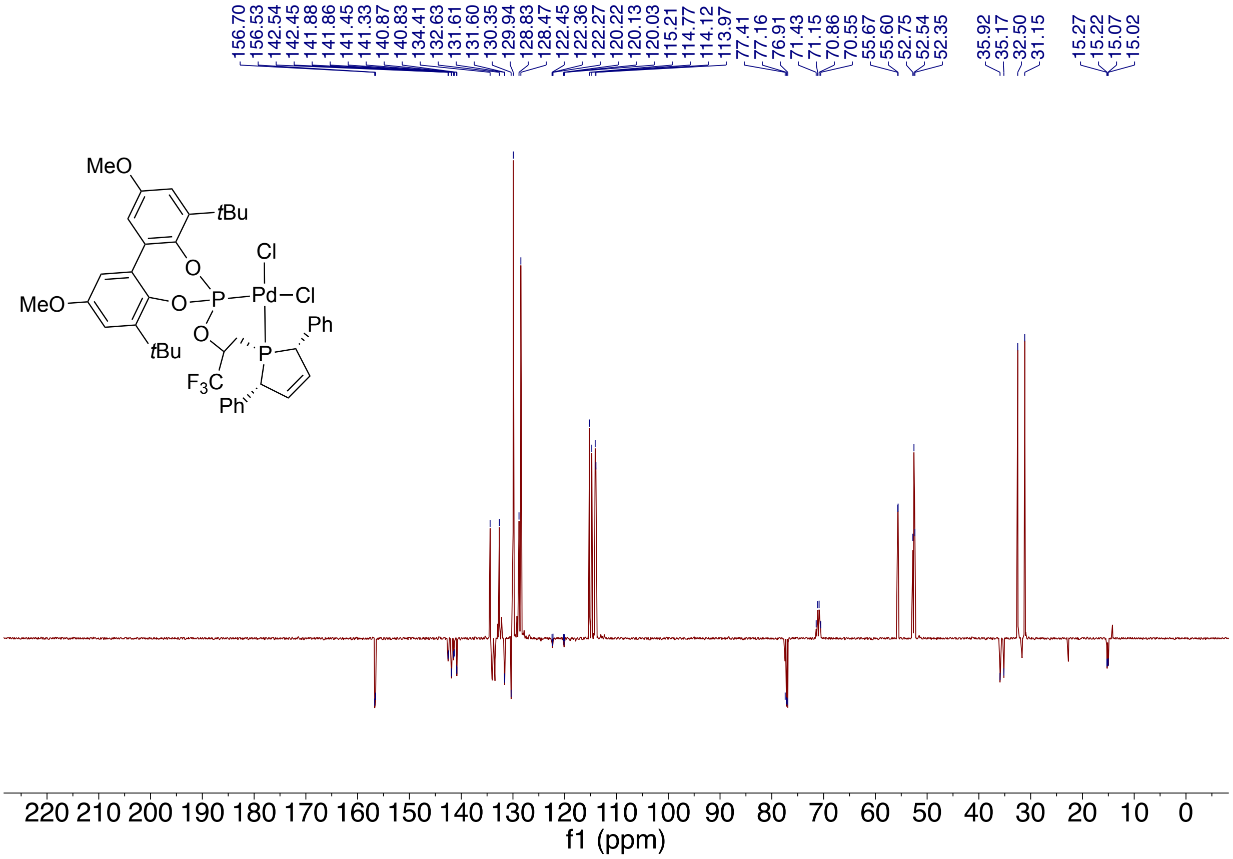

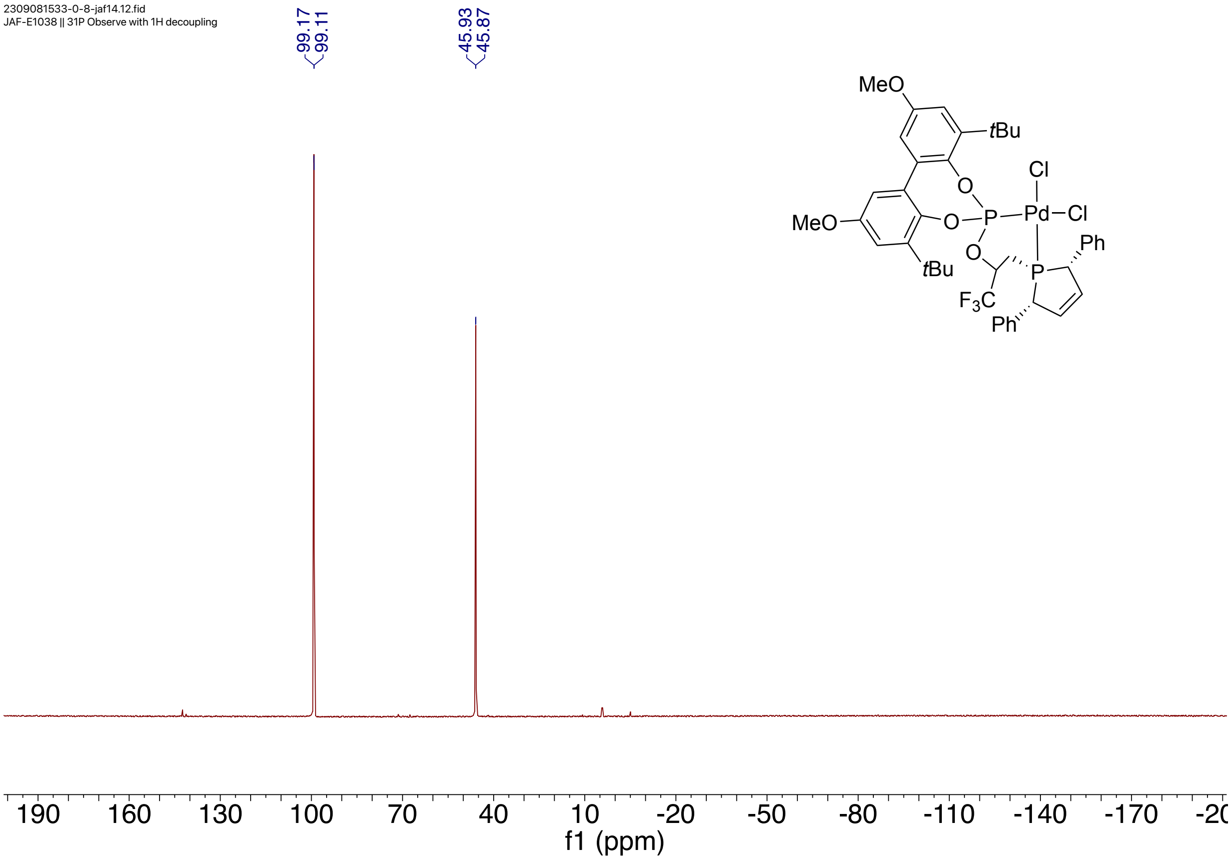
2309081533-0-8-jaf14.12.fid
JAF-E1038 || 31P Observe with 1H decoupling
99.17
99.11
45.93
45.87
MeO
tBu
Cl
O
O–P–Pd–Cl
MeO
O
Ph
tBu
P
F3C
Ph
190 160 130 100 70 40 10 -20 -50 -80 -110 -140 -170
f1 (ppm)

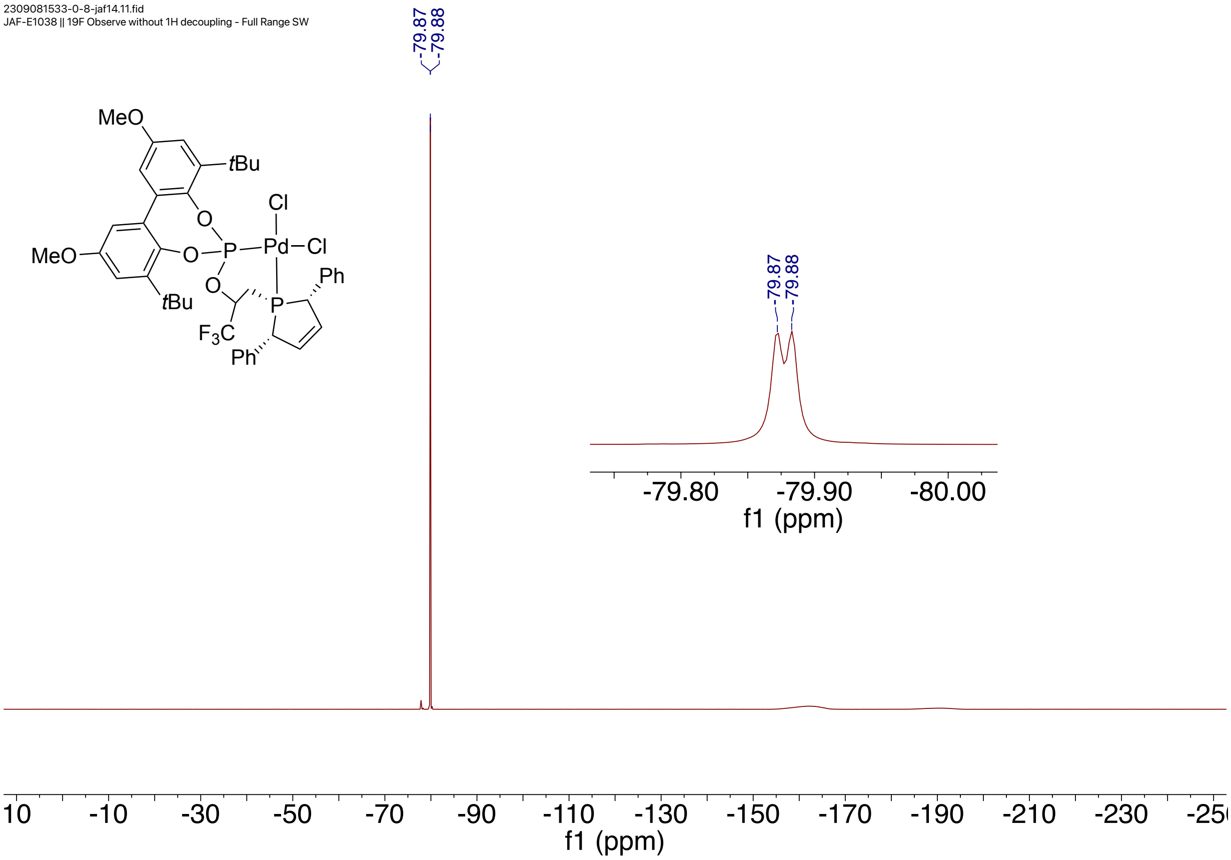
2309081533-0-8-jaf14.11.fid
JAF-E1038 || 19F Observe without 1H decoupling - Full Range SW
-79.87
-79.88
MeO
tBu
Cl
O
O–P–Pd–Cl
MeO
O
Ph
tBu
P
F3C
Ph
-79.87
-79.88
-79.80 -79.90 -80.00
f1 (ppm)
10 -10 -30 -50 -70 -90 -110 -130 -150 -170 -190 -210 -230
f1 (ppm)

11. References

18. Fuentes, J. A.; Janka, M. E.; Rogers, J.; Fontenot, K. J.; Bühl, M.; Slawin, A. M. Z.; Clarke, M. L. *Organometallics*, **2021**, 40, 3966-3978.

25. L. Hintermann, M. Schmitz, O. V. Matsev, P. Naumov. *Synthesis*, **2013**, *45*, 308-325.

38. How, R. C, Dingwall, P.; Hembre, R. T.; Ponasik, J. A.; Tolleson, G. S.; Clarke, M. L. *Mol. Catal.* **2017**, *434*, 116- 122.

46. Allian, A. D.; Garland, M. *Dalton. Trans.* **2005**, 1957.

53. C. Li, K. Xiong, L. Yan, M. Jiang, X. Song, T. Wang, X. Chen, Z. Zhan, Y. Ding, *Catal. Sci. Technol.* **2016**, *6*, 2143-2149.
